# Arylethynyl- or Alkynyl-Linked Pyrimidine and 7-Deazapurine 2′-Deoxyribonucleoside 3′-Phosphoramidites for Chemical Synthesis of Hypermodified Hydrophobic Oligonucleotides

**DOI:** 10.1021/acsomega.3c05202

**Published:** 2023-10-12

**Authors:** Ivana Jestřábová, Lenka Poštová Slavětínská, Michal Hocek

**Affiliations:** † Institute of Organic Chemistry and Biochemistry, Czech Academy of Sciences, Flemingovo nam. 2, CZ-16000 Prague 6, Czech Republic; ‡ Department of Organic Chemistry, Faculty of Science, Charles University, Hlavova 8, CZ-12843 Prague 2, Czech Republic

## Abstract

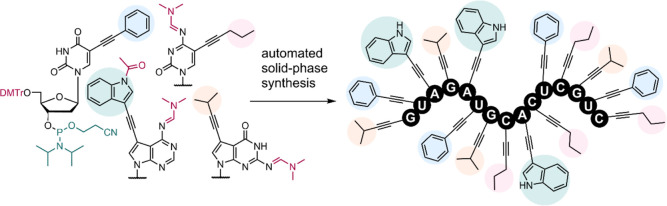

We designed and synthesized a set of 2′-deoxyribonucleoside 3′-phosphoramidites derived from 5-phenylethynyluracil, 5-(pentyn-1-yl)cytosine, 7-(indol-3-yl)ethynyl-7-deazaadenine, and 7-isopropylethynyl-7-deazaguanine. These nucleoside phosphoramidites were successfully used for automated solid-phase synthesis of oligonucleotides containing one or several modifications, including fully modified sequences where every nucleobase was displaying a modification, and their hybridization was studied. The phosphoramidite building blocks have potential for synthesis of hypermodified aptamers and other functional nucleic acid-based polymers, which sequence-specifically display amino acid-like hydrophobic substituents.

## Introduction

Base-modified oligonucleotides and nucleic acids find diverse applications^1^ in chemical biology, diagnostics, therapy, as well as in material science. Particularly interesting and promising are base-modified aptamers^2^ containing one^3^ or two^4^ aromatic or hydrophobic modifications targeting proteins with enhanced affinity and specificity compared to the corresponding unmodified oligonucleotides. The presence of 5-alkynylpyrimidine^5,6^ and 7-alkynyl-7-deazapurine^7^ in oligonucleosides (ONs) significantly stabilizes duplexes both with complementary DNA or RNA strands and hence alkynyl-modified ONs are used in antisense oligonucleotides (ASO),^5^ fluorescent hybridization probes,^8^ and many other applications.

In most of the above-mentioned studies, only one or two nucleobases were bearing the modifications while the others were unmodified.^3−8^ There were very few reports on enzymatic synthesis of fully modified (on all four nucleobases) nucleic acids,^9^ and so far the only recently reported application was in redox coding of DNA bases.^10^ An alternative way to prepare nucleic acids containing more base-modifications is ligation of short modified ONs.^11^ Previously, we reported^12^ the synthesis of a full set of all four 2′-deoxyribonucleoside triphosphates (dNTPs) bearing alkyl or aryl-groups linked through either rigid ethynyl or flexible alkyl tether to position 5 of pyrimidines (uracil or cytosine) and to position 7 of 7-deazapurines (7-deazaadenine and 7-deazaguanine) and their use in enzymatic synthesis of hypermodified DNA with DNA polymerases. Primer extension or asymmetric PCR was used^12^ to incorporate up to 150 modified nucleotides in a row, and later reverse transcription from RNA templates and ribonucleotide-containing primers was used^13^ to generate hypermodified single-stranded ONs. These monodispersed sequence-specific DNA polymers displaying four different hydrophobic small molecules can be re-PCRed and used for Sanger or NGS sequencing, and thus, they have potential in the selection of aptamers and other functional nucleic acids. However, in order to further explore their potential, we need to be able to also chemically synthesize these hypermodified ONs using automated solid-phase synthesis^14^ on a larger scale to produce micromolar quantities of material for biological testing. Therefore, we report here on the design and synthesis of a full set of protected base-modified 2′-deoxyribonucleoside 3′-phosphoramidites and their use in solid-phase synthesis of hypermodified ONs.

## Results and Discussion

### Synthesis of the Nucleoside Phosphoramidites

**Scheme 1:**
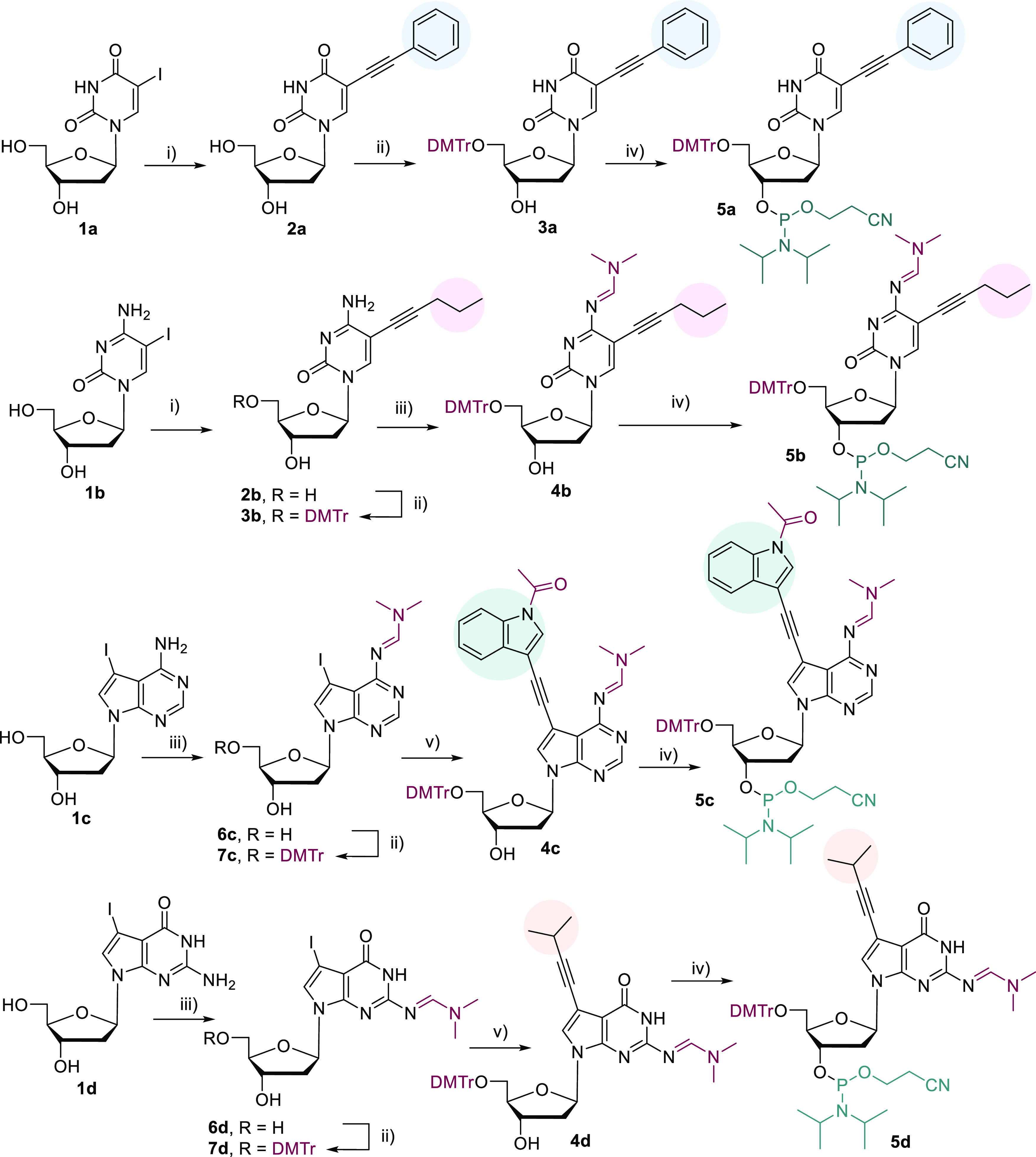
Reagents and conditions: (i) ethynyl benzene (10 equiv, for **1a**) or pent-1-yne (10 equiv, for **1b**), Pd(OAc)_2_ (0.1 equiv), CuI (0.1 equiv), TPPTS (0.1 equiv), TEA (6 equiv), MeCN/H_2_O (1:1), RT, under Ar, overnight (**2a** 91%, **2b** 91%); (ii) DMTrCl (1.2 equiv), DMAP (0.1 equiv), pyridine (dry), RT, overnight (**3a** 69%, **3b** 84%, **7c** 70%, **7d** 85%); (iii) DMF-DMA (14 equiv), DMF (dry), 40 °C, Ar, 4 h (**4b** 95%, **6c** 93%, **6d** 67%); (iv) 2-cyanoethyl-*N*,*N*-diisopropylchlorophosphoramidite (1.2 equiv), DIPEA (2.5 equiv), DCM (dry), 0 °C to RT, 1.5 h (**5a** 72%, **5b** 65%, **5c** 81%, **5d** 70%); (v) 1-acetyl-3-[(trimethylsilyl)ethynyl]indole (1.3 equiv, for **7c**) or 3-methylbut-1-yne (25 equiv, for **7d**), PdCl_2_(PPh_3_)_2_ (0.2 equiv), CuI (0.4 equiv), TEA (5 equiv), TEA·3HF (5 equiv, for **7c**), DMF, r.t., under Ar, overnight (**4c** 80%, **4d** 83%).

The design of the nucleoside phosphoramidite building blocks followed the same scheme as the previously reported set of base-modified dNTPs: 5-phenylethynyl-2-deoxyuridine, 5-pentyn-1-yl-2′-deoxycytidine, 7-(indol-3-yl)ethynyl-7-deaza-2′-deoxyadenosine, and 7-isopropylethynyl-7-deaza-2′-deoxyguanosine. The two arylethynyl substituents, phenylethynyl and (indol-3-yl)ethynyl, were designed to resemble Phe and Trp amino acid side chains, whereas the aliphatic isopropylethynyl and pentyn-1-yl groups are analogues of Val and Met side-chains. The synthesis built on our previous experience in the synthesis of the corresponding dNTPs using the Sonogashira cross-coupling reaction as a key step for attachment of the modification with additional steps for introduction of protecting groups and phosphoramidite moiety (Scheme 1).

The synthesis of modified pyrimidine nucleosides started by previously reported Sonogashira cross-coupling reactions of the corresponding iodinated nucleosides **1a** and **1b** with ethynylbenzene or pentyne in the presence of Pd(OAc)_2_, TPPTS, CuI, and TEA in H_2_O/MeCN (1:1) to give nucleosides **2a** (91%) and **2b** (91%).^12^ The protection of the 5′-hydroxyl group by dimethoxytrityl (DMTr) through the reaction with dimethoxytrityl chloride (DMTrCl) in the presence of *N*,*N*-dimethylaminopyridine (DMAP) afforded protected nucleosides **3a** (69%) and **3b** (84%), respectively. In the case of modified 2′-deoxycytidine, the reactive amino group was initially protected by a benzoyl-protecting group, but the reaction led to low yields (∼40%). Therefore, a different strategy was chosen using dimethylformamidine protecting group in analogy to our previous work.^15^ Reaction of **3b** with *N*,*N*-dimethylformamide dimethylacetal (DMF-DMA) in DMF at 40 °C gave compound **4b** in a good yield (95%). Finally, the reaction of **3a** and **4b** with 2-cyanoethyl-*N*,*N*-diisopropylchlorophosphoramidite and *N*,*N*-diisopropylethylamine (DIPEA) led to the desired fully protected nucleoside phosphoramidites **5a** (72%) and **5b** (65%).

In the case of modified 2′-deoxy-7-deazapurine nucleoside phosphoramidites, a different strategy was used. Since the aqueous Sonogashira cross-coupling reactions of 7-iodo-2′-deoxy-7-deazapurine nucleosides gave low yields, we decided to first protect the iodinated nucleosides and then perform the Sonogashira reactions in organic solvents. In the first step, we introduced dimethylformamidine protection of the amino groups through the reactions of 7-iodo-2′-deoxy-7-deazaadenosine **1c** or 7-deazaguanosine **1d** with DMF-DMA to give protected nucleosides **6c** (93%)^16^ and **6d** (67%), respectively. Consequently, 5′-hydroxyl groups were protected by DMTr groups to afford intermediates **7c** (70%)^7b^ and **7d** (85%) that were then used for the Sonogashira cross-coupling with the corresponding alkynes. For the introduction of the indolylethynyl moiety, we synthesized the known 1-acetyl-3-(trimethylsilyl)ethynylindole,^17^ which was then used in the Sonogashira cross-coupling reaction with the protected 7-iodo-2′-deoxy-7-deazaadenosine **7c**. The reaction was performed in the presence of TEA·3HF for *in situ* cleavage of TMS to release the terminal alkyne and in the presence of PdCl_2_(PPh_3_)_2_ catalyst, CuI, and TEA in DMF at ambient temperature overnight to give the desired modified nucleoside **4c** in a good yield of 80%. Different deprotection agents (KF or NH_4_F) were also attempted, but they led to complex mixtures of products. The Sonogashira cross-coupling of protected 7-iodo-2′-deoxy-7-deazaguanosine **7d** with isopropylacetylene afforded alkynylated nucleoside **4d** also in a good yield of 83%. The final conversion to the corresponding phosphoramidites, under the same conditions as those described above, resulted in building blocks **5c** (81%) and **5d** (70%). Interestingly, normal-phase flash chromatography did not purify the final compounds **5c** and **5d** from the oxidized reagent; thus, reverse-phase chromatography on C18 column was applied, leading to a successful purification. All of the reactions were gradually scaled up from 100 mg up to 7 g of the starting nucleosides to achieve sufficient amounts of the final phosphoramidite products (*ca.* 1 g of each).

### Solid-Phase Synthesis and Characterization of Modified Oligonucleotides

**Scheme 2:**
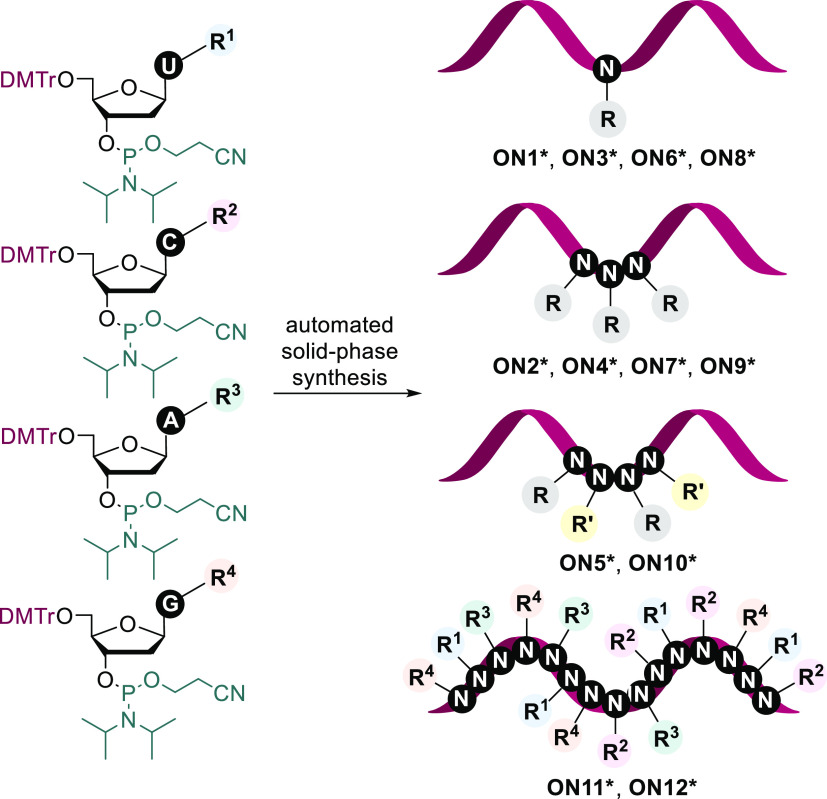
Solid-Phase Synthesis of the Partially and Hyper-Modified Oligonucleotides

The modified nucleoside phosphoramidite building blocks **5a**–**5d** were then used for the automated synthesis of modified oligonucleotides. We designed 12 target sequences containing one or three modified nucleotides in the middle, combination of two different modified nucleotides, and then fully modified ON strands containing combinations of all four modified nucleotides. The ONs were synthesized using a standard phosphoramidite synthesis protocol on an automated DNA synthesizer (Scheme 2, Table 1). The partially modified ON1*–ON10* were synthesized on standard solid-phase columns, while the hyper-modified ON11* and ON12* were synthesized on the universal solid-phase column. The 1 μmol scale was used with the 0.1 M concentration of all of the phosphoramidites. The coupling volume and duration for the natural phosphoramidites were 220 μL and 1 min 30 s, whereas for the modified phosphoramidites, the coupling time was increased to 6 min to increase the chance of successful incorporation. Following the synthesis, the cleavage of the ONs from the solid phase took place, followed by the deprotection of the residual protecting groups by aqueous ammonia. Then, the HPLC purification of the ONs was performed, followed by the mass characterization and purity control done by UHPLC–MS and concentration measurement by UV–vis spectrophotometer at 260 nm. For details, see Table 1 and Supporting Information.

**Table 1:** List of the Chemically Synthesized Modified Oligonucleotides ON1*–ON12*, Their Sequences, Calculated and Measured Masses [Da], Their Purities [%], and Isolated Yields [%]

code	sequence (5′→3′)	mass calc. [Da]	mass found [Da]	purity [%]	yield^a^ [%]
ON1*	ATCTCAGA*GAACTGC	4699.17	4699.03	100	65
ON2*	ATCTAGA*A*A*GACTGC	4999.61	4999.09	100	3 (11)^a^
ON3*	ATCTCAGG*AAGCTGC	4642.12	4642.71	100	64
ON4*	ATGTCAG*G*G*AGCTGC	4828.45	4827.80	84	13
ON5*	ATGTCG*A*G*A*AGCTGC	5023.67	5023.07	95	6 (10)^a^
ON6*	GCTCCGTC*GATTGAA	4634.10	4633.91	98	51
ON7*	GCTCCC*C*C*GATTGAA	4711.31	4710.97	100	42
ON8*	GCTCCGTU*GATTGAA	4669.19	4668.71	98	34
ON9*	GCTCCGU*U*U*ATTGAA	4816.28	4815.83	97	8 (12)^a^
ON10*	GCTCGC*U*C*U*ATTGAA	4847.39	4846.76	100	11 (17)^a^
ON11*	G*U*A*G*A*U*G*C*A*C*U*C*G*U*C*	5851.75	5851.11	90	6 (17)^a^
ON12*	G*A*C*G*A*G*U*G*C*A*U*C*U*A*C*	5912.83	5911.88	98	5 (20)^a^

a HPLC yields given in parentheses in case of lower isolated yield.

At first, each modified nucleotide was incorporated into a central position of the 15 nt ON sequence to verify the reactivity of the modified phosphoramidites **5a**–**5d**. In all cases, the modified ONs (ON1*, ON3*, ON6*, ON8*) were successfully synthesized, isolated by HPLC, and characterized by mass spectrometry. Next, the set of ONs containing three modified nucleotides in a row (ON2*, ON4*, ON7*, and ON9*) was also synthesized and characterized. Further, we successfully synthesized two ONs containing a combination of two different modified nucleotides (ON5*, ON10*). Finally, the two complementary hypermodified ONs containing all four modified nucleotides were successfully synthesized (ON11*, ON12*), although their isolation was more difficult as it was necessary to prolong the gradient up to 3 h (from 5 to 100% MeCN in aqueous 0.1 M TEAB solution, Clarity 5 μm Oligo XT reverse-phase column from Phenomenex) to obtain the pure ONs. Isolated yields (Table 1) varied from good 34–65% (for ONs containing one modification or three pentynylC modifications) to rather low yields (for most other ONs containing multiple modifications), but in all cases, we successfully isolated pure modified ONs in sufficient amounts for further studies. These results show that these hydrophobic and bulky phosphoramidites **5a**–**5d** are less efficient than standard phosphoramidites derived from cannonical nucleobases, in particular when incorporated into adjacent positions but still generally useful for the solid-phase synthesis of modified and hypermodified ONs.

**Figure 1:**
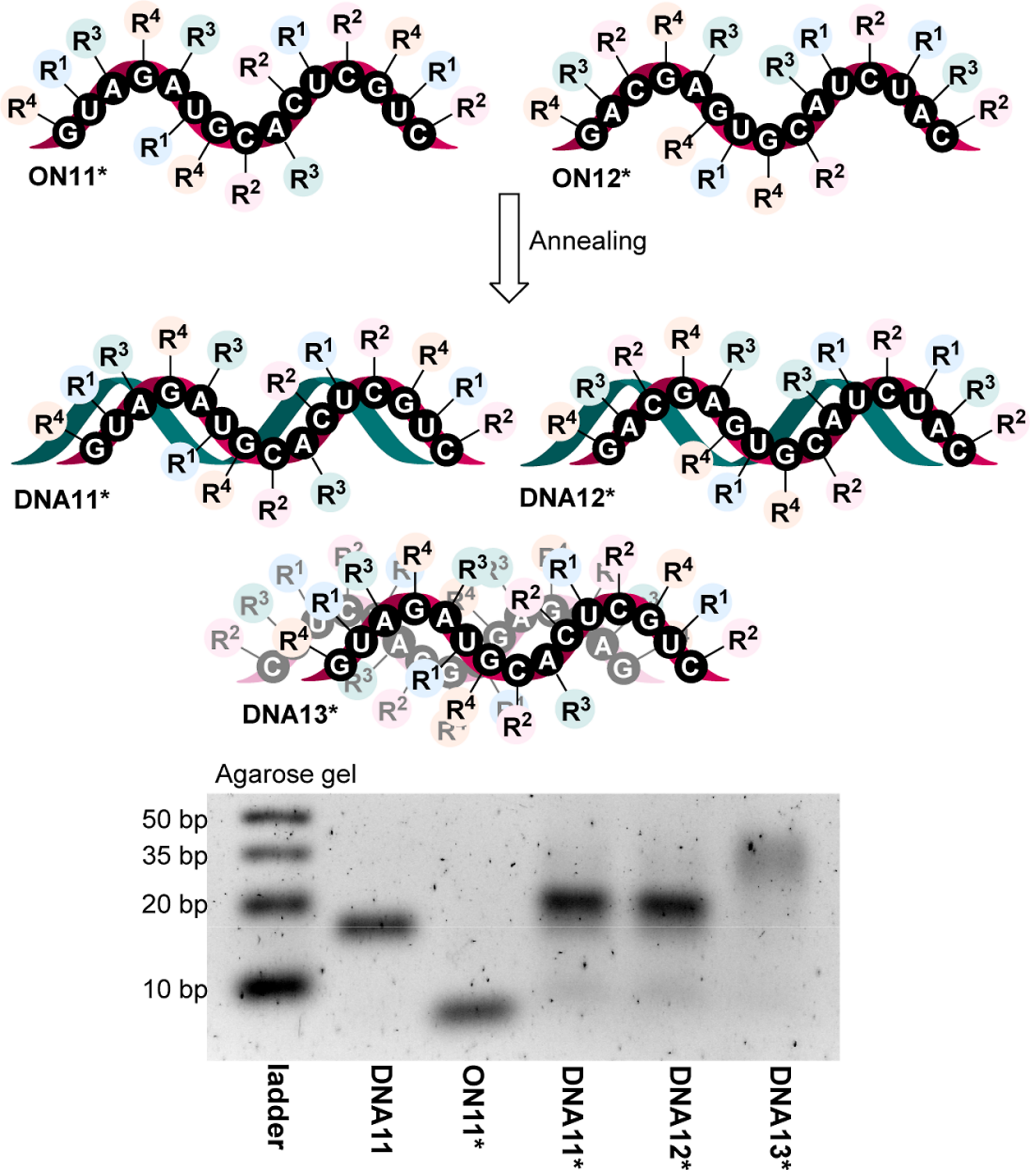
Annealing of the hyper-modified ONs* with either a nonmodified complementary strand or a hyper-modified complementary strand visualized on agarose gel (3%) with GelRed. Ladder: ultralow-range DNA ladder; **DNA11**: nonmodified ds-DNA with a sequence corresponding to modified DNA11*–DNA13*; ON11*: hyper-modified ss-ON11*; DNA11*, DNA12*: hyper-modified ON11*, ON12* annealed with the nonmodified complementary strands; DNA13*: annealed hyper-modified ON11* and ON12*.

After the successful synthesis and characterization of modified ON1*–ON12*, they were annealed with their complementary counterparts. First, the partially (ON1*–10*) and hyper-modified ONs* (ON11*, ON12*) were annealed with the corresponding complementary nonmodified strands to give dsDNA modified in one strand (DNA1*–DNA12*). Subsequently, the mutually complementary hypermodified ON11* and ON12* were annealed to one another to form dsDNA hyper-modified in both strands (DNA13*). Then, all resulting double-stranded DNA1*–13* were analyzed and visualized on native agarose gel electrophoreses (Figures 1 and S1 in Suppporting Information). Figure 1 shows somewhat slower mobility of dsDNA modified in one strand (DNA11* and DNA12*) compared to nonmodified double-stranded **DNA11**, while the modified ssON (ON11*) shows much faster mobility. The mobility of the DNA13* hypermodified in both strands is still much slower than those of any of the other ssONs or dsDNA due to their higher bulkiness. The lower intensity of the band of DNA13* could be explained by the presence of a higher number of 7-deazaguanine bases that are known^18^ to quench fluorescence of GelRed.

Finally, the annealing (*T*_a_) and denaturating (melting) temperatures (*T*_m_) of the modified dsDNAs were measured and compared with the nonmodified double-stranded DNAs to study the effect of the modification(s) on duplex stability. The results are summarized in Table 2, which shows that the introduction of one or three alkyne-modified adenine, guanine, or uracil (DNA1*–DNA5*, DNA8*, DNA9*) in the middle of the sequence has a rather destabilizing effect on the duplex, manifested by the decreasing *T*_m_. Conversely, incorporation of modified cytosine(s) leads to increased *T*_m_ and more stable duplexes (DNA6*, DNA7*, DNA10*). On the other hand, DNA11* and DNA12* hypermodified in one strand, as well as DNA13* hypermodified in both strands, show increased *T*_m_. These findings are in accord with our recent work on hypermodified DNA^12^ and previous papers describing a duplex stabilizing effect of multiple alkynyl-linked nucleobases^6,7^ and with other somewhat contradictory works^19^ reporting on the destabilizing effect of one alkynyl-linked uracil in DNA (strongly depending on the sequence context). Apparently, the stabilizing effect is generally observed in a higher density of alkynyl modifications where the increased π–π stacking prevails over some destabilizing effects of steric hindrance.

**Table 2:** Melting (*T*_m_) and Annealing Temperatures (*T*_a_) of Non-modified Double-Stranded DNA (DNA1–DNA11) and Double-Stranded Modified DNA (DNA1*–DNA13*) Determined by UV–Vis Spectroscopy^a^

DNA*^b^	DNA^c^*T*_m_ [°C]	DNA* *T*_m_ [°C]	DNA* *T*_a_ [°C]	Δ*T*_m_/modification
DNA1*	51.8	49.6	43.8	–2.2
DNA2*	47.6	44.4	40.3	–1.1
DNA3*	55.9	54.7	50.2	–1.2
DNA4*	59.8	57.9	54.2	–0.6
DNA5*	58.6	55.7	50.9	–0.7
DNA6*	57.3	57.5	52.4	+0.2
DNA7*	57.4	64.6	59.9	+2.4
DNA8*	53.3	52.2	47.1	–1.1
DNA9*	48.5	47.5	42.8	–0.3
DNA10*	52.3	58.6	52.4	+1.6
DNA11*	54.9^d^	62.0	56.3	+0.5
DNA12*	d	62.3	56.4	+0.5
DNA13*	d	61.5	56.2	+0.2

a In the last column is the difference of *T*_m_ per modification in comparison with non-modified DNA *T*_m_.

b DNA1*–DNA12* are dsDNA made by annealing of base-modified ON1*–ON12* with the corresponding nonmodified complementary strand, while the DNA13* was made by annealing of two modified strands ON11* and ON12*.

c **DNA1–DNA11** are the corresponding nonmodified double-stranded DNA sequences.

d Nonmodified **DNA11** corresponds to the same sequence as the modified DNA11*–DNA13*.

## Conclusions

In conclusion, we designed and synthesized a full set of all four 2′-deoxyribonucleoside 3′-phosphoramidites derived from 5-substituted pyrimidines and 7-substituted 7-deazapurines, each bearing a different hydrophobic arylethynyl or alkylethynyl modification. These phosphoramidites are useful building blocks for the automated solid-phase synthesis of modified or hypermodified ONs, although the synthesis of hypermodified ONs gives rather low isolated yields. We synthesized several ONs bearing different numbers of modified bases and studied their hybridization. Interestingly, a single alkynyl modification incorporated in the middle of the sequence has mostly a destabilizing effect on the DNA duplex, whereas a high density of alkynyl-linked nucleobases stabilizes the duplexes. The phosphoramidite building blocks will be further used in the chemical synthesis of truncated versions of modified aptamers and other functional DNA oligomers and polymers.

## Experimental Section

For detailed procedures and characterization of compounds, see the Supporting Information.

### General Method A: Dimethoxytritylation of 5′-OH

The precursor was dried by several coevaporations with anhydrous pyridine (3 × 5 mL) and finally dissolved in anhydrous pyridine along with *N*,*N*-dimethylaminopyridine (DMAP, 0.1 equiv). Solution of 4,4′-dimethoxytrityl chloride (DMTrCl, 1.2 equiv) in anhydrous pyridine was added in 4 portions over 1 h, and the reaction was stirred at room temperature overnight. The solvent was removed under reduced pressure, and the crude was redissolved in DCM, washed with 10% aqueous solution of NaHCO_3_, brine, and finally dried over anhydrous Na_2_SO_4_. Purification by high-performance flash chromatography (HPFC; usually DCM/MeOH 0–1% with 0.5% Et_3_N) afforded the desired compound.

### General Method B: Dimethylformamidine Protection of Nucleobase Amino Group

To the amino nucleoside precursor dissolved in anhydrous DMF under an argon atmosphere was added dimethylformamide dimethylacetal (DMF-DMA, 14 equiv), and the reaction was stirred for 4 h at 40 °C. Subsequently, the solvent was evaporated, and the crude product was purified by HPFC (DCM/MeOH).

### General Method C: Synthesis of 3′-Phosphoramidites

Protected nucleoside was dried by repeated coevaporation with anhydrous pyridine (3 × 5 mL), followed by coevaporation with anhydrous DCM (3 × 5 mL), and dried under vacuum for 30 min. Subsequently, the starting material was dissolved in anhydrous DCM in a sealed flask under an argon atmosphere with molecular sieves (4 Å). Subsequently, the reaction was cooled down to 0 °C and freshly distilled *N*,*N*-diisopropylethylamine (DIPEA) was added followed by the addition of 2-cyanoethyl-*N*,*N*-diisopropylchlorophosphoramidite. The mixture was then warmed to room temperature and stirred until a complete conversion was observed by TLC analysis (cyclohexane/EtOAc, approximately 1.5 h). Then, the mixture was diluted with anhydrous DCM, quickly washed under an argon atmosphere with saturated aqueous solution of KI and dried over Na_2_SO_4_. Purification was done by normal-phase flash chromatography (cyclohexane/EtOAc with 0.5% TEA) under an argon atmosphere (compounds **5a** and **5b**) or reverse-phase HPFC (H_2_O/MeCN 9:1 to 100% MeCN, compounds **5c** and **5d**) provided the final compound usually as a mixture of two diastereomers.

### Solid-Phase Synthesis of Oligonucleotides

Synthesis of partially and hyper-modified oligonucleotides ON1*–ON12* with the phosphoramidites **5a**, **5b**, **5c**, and **5d** was performed on a 1 μmol scale. The trityl-off mode was used to prevent the loss of the products in another purification round (which was shown to be crucial with the hypermodified ON11*, ON12*, where the yield of the solid-phase synthesis was rather low) and also to avoid the risk of a strong attachment to the reverse phase and possible problems with the elution due to the increased hydrophobicity of the modified ONs. For partially modified ON1*–ON10*, standard solid-phase columns were used; for hyper-modified ONs* (ON11*, ON12*) the universal solid-phase columns were used. Each phosphoramidite was diluted to a 0.1 M solution and 0.3 M 5-(benzylthio)-1*H*-tetrazole (BTT) solution in MeCN was used as an activator. Iodine solution (0.02 M) in THF/pyridine/water (ratio 70:20:10) was used for the oxidation step. Standard cycle procedures provided by BioAutomation Corporation were applied for the unmodified as well as modified phosphoramidites. The coupling volume and duration for the natural phosphoramidites were 220 μL and 1 min 30 s, whereas for the modified phosphoramidite the coupling time was increased to 6 min to maximize the incorporation efficiency. Cleavage from the solid-phase was performed by 30% aqueous NH_3_ for 2 × 45 min (2 × 1 mL). Following this, a deprotection step was carried out by incubation of the oligonucleotide solutions at 65 °C for 6 h.

### Purification and Characterization of Oligonucleotides

The purification of the oligonucleotides was performed using HPLC with a linear gradient of MeCN (0–100%) in 0.1 M triethylammonium bicarbonate (TEAB) buffer (pH 7.6). The final lyophilization from H_2_O provided pure products. The approximate concentrations were measured by UV/vis spectrophotometer at 260 nm, mass and purity were then measured on UPLC–MS. Most of the modified oligonucleotides were >90% pure, except ON4* showing 84% purity. The sequences, calculated and measured masses, purities, and yields of the chemically synthesized oligonucleotides are shown in Table 1. The exact concentrations and isolated yields of the modified ON*s were calculated based on the phosphorus content determined by elemental analysis of aqueous solutions of isolated pure ONs.

## Supporting Information Available

Synthetic protocols and characterization of new compounds (PDF)
