# Supplementary material for: Arylethynyl- or Alkynyl-Linked Pyrimidine and 7-Deazapurine 2′-Deoxyribonucleoside 3′-Phosphoramidites for Chemical Synthesis of Hypermodified Hydrophobic Oligonucleotides
Source: ACS Omega. 2023 Oct 12;8(42):39447–53. doi: 10.1021/acsomega.3c05202 (PMC10601081; doi:10.1021/acsomega.3c05202)
Supplement: Supplementary file 1 — ao3c05202_si_001.pdf [file ao3c05202_si_001.pdf]

SUPPLEMENTARY INFORMATION

Arylethynyl- or alkynyl-linked pyrimidine and 7-deazapurine 2'-deoxyribonucleoside 3'-phosphoramidites for chemical synthesis of hypermodified hydrophobic oligonucleotides

Ivana Jestřábová,[a,b] Lenka Poštová Slavětínská,[a] Michal Hocek*[a,b]

a) Institute of Organic Chemistry and Biochemistry, Czech Academy of Sciences, Flemingovo nam. 2, CZ-16000 Prague 6, Czech Republic; hocek@uochb.cas.cz

b) Department of Organic Chemistry, Faculty of Science, Charles University, Hlavova 8, CZ-12843 Prague 2, Czech Republic

Contents

1 General Remarks 3

2 Synthesis of Phosphoramidites 4

2.1 Experimental Procedures 4

2.1.1 Synthesis of Published Compounds 4

2.1.2 General Method A: Dimethyltritylation of 5′-OH 4

2.1.3 General Method B: Dimethylformimidamide Protection of Nucleobase Amino Group 5

2.1.4 General Method C: Synthesis of 3′-Phosphoramidites 5

2.1.5 Synthesis of Compound 1d 6

2.1.6 Synthesis of Modified 2′-Deoxyuridine 8

2.1.7 Synthesis of Modified 2′-Deoxycytidine 11

2.1.8 Synthesis of Modified 2′-Deoxyadenosine 16

2.1.9 Synthesis of Modified 2′-Deoxyguanosine 19

3 Synthesis of Oligonucleotides 24

3.1 Solid-phase Synthesis 24

3.2 Purification and Characterization 24

3.3 Annealing 25

3.4 Measurement of Melting Temperatures 28

4 Copies of Chromatograms, Spectra and Graphs 30

4.1 Absorbance Chromatograms of Oligonucleotides 30

4.2 ESI Spectra of Oligonucletotides 36

4.3 Melting and Annealing Curves 48

4.4 Copies of NMR Spectra 51

5 Reference List 79

1 General Remarks

All chemicals used were purchased from common commercial suppliers, such as Fluorochem, Sigma Aldrich and Enamine. Reactions were monitored by silica gel thin-layer chromatography (TLC) in Merck silica gel 60 F_{254} plates with UV light detection (254 and 365 nm) combined with visualisation by the solution of 4-anisaldehyde in ethanol with sulphuric acid (10%). The masses of individual spots on TLC plate were measured by Advion Expression Compact Mass Spectrometer connected with Plate Express TLC Plate Reader (TLC-MS) using electrospray ionization (ESI). Solvents were removed *in vacuo* with the bath temperature between 40–60 °C. Purification of nucleosides was carried out on CombiFlash Rf+ (Teledyne Isco) with columns filled with Silicagel 40–63 μm from VWR International or C18-HP 30 μm from Interchim.

Purity of all compounds was determined by NMR and HR-MS spectra. ^{1}H and ^{13}C NMR spectra were measured on Bruker Avance III HD 500 MHz (^{1}H at 500.0 MHz, ^{13}C at 125.7 MHz and ^{31}P at 202.4 MHz) and JEOL ECZR 500 MHz (^{1}H at 500.2 MHz, ^{13}C at 125.8 MHz and ^{31}P at 202.5 MHz) in DMSO-*d6*, D_2O or CD_3CN referenced to the residual solvent signal. Chemical shifts are given in ppm (δ-scale), coupling constants (J) in Hz. Complete assignment of all NMR signals was performed using a combination of H,H-COSY, H,C-HSQC and H,C-HMBC experiments. MestreNova (version 14) from Mestrelab Research was used for data evaluation. Low and high resolution mass spectra of small molecules were measured on LTQ Orbitrap XL spectrometer (ESI ionization, Thermo Fisher Scientific). The matrix (1 μL) was applied to the target (ground steel) and dried down at room temperature. Mass of the small molecules were acquired by the MS service at IOCB.

Reagents and solvents for the solid-phase synthesis of oligonucleotides were purchased from Sigma-Aldrich, Link Technologies, and Thermo Fisher-Scientifics. Modified oligonucleotides were synthesized through standard phosphoramidite chemistry with an automated DNA synthesizer (Mermade 8, BioAutomation Corporation) either on standard or universal solid-phase columns (500 Å). Purification of the prepared oligonucleotides was performed using semi-preparative HPLC (Waters modular HPLC system) on a column packed with C18 reverse phase (Clarity 5 μm Oligo XT 100Å from Phenomenex for the hypermodified oligonucleotides (ONs) and Kinetex 5 μm EVO C18 100 Å from Phenomenex for the partially modified ONs). The analysis of ONs was performed by UHPLC-MS-ESI (Agilent 1290 Infinity II Bio LC System with DAD

detector and mass spectrometer MSD XT). The analysis was carried out according to standard procedures using mobile phases A (12.2 mM TEA (triethylamine), 300 mM HFIP (hexafluoro-2-propanol) in H_2O) and B (12.2 mM Et_3N, 300 mM HFIP in H_2O in 100% MeOH) by 20 min gradient from 5% B to 50% B in A for the partially modified ONs and by 22 min gradient from 5% B to 100% B in A for the fully modified ONs using BioZen 1.7 μm oligo column 2.1×50 mm (Phenomenex). The absorbance chromatograms were analyzed in MestreNova and the LC-ESI-MS were deconvoluted using free software UniDec[1]. Approximate concentrations of ONs solutions were calculated using the on-line tool at https://www.atdbio.com/tools/oligo-calculator and A_{260} values measured on a Cary100 Bio UV/VIS Spectrophotometer (Varian) and/or on Nanodrop 1000 (Thermo Fischer Scientific). The exact concentrations and yields were determined based on the phosphorus content measured by the elemental analysis of pure ONs. The non-modified oligonucleotides were ordered from Eurofins.

The agarose gels were visualized by GelRed (Biotium) using Typhoon FLA 9500 (GE Healthcare Life Sciences). Melting and annealing temperatures were measured by UV-absorption measurements which were performed on Cary 100 Bio UV/VIS Spectrophotometer with temperature controller (Varian).

2 Synthesis of Phosphoramidites

2.1 Experimental Procedures

2.1.1 Synthesis of Published Compounds

Compounds **2a** and **2b** were synthesized according to the reported procedures[2,6] from corresponding iodonucleosides **1a** and **1b**. 1-Acetyl-3-[(trimethylsilyl)ethynyl]indole was prepared following the published procedures[3-5] starting from 1*H*-indole. Compound **7c** was prepared by a multi-step synthesis reported in literature[6-9] from 4-chloro-7*H*-pyrrolo[2,3-d]pyrimidine. Compound **8** was prepared according to the published synthesis[10] from 4-chloro-7*H*-pyrrolo[2,3-d]pyrimidin-2-amine.

2.1.2 General Method A: Dimethoxytritylation of 5′-OH

Precursor was dried by several co-evaporations with anhydrous pyridine (3×5 mL) and finally dissolved in anhydrous pyridine along with *N*,*N*-dimethylaminopyridine

(DMAP, 0.1 equiv.). Solution of 4,4′-dimethoxytrityl chloride (DMTrCl, 1.2 equiv.) in anhydrous pyridine was added in 4 portions over 1 h and the reaction was stirred at room temperature overnight. The solvent was removed under reduced pressure and the crude was re-dissolved in DCM, washed with 10% aqueous solution of $NaHCO_3$, brine, and finally dried over anhydrous Na_2SO_4. Purification by high-performance flash chromatography (HPFC; usually DCM/MeOH 0–1% with 0.5% Et_3N) afforded the desired compound.

2.1.3 General Method B: Dimethylformamidine Protection of Nucleobase Amino Group

To the amino nucleoside precursor dissolved in anhydrous DMF under argon atmosphere, dimethylformamide dimethylacetal (DMF-DMA, 14 equiv.) was added and the reaction was stirred for 4 hours at 40 °C. Subsequently, the solvent was evaporated and the crude product was purified by HPFC (DCM/MeOH).

2.1.4 General Method C: Synthesis of 3′-Phosphoramidites

Protected nucleoside was dried by repeated co-evaporation with anhydrous pyridine (3×5 mL), followed by co-evaporation with anhydrous DCM (3×5 mL), and dried under vacuum for 30 min. Subsequently, the starting material was dissolved in anhydrous DCM in a sealed flask under argon atmosphere with molecular sieves (4 Å). Subsequently, the reaction was cooled down to 0 °C and freshly distilled *N*,*N*-diisopropylethylamine (DIPEA) was added followed by the addition of 2-cyanoethyl-*N*,*N*-diisopropylchlorophosphoramidite. The mixture was then warmed up to room temperature and stirred until a complete conversion was observed by TLC analysis (cyclohexane/EtOAc, approx. 1.5 h). Then, the mixture was diluted with anhydrous DCM, quickly washed under an argon atmosphere with saturated aqueous solution of KI and dried over Na_2SO_4. Purification was done by normal-phase flash chromatography (cyclohexane/EtOAc with 0.5% TEA) under argon atmosphere (compounds **5a** and **5b**) or reverse-phase HPFC (H_2O/MeCN 9:1 to 100% MeCN, compounds **5c** and **5d**) provided final compound usually as a mixture of two diastereomers.

2.1.5 Synthesis of Compound 1d

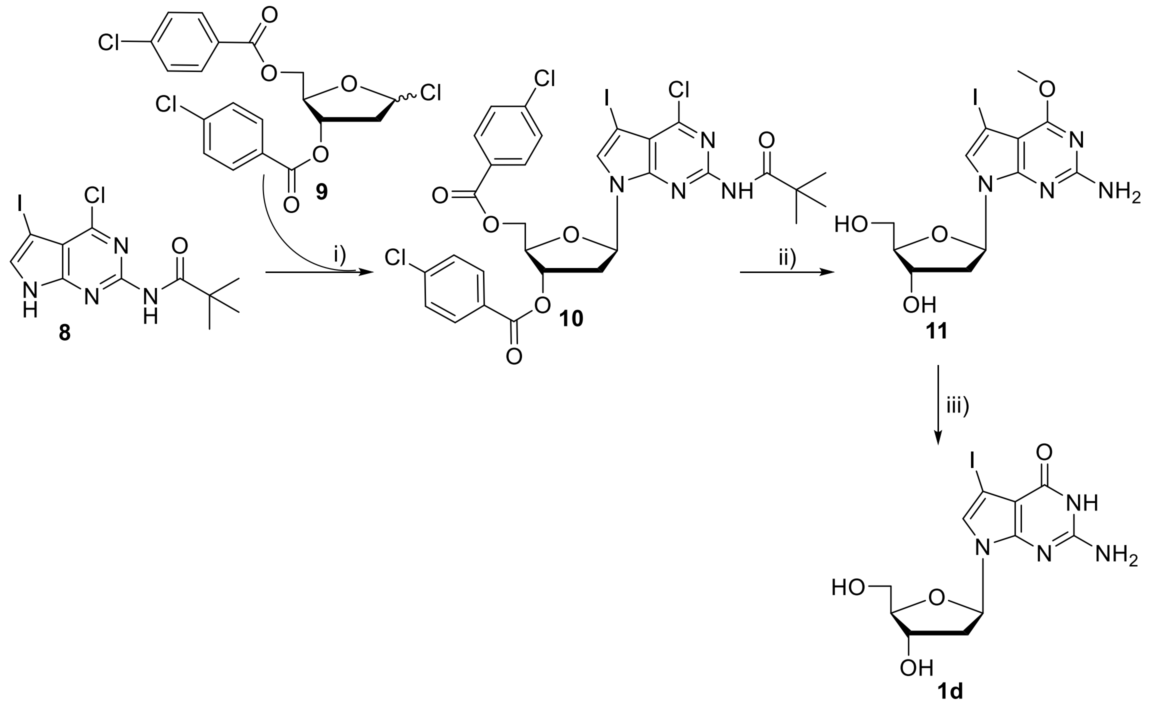

Scheme S1 Reagents and conditions: i) 3,5-*O*-bis(4-chlorobenzoyl)-2-deoxy-*α*-d-ribofuranosyl chloride (**9**, 1.3 equiv.), KOH (2.5 equiv.), TDA-1 (0.07 equiv.), dry MeCN, RT, 2 h; ii) 0.5 M MeONa (10 equiv.), reflux, 2.5 h; iii) 2 M NaOH (160 equiv.), reflux, 4 h;

_N_-[7-[3,5-Bis-_O_-(4-chlorobenzoyl)-2-deoxy-β-D-deoxyribofuranosyl]-4-chloro-5-iodo-7_H_-pyrrolo[2,3-_d_]pyrimidin-2-yl]-2,2-dimethylpropanamide (10)

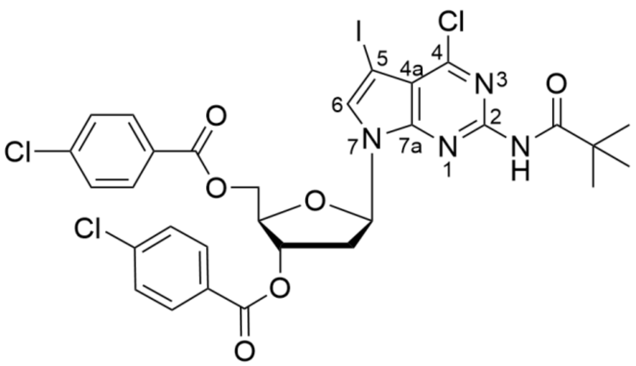

Powdered KOH (3.7 g, 66.0 mmol) was stirred in MeCN (600 mL) for 3 min after which tris[2-(2-methoxyethoxy)ethyl]amine (TDA-1; 591 μL, 1.8 mmol) was injected and the mixture was stirred for 20 min. Subsequently, *N*-(4-chloro-5-iodo-7*H*-pyrrolo[2,3-*d*]pyrimidin-2-yl)pivalamide **8** (10 g, 26.4 mmol) was added followed by stirring for another 20 min. After adding 3,5-*O*-bis(4-chlorobenzoyl)-2-deoxy-α-d-ribofuranosyl chloride **9** (14.8 g, 34.3 mmol), the stirring continued for 5 min until the precipitation of the product **10** started. The precipitate was then filtered, washed with MeCN and dried to result in the pure compound **10** (20.3 g, 99%) as a white solid.

^{1}H NMR (400.1 MHz, DMSO-d$_6$): 1.18 (s, 9H, (CH$_3$)$_3$C); 2.71 (m, 1H, H-2´a); 3.18 (m, 1H, H-2´b); 4.48 – 4.58 (m, 2H, H-4´,5´a); 4.68 (m, 1H, H-5´b); 5.79 (m, 1H, H-3´); 6.61 (t, 1H, $J_{1´,2´b} = J_{1´,2´a}$ = 7.1 Hz, H-1´); 7.58 and 7.64 (2×m, 2×2H, H-*m*-Ph); 7.88 (vbs, 1H, H-6); 7.96 and 8.05 (2×m, 2×2H, H-*o*-Ph); 10.28 (bs, 1H, NH).

^{13}C NMR (100.6 MHz, DMSO-d$_6$): 27.38 ((**C**H$_3$)$_3$C); 35.54 (CH$_2$-2´); 39.6 ((CH$_3$)$_3$**C**, *overlap solvent*); 53.90 (C-5); 64.43 (CH$_2$-5´); 75.47 (CH-3´); 81.14 (CH-4´); 83.54 (CH-1´); 128.16 (C-*i*-Ph); 128.96 and 128.99 (CH-*m*-Ph); 131.07 and 131.31 (CH-*o*-Ph); 138.44 and 138.59 (C-*p*-Ph); 164.46 and 164.62 (COO); 177.08 (CONH). *Carbon signals of C-2,4,4a,7a and CH-6 were not detected*

HR ESI-MS calculated m/z: 771.00354 [M+H]+, 792.98548 [M+Na]+, found m/z: 771.00363 [M+H]+, 792.98562 [M+Na]+.

2-Amino-6-methoxy-7-iodo-9-(β-D-2'-deoxyribofuranosyl)-7-deazapurine (11)

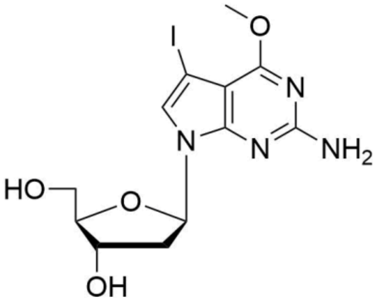

Compound **11** was prepared according to a published procedure.[11] Mixing compound **10** (12.2 g, 15.8 mmol) with 0.5 M MeONa in MeOH (305 mL) and following the described procedure led to the pure product **11** (4.5 g, 70%). NMR and MS spectra were in accord with the literature.[11]

7-Iodo-2′-deoxy-7-deazaguanosine (1d)

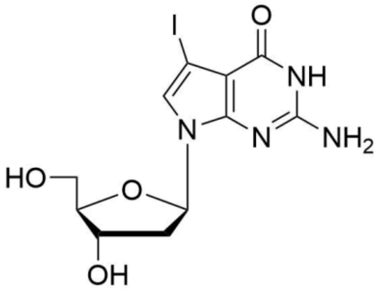

Compound **1d** was prepared according to the published procedure.[12] Compound **11** (4.5 g, 11.1 mmol) was mixed with 2 M NaOH aqueous solution (880 mL) and by following the published procedure pure compound **1d** (4.3 g, 99%) was acquired as a white solid. NMR and MS spectra were in accord with the literature.[12]

2.1.6 Synthesis of Modified 2′-Deoxyuridine

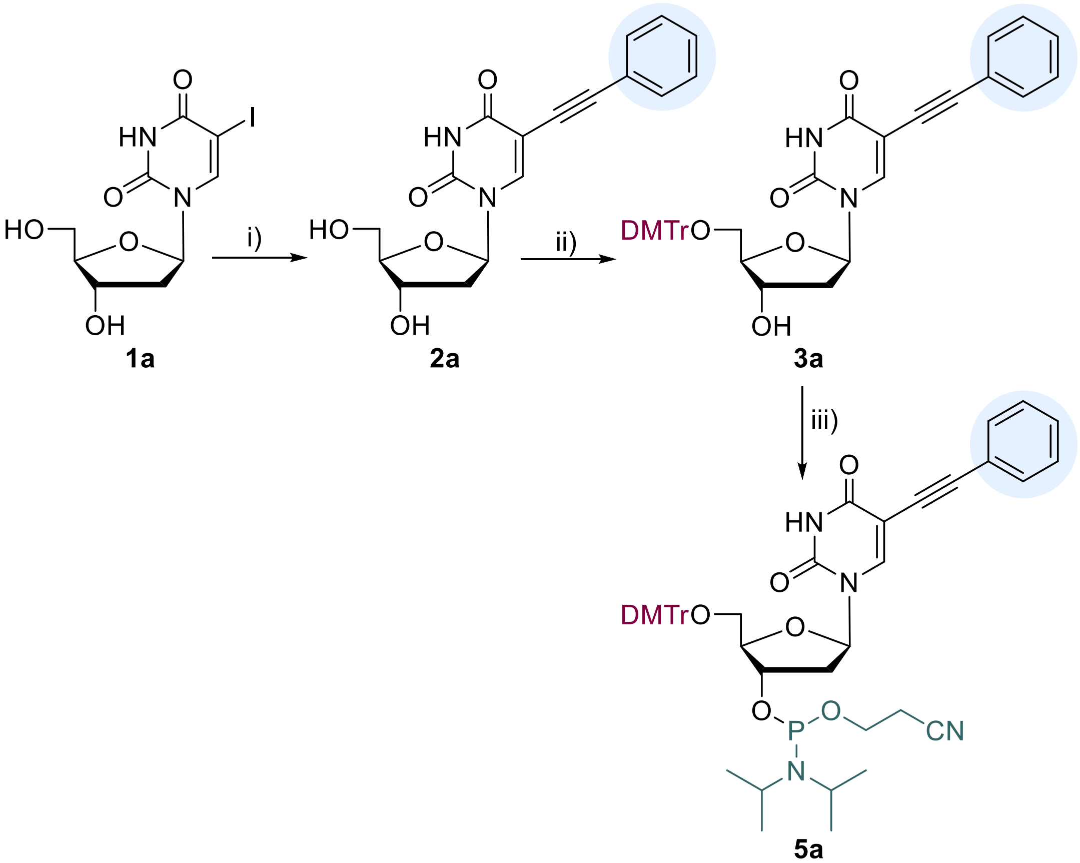

Scheme S2 Reagents and conditions: i) ethynyl benzene (10 equiv), $Pd(OAc)_2$ (0.1 equiv.), CuI (0.1 equiv.), TPPTS (0.1 equiv.), TEA (6 equiv.), $MeCN/H_2O$ (1:1), RT, Ar, overnight; ii) DMTrCl (1.2 equiv.), DMAP (0.1 equiv.), pyridine (dry), RT, overnight; iii) 2-cyanoethyl-*N*,*N*-diisopropylchlorophosphoramidite (1.2 equiv.), DIPEA (2.5 equiv.), DCM (dry), 0 °C to RT, 1.5 h;

5'-*O*-[Bis(4-methoxyphenyl)phenylmethyl]-5-(2-phenylethyn-1-yl)-2′-deoxyuridine (3a)

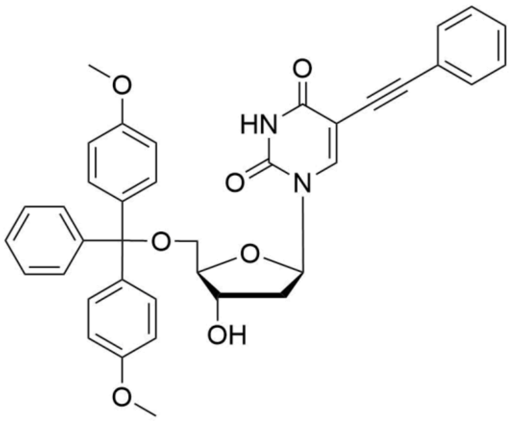

To synthesize compound **3a** General method A was used. To the solution of compound **2a** (130 mg, 0.40 mmol) in dry pyridine (2.3 mL), DMAP (4.9 mg, 0.04 mmol) and solution of DMTrCl (161 mg, 0.48 mmol) in pyridine (1.2 mL) were added. The compound **3a** (172 mg, 69%) was afforded as a yellowish solid.

^{1}H NMR (500.2 MHz, DMSO-d_6): 2.24 (ddd, 1H, J_{gem} = 13.5, $J_{2'b,1'}$ = 6.2, $J_{2'b,3'}$ = 3.4, H-2′b); 2.30 (ddd, 1H, J_{gem} = 13.5, $J_{2'a,1'}$ = 7.4, $J_{2'a,3'}$ = 6.2, H-2′a); 3.16 (dd, 1H, J_{gem} = 10.6, $J_{5'b,4'}$ = 2.7, H-5′b); 3.23 (dd, 1H, J_{gem} = 10.6, $J_{5'a,4'}$ = 5.0, H-5′a); 3.65, 3.66 (2 × s, 2 × 3H, CH_3O-DMTr); 3.96 (ddd, 1H, $J_{4',5'}$ = 5.0, 2.7, $J_{4',3'}$ = 3.4, H-4′); 4.31 (dt, 1H, $J_{3',2'}$ = 6.2, 3.4, $J_{3',4'}$ = 3.4, H-3′); 5.34 (bs, 1H, OH-3′); 6.15 (dd, 1H, $J_{1',2'}$ = 7.4, 6.2, H-1′); 6.81 – 6.86 (m, 4H, H-*m*-C_6H_4OMe-DMTr); 7.12 – 7.14 (m, 2H, H-*o*-Ph); 7.16 (m, 1H, H-*p*-C_6H_5-DMTr); 7.25 – 7.36 (m, 9H, H-*m,p*-Ph, H-*o*-C_6H_4OMe-DMTr, H-*m*-C_6H_5-DMTr); 7.40 – 7.43 (m, 2H, H-*o*-C_6H_5-DMTr); 8.06 (s, 1H, H-6).

^{13}C NMR (125.8 MHz, DMSO-d_6): 40.40 (CH_2-2′); 55.11 (CH_3O-DMTr); 63.78 (CH_2-5′); 70.65 (CH-3′); 82.08 (**C**≡C-Ph); 85.33 (CH-1′); 86.07 (C-DMTr); 86.23 (CH-4′); 92.08 (C≡**C**-Ph); 98.70 (C-5); 113.38, 113.39 (CH-*m*-C_6H_4OMe-DMTr); 122.40 (C-*i*-Ph); 126.86 (CH-*p*-C_6H_5-DMTr); 127.81 (CH-*o*-C_6H_5-DMTr); 128.05 (CH-*m*-C_6H_5-DMTr); 128.60 (CH-*m*-Ph); 128.65 (CH-*p*-Ph); 129.78, 129.85 (CH-*o*-C_6H_4OMe-DMTr); 131.23 (CH-*o*-Ph); 135.54, 135.74 (C-*i*-C_6H_4OMe-DMTr); 143.02 (CH-6); 144.85 (C-*i*-C_6H_5-DMTr); 149.56 (C-2); 158.23, 158.24 (C-*p*-C_6H_4OMe-DMTr); 161.65 (C-4).

HR ESI-MS calculated m/z: 631.24388 $[M+H]^+$, 653.22582 $[M+Na]^+$, found m/z: 631.24400 $[M+H]^+$, 653.22571 $[M+Na]^+$.

5'-*O*-[Bis(4-methoxyphenyl)phenylmethyl]-5-(2-phenylethyn-1-yl)-2′-deoxyuridine-3′-(2-cyanoethyl *N,N*-diisopropylphosphoramidite) (5a)

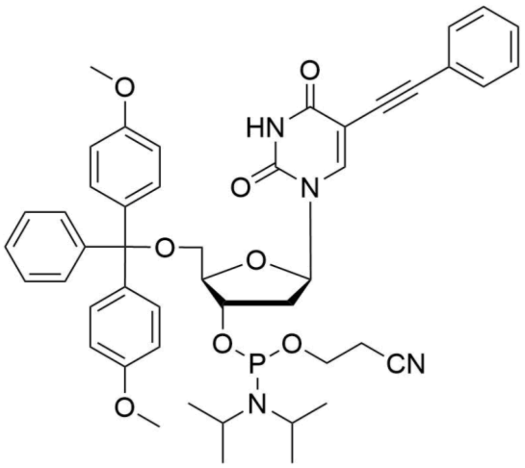

Compound **5a** was prepared using General method C by dissolution of the precursor **3a** (1.1 g, 1.74 mmol) in dry DCM (10 mL) and addition of *N*,*N*-diisopropylethylamine (DIPEA, 760 μL, 4.36 mmol) and 2-cyanoethyl-*N*,*N*-diisopropylchlorophosphoramidite (584 μL, 2.62 mmol). Purification by flash chromatography (EtOAc/cyclohexane 1:1 with 0.5% TEA) provided the final compound **5a** (1.0 g, 72%) as a 1:1 mixture of two diastereomers in a form of a white foam.

^{1}H NMR (500.0 MHz, CD_3CN): 1.07 (1×d, 2×3H, $J_{CH3,CH}$ = 6.8 Hz, (C**H$_3$**)$_2$CH); 1.16 (d, 2×3H, $J_{CH3,CH}$ = 7.1 Hz, (C**H$_3$**)$_2$CH); 1.165 (d, 2×3H, $J_{CH3,CH}$ = 6.8 Hz, (C**H$_3$**)$_2$CH); 1.173 (d, 2×3H, $J_{CH3,CH}$ = 7.1 Hz, (C**H$_3$**)$_2$CH); 2.37 – 2.58 (m, 2×2H, H-2´); 2.53 and 2.65 (2×t, 2×2H, $J_{CH2,CH2}$ = 6.0 Hz, OCH$_2$C**H$_2$**CN); 3.27 – 3.39 (m, 2×2H, H-5´); 3.67 (s, 2×3H, CH$_3$O-DMTr); 3.676 and 3.679 (2×s, 2×3H, CH$_3$O-DMTr); 3.51 – 3.68 (m, 4×1H, (CH$_3$)$_2$C**H**); 3.65 – 3.86 (m, 2×2H, OC**H$_2$**CH$_2$CN); 4.14 and 4.19 (2×bq, 2×1H, $J_{4´,5´b}$ = $J_{4´,5´a}$ = $J_{4´,3´}$ = 3.3 Hz, H-4´); 4.60 – 4.70 (m, 2×1H, H-3´); 6.18 (bt, 1H, $J_{1´,2´a}$ = $J_{1´,2´b}$ = 6.5 Hz, H-1´); 6.20 (dd, 1H, $J_{1´,2´a}$ = 7.2 Hz, $J_{1´,2´b}$ = 6.1 Hz, H-1´); 6.77 - 6.85 (m, 2×4H, H-*m*-C$_6$H$_4$OMe-DMTr); 7.03 – 7.09 (m, 2×2H, H-*o*-Ph); 7.14 – 7.20 (m, 2×1H, H-*p*-Ph); 7.21 – 7.33 (m, 2×5H, H-*m*-Ph, H-*m,p*-C$_6$H$_5$-DMTr); 7.33 – 7.40 (m, 2×4H, H-*o*-C$_6$H$_4$OMe-DMTr); 7.45 – 7.50 (m, 2×2H, H-*o*-C$_6$H$_5$-DMTr); 8.10 and 8.12 (2×s, 2×1H, H-6).

^{13}C NMR (125.7 MHz, CD_3CN): 20.97 and 21.04 (2×d, $J_{C,P}$ = 7.3 Hz, OCH$_2$**C**H$_2$CN); 24.84, 24.88 and 24.90 (3×d, $J_{C,P}$ = 7.5 Hz, (**C**H$_3$)$_2$CH); 40.94 (d, $J_{C,P}$ = 4.6 Hz, CH$_2$-2´); 41.11 (d, $J_{C,P}$ = 3.5 Hz, CH$_2$-2´); 44.01 and 44.02 (2×d, $J_{C,P}$ = 12.3 Hz, (CH$_3$)$_2$**C**H); 55.82 and 55.83 (CH$_3$O-DMTr); 59.47 and 59.48 (2×d, $J_{C,P}$ = 19.3 Hz, O**C**H$_2$CH$_2$CN); 63.85 and 64.09 (CH$_2$-5´); 73.85 (d, $J_{C,P}$ = 16.3 Hz, CH-3´); 74.31 (d, $J_{C,P}$ = 17.2 Hz, CH-3´); 82.12 and 82.14 (**C**≡C-Ph); 86.45 (d, $J_{C,P}$ = 5.9 Hz, CH-4´); 86.57 and 86.68 (CH-1´); 86.69 (d, $J_{C,P}$ = 5.9 Hz, CH-4´); 87.63 and 87.64 (C-DMTr); 93.25 and 93.27 (C≡**C**-Ph); 100.14 and 100.23 (C-5); 114.14 (CH-*m*-C$_6$H$_4$OMe-DMTr); 119.40 and 119.53 (OCH$_2$CH$_2$**C**N); 123.48 (CH-*i*-Ph); 127.89 (CH-*p*-C$_6$H$_5$-DMTr); 128.94 and 128.99 (CH-

o,m-C_6H_5-DMTr); 129.26 and 129.28 (CH-*m*-Ph); 129.41 and 129.43 (CH-*p*-Ph); 130.93, 130.95, 131.02 and 131.05 (CH-*o*-C_6H_4OMe-DMTr); 132.26 (CH-*o*-Ph); 136.56, 136.62, 136.74 and 136.80 (C-*i*-C_6H_4OMe-DMTr); 143.69 and 143.73 (CH-6); 145.78 (C-*i*-C_6H_5-DMTr); 150.32 (C-2); 159.65 and 159.68 (C-*p*-C_6H_4OMe-DMTr); 162.32 and 162.35 (C-4).

$^{31}P\{^1H\}$ NMR (202.4 MHz, CD_3CN): 150.47 and 150.42.

HR ESI-MS calculated m/z: 831.35173 $[M+H]^+$, 853.33367 $[M+Na]^+$, found m/z: 831.35193 $[M+H]^+$, 853.33395 $[M+Na]^+$.

2.1.7 Synthesis of Modified 2′-Deoxycytidine

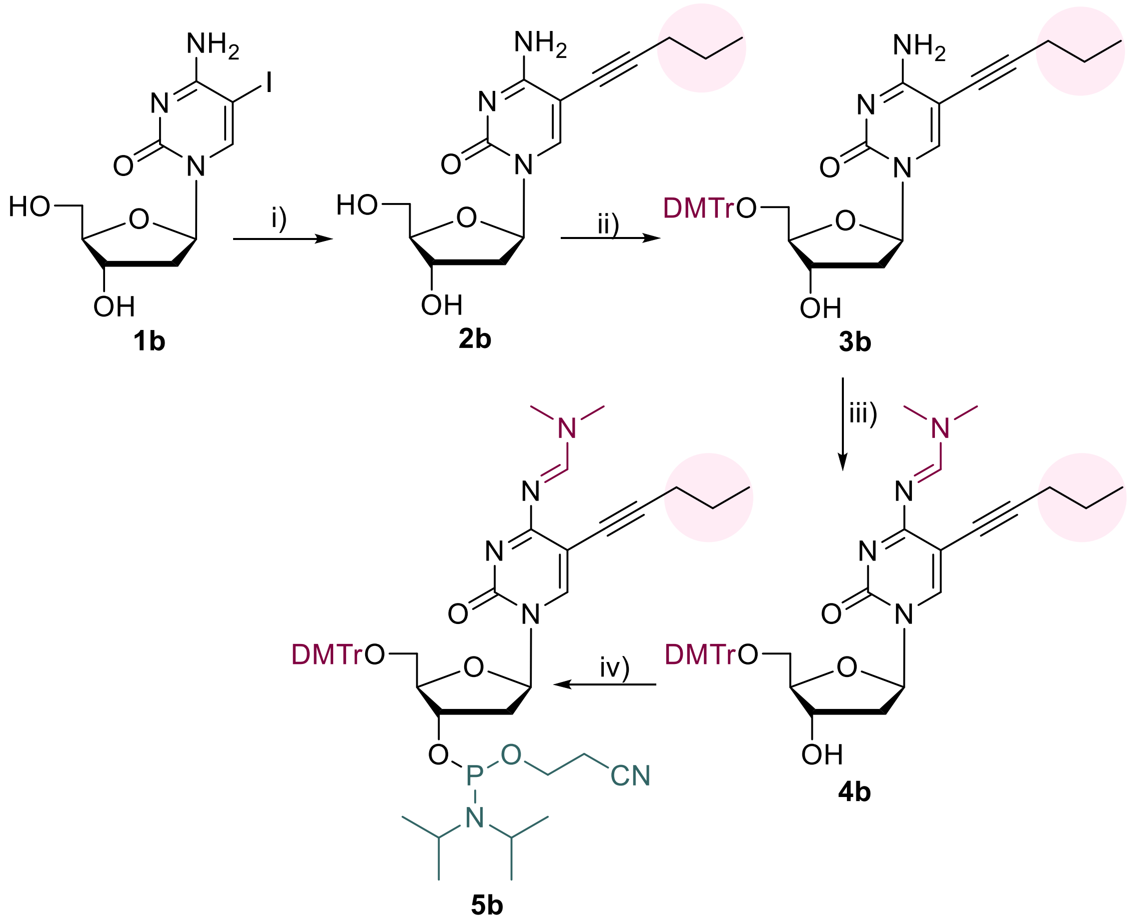

Scheme S3 Reagents and conditions: i) pent-1-yne (10 equiv.), $Pd(OAc)_2$ (0.1 equiv.), CuI (0.1 equiv.), TPPTS (0.1 equiv.), TEA (6 equiv.), MeCN/H_2O (1:1), RT, Ar, overnight; ii) DMTrCl (1.2 equiv.), DMAP (0.1 equiv.), pyridine (dry), RT, overnight; iii) DMF-DMA (14 equiv.), DMF (dry), 40°C, Ar, 4 h; iv) 2-cyanoethyl-*N,N*-diisopropylchlorophosphoramidite (1.2 equiv.), DIPEA (2.5 equiv.), DCM (dry), 0 °C to RT, 1.5 h;

5'-*O*-[Bis(4-methoxyphenyl)phenylmethyl]-5-(pent-1-yn-1-yl)-2′-deocytidine (3b)

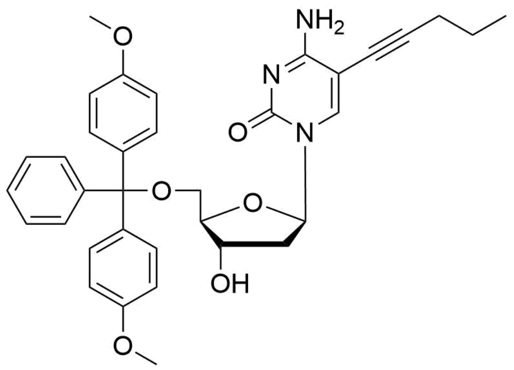

General method A was used to obtain compound **3b**. After dissolving compound **2b** (170 mg, 0.58 mmol) in dry pyridine (2 mL), DMAP (7 mg, 0.06 mmol) and solution of DMTrCl (236 mg, 0.70 mmol) in 1 mL of dry pyridine were added. Afforded product **3b** (289 mg, 84%) was in a form of pinkish foam.

^{1}H NMR (500.0 MHz, DMSO-d_6): 0.81 (t, 3H, J_{vic} = 7.4, C**H$_3$**CH$_2$CH$_2$); 1.34 (qt, 2H, J_{vic} = 7.4, 7.1, CH$_3$C**H$_2$**CH$_2$); 2.09 (ddd, 1H, J_{gem} = 13.1, $J_{2'b,1'}$ = 7.4, $J_{2'b,3'}$ = 6.3, H-2′b); 2.15, 2.19 (2 × dt, 2 × 1H, J$_{gem}$ = 16.8, J_{vic} = 7.1, CH$_3$CH$_2$C**H$_2$**); 2.23 (ddd, 1H, J_{gem} = 13.4, $J_{2'a,1'}$ = 6.1, $J_{2'a,3'}$ = 3.3, H-2′a); 3.12 (dd, 1H, J_{gem} = 10.5, $J_{5'b,4'}$ = 2.8, H-5′b); 3.20 (dd, 1H, J_{gem} = 10.5, $J_{5'a,4'}$ = 5.0, H-5′a); 3.730, 3.733 (2 × s, 2 × 3H, CH$_3$O-DMTr); 3.93 (ddd, 1H, $J_{4',5'}$ = 5.0, 2.8, $J_{4',3'}$ = 3.3, H-4′); 4.26 (ddt, 1H, $J_{3',2'}$ = 6.3, 3.3, $J_{3',OH}$ = 4.3, $J_{3',4'}$ = 3.3, H-3′); 5.30 (d, 1H, $J_{OH,3'}$ = 4.3, OH-3′); 6.13 (dd, 1H, $J_{1',2'}$ = 7.4, 6.1, H-1′); 6.72 (bs, 1H, NH$_a$**H$_b$**); 6.86 – 6.90 (m, 4H, H-*m*-C$_6$H$_4$OMe-DMTr); 7.22 (m, 1H, H-*p*-C$_6$H$_5$-DMTr); 7.27 – 7.33 (m, 6H, H-*o*-C$_6$H$_4$OMe-DMTr, H-*m*-C$_6$H$_5$-DMTr); 7.30 – 7.43 (m, 2H, H-*o*-C$_6$H$_5$-DMTr); 7.70 (bs, 1H, N**H$_a$**H$_b$); 7.88 (s, 1H, H-6).

^{13}C NMR (125.7 MHz, DMSO-d_6): 13.61 (**C**H$_3$CH$_2$CH$_2$); 21.16 (CH$_3$CH$_2$**C**H$_2$); 21.51 (CH$_3$**C**H$_2$CH$_2$); 41.10 (CH$_2$-2′); 55.21 (CH$_3$O-DMTr); 63.81 (CH$_2$-5′); 70.77 (CH-3′); 71.77 (**C**≡C-Pr); 85.51 (CH-1′); 85.94 (CH-4′); 86.03 (C-DMTr); 90.95 (C-5); 95.71 (C≡**C**-Pr); 113.37, 113.39 (CH-*m*-C$_6$H$_4$OMe-DMTr); 126.83 (CH-*p*-C$_6$H$_5$-DMTr); 127.82 (CH-*o*-C$_6$H$_5$-DMTr); 128.07 (CH-*m*-C$_6$H$_5$-DMTr); 129.80, 129.89 (CH-*o*-C$_6$H$_4$OMe-DMTr); 135.57, 135.82 (C-*i*-C$_6$H$_4$OMe-DMTr); 142.89 (CH-6); 144.88 (C-*i*-C$_6$H$_5$-DMTr); 153.60 (C-2); 158.24, 158.26 (C-*p*-C$_6$H$_4$OMe-DMTr); 164.59 (C-4).

HR ESI-MS calculated m/z: 596.27551 [M+H]$^+$, 618.25746 [M+Na]$^+$, found m/z: 596.27539 [M+H]$^+$, 618.25710 [M+Na]$^+$.

***N*[4]-Dimethylformimidine-5'-*O*-[bis(4-methoxyphenyl)phenylmethyl]-5-(pent-1-yn-1-yl)-2′-deocytidine (4b)**

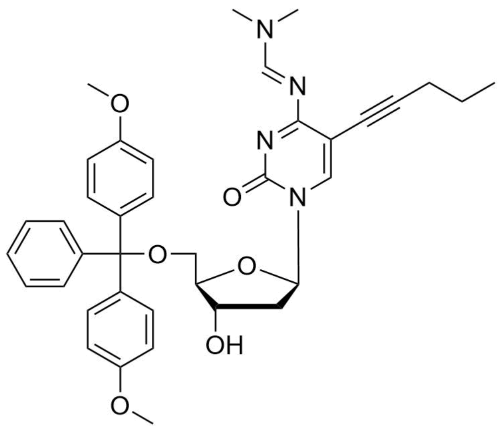

To synthesize compound **4b**, General method B was used. Upon dissolution of compound **3b** (1.0 g, 1.7 mmol) in dry DMF (40 mL), DMF-DMA (3.1 mL, 23.5 mmol) was added. Compound **4b** (1.0 g, 95%) was obtained as a yellowish foam.

^{1}H NMR (500.2 MHz, DMSO-*d*$_6$): 0.89 (t, 3H, $J_{CH3,CH2}$ = 7.3 Hz, C**H$_3$**CH$_2$CH$_2$); 1.34 (m, 2H, CH$_3$C**H$_2$**CH$_2$); 2.05 – 2.20 (m, 3H, H-2´a, CH$_3$CH$_2$C**H$_2$**); 2.28 (ddd, 1H, J_{gem} = 13.3 Hz, $J_{2´b,1´}$ = 6.1 Hz, $J_{2´b,3´}$ = 3.3 Hz, H-2´b); 3.08 (d, 3H, $J_{CH3,CH}$ = 0.8 Hz, (CH$_3$)$_2$N); 3.14 (dd, 1H, J_{gem} = 10.6 Hz, $J_{5´a,4'}$ = 2.8 Hz, H-5´a); 3.19 (s, 3H, (CH$_3$)$_2$N); 3.22 (dd, 1H, J_{gem} = 10.6 Hz, $J_{5´b,4´}$ = 5.2 Hz, H-5´b); 3.730, 3.732 (2 × s, 2 × 3H, CH$_3$O-DMTr); 3.96 (dt, 1H, $J_{4´,5´b}$ = 5.2 Hz, $J_{4´,5´a}$ = $J_{4´,3´}$ = 3.1 Hz, H-4´); 4.27 (bdq, 1H, $J_{3´,2´a}$ = 6.9 Hz, $J_{3´,2´b}$ = $J_{3´,4´}$ = $J_{3´,OH}$ = 3.5 Hz, H-3´); 5.32 (d, 1H, $J_{OH,3´}$ = 4.4 Hz, OH-3´); 6.14 (dd, 1H, $J_{1´,2´a}$ = 7.2 Hz, $J_{1´,2´b}$ = 6.1 Hz, H-1´); 6.85 – 6.91 (m, 4H, H-*m*-C$_6$H$_4$OMe-DMTr); 7.22 (m, 1H, H-*p*-C$_6$H$_5$-DMTr); 7.28 – 7.33 (m, 6H, H-*o*-C$_6$H$_4$OMe-DMTr, H-*m*-C$_6$H$_5$-DMTr); 7.39 – 7.44 (m, 2H, H-*o*-C$_6$H$_5$-DMTr); 7.99 (s, 1H, H-6); 8.58 (m, 1H, NCH=N).

^{13}C NMR (125.8 MHz, DMSO-*d*$_6$): 13.40 (**C**H$_3$CH$_2$CH$_2$); 21.00 (CH$_3$CH$_2$**C**H$_2$); 21.72 (CH$_3$**C**H$_2$CH$_2$); 34.84 and 41.00 ((CH$_3$)$_2$N); 41.18 (CH$_2$-2´); 55.19 (CH$_3$O-DMTr); 63.79 (CH$_2$-5´); 70.70 (CH-3´); 74.50 (**C**≡C-Pr); 85.91 (CH-1´); 86.03 (C-DMTr); 86.08 (CH-4´); 92.50 (C≡**C**-Pr); 98.84 (C-5); 113.37 and 113.39 (CH-*m*-C$_6$H$_4$OMe-DMTr); 126.82 (CH-*p*-C$_6$H$_5$-DMTr); 127.81 (CH-*o*-C$_6$H$_5$-DMTr); 128.05 (CH-*m*-C$_6$H$_5$-DMTr); 129.81 and 129.88 (CH-*o*-C$_6$H$_4$OMe-DMTr); 135.58 and 135.82 (C-*i*-C$_6$H$_4$OMe-DMTr); 143.86 (CH-6); 144.89 (C-*i*-C$_6$H$_5$-DMTr); 153.60 (C-2); 158.03 (NCH=N); 158.25 (C-*p*-C$_6$H$_4$OMe-DMTr); 170.27 (C-4).

HR ESI-MS calculated m/z: 651.31771 [M+H]$^+$, 673.29966 [M+Na]$^+$, found m/z: 651.31747 [M+H]$^+$, 673.29952 [M+Na]$^+$.

N^4-Dimethylformamidine-5'-*O*-[bis(4-methoxyphenyl)phenylmethyl]-5-(pent-1-yn-1-yl)-2′-deocytidine-3′-(2-cyanoethyl *N,N*-diisopropylphosphoramidite) (5b)

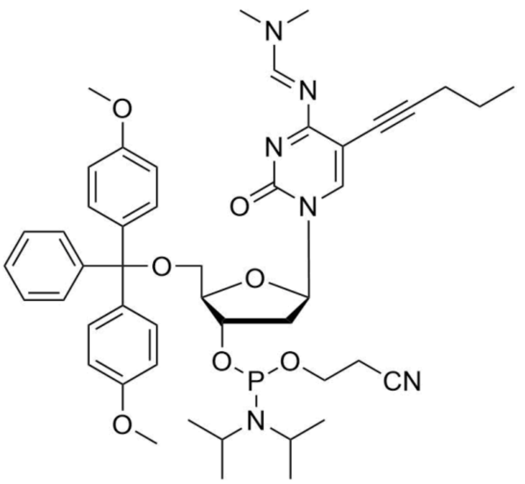

Compound **5b** was prepared using General method C. Protected nucleoside **4b** (1 g, 1.57 mmol) was dissolved in dry DCM (8 mL) and DIPEA (683 μL, 3.92 mmol) was added followed by 2-cyanoethyl-*N*,*N*-diisopropylchlorophosphoramidite (420 μL, 1.88 mmol). The completion of the reaction was confirmed by TLC analysis (EtOAc/MeOH 10:1, 1.5 h). Extraction and purification by flash chromatography (EtOAc 100% to EtOAc/MeOH 10:1 with 0.5% TEA) under argon atmosphere provided final compound **5b** as two diastereomers in a ratio 3.4:1 (867 mg, 65%) in a form of a white foam.

^{1}H NMR (500.0 MHz, CD_3CN): 0.90 (t, 3H, $J_{CH3,CH2}$ = 7.4 Hz, $C\mathbf{H_3}CH_2CH_2$-*B*); 0.91 (t, 3H, $J_{CH3,CH2}$ = 7.4 Hz, $C\mathbf{H_3}CH_2CH_2$-*A*); 1.06 and 1.15 (2×d, 2×3H, $J_{CH3,CH}$ = 6.8 Hz, $(C\mathbf{H_3})_2CH$-*A*); 1.15 and 1.17 (2×d, 2×3H, $J_{CH3,CH}$ = 6.8 Hz, $(C\mathbf{H_3})_2CH$-*B*); 1.31 - 1.42 (m, 2×2H, $CH_3C\mathbf{H_2}CH_2$-*A*,*B*); 2.10 (td, 2H, $J_{CH2,CH2}$ = 7.1 Hz, $J_{CH2,P}$ = 1.8 Hz, $CH_3CH_2C\mathbf{H_2}$-*B*); 2.12 (td, 2H, $J_{CH2,CH2}$ = 7.1 Hz, $J_{CH2,P}$ = 1.6 Hz, $CH_3CH_2C\mathbf{H_2}$-*A*); 2.27 (dt, 1H, J_{gem} = 13.8 Hz, $J_{2'a,1'}$ = $J_{2'a,3'}$ = 6.5 Hz, H-2´a-*A*); 2.29 (dt, 1H, J_{gem} = 13.6 Hz, $J_{2'a,1'}$ = $J_{2'a,3'}$ = 6.5 Hz, H-2´a-*B*); 2.51 (ddd, 1H, J_{gem} = 13.6 Hz, $J_{2'b,1'}$ = 6.3 Hz, $J_{2'b,3'}$ = 3.8 Hz, H-2´b-*B*); 2.53 (t, 2H, $J_{CH2,CH2}$ = 6.0 Hz, $OCH_2C\mathbf{H_2}CN$-*B*); 2.57 (ddd, 1H, J_{gem} = 13.8 Hz, $J_{2'b,1'}$ = 6.2 Hz, $J_{2'b,3'}$ = 3.4 Hz, H-2´b-*A*); 2.64 (t, 2H, $J_{CH2,CH2}$ = 6.0 Hz, $OCH_2C\mathbf{H_2}CN$-*A*); 3.11 and 3.15 (2×s, 4×3H, $(CH_3)_2N$-*A*,*B*); 3.24 – 3.34 (m, 2×2H, H-5´-*A*,*B*); 3.50 – 3.64 (m, 4×1H, $(CH_3)_2C\mathbf{H}$-*A*,*B*); 3.63 – 3.84 (m, 2×2H, $OC\mathbf{H_2}CH_2CN$-*A*,*B*); 3.753 and 3.756 (2×s, 2×3H, CH_3O-DMTr-*A*); 3.757 and 3.760 (2×s, 2×3H, CH_3O-DMTr-*B*); 4.13 (bq, 1H, $J_{4',5'b}$ = $J_{4',5'a}$ = $J_{4',3'}$ = 3.5 Hz, H-4´-*A*); 4.17 (bq, 1H, $J_{4',5'b}$ = $J_{4',5'a}$ = $J_{4',3'}$ = 3.5 Hz, H-4´-*B*); 4.56 (ddt, 1H, $J_{3',P}$ = 10.0 Hz, $J_{3',2'a}$ = 6.4 Hz, $J_{3',2'b}$ = $J_{3',4'}$ = 3.4 Hz, H-3´-*A*); 4.60 (ddt, 1H, $J_{3',P}$ = 10.4 Hz, $J_{3',2'a}$ = 6.5 Hz, $J_{3',2'b}$ = $J_{3',4'}$ = 3.5 Hz, H-3´-*B*); 6.14 (t, 1H, $J_{1',2'a}$ = $J_{1',2'b}$ = 6.4 Hz, H-1´-*B*); 6.17 (t, 1H, $J_{1',2'a}$ = $J_{1',2'b}$ = 6.6 Hz, H-1´-*A*); 6.82 – 6.89 (m, 2×4H, H-*m*-C_6H_4OMe-DMTr-*A*,*B*); 7.19 – 7.25 (m, 2×1H, H-*p*-C_6H_5-DMTr-*A*,*B*); 7.27 – 7.33 (m, 2×2H, H-*m*-C_6H_5-DMTr-*A*,*B*); 7.34 – 7.40 (m, 2×4H, H-*o*-C_6H_4OMe-DMTr-

A,*B*); 7.45 – 7.51 (m, 2×2H, H-*o*-C_6H_5-DMTr-*A*,*B*); 8.03 (s, 1H, H-6-*A*); 8.06 (s, 1H, H-6-*B*); 8.63 (s, 2×1H, NCH=N-*A*,*B*).

^{13}C NMR (125.7 MHz, CD_3CN): 13.83 (**C**$H_3CH_2CH_2$-*A*,*B*); 20.95 (d, $J_{C,P}$ = 7.1 Hz, OCH_2**C**H_2CN-*A*); 21.01 (d, $J_{C,P}$ = 7.1 Hz, OCH_2**C**H_2CN-*B*); 21.99 (CH_3CH_2**C**H_2-*A*,*B*); 22.87 (CH_3**C**H_2CH_2-*B*); 22.88 (CH_3**C**H_2CH_2-*A*); 24.80 – 24.92 (m, (**C**$H_3)_2CH$); 35.41 ($(CH_3)_2N$-*A*,*B*); 41.43 (d, $J_{C,P}$ = 4.5 Hz, CH_2-2´-*B*); 41.62 (d, $J_{C,P}$ = 3.6 Hz, CH_2-2´-*A*); 41.71 ($(CH_3)_2N$-*A*,*B*); 43.98 (d, $J_{C,P}$ = 12.3 Hz, $(CH_3)_2$**C**H-*B*); 43.99 (d, $J_{C,P}$ = 12.3 Hz, $(CH_3)_2$**C**H-*A*); 55.88 (CH_3O-DMTr-*A*); 55.83 (CH_3O-DMTr-*B*); 59.46 (d, $J_{C,P}$ = 19.1 Hz, O**C**H_2CH_2CN-*A*,*B*); 63.82 (CH_2-5´-*B*); 64.19 (CH_2-5´-*A*); 73.80 (d, $J_{C,P}$ = 16.0 Hz, CH-3´-*B*); 74.49 (d, $J_{C,P}$ = 17.1 Hz, CH-3´-*A*); 75.22 (**C**≡CCH_2); 86.29 (d, $J_{C,P}$ = 5.8 Hz, CH-4´-*A*); 86.48 (d, $J_{C,P}$ = 4.2 Hz, CH-4´-*B*); 87.39 (CH-1´-*A*); 87.41 (CH-1´-*B*); 87.49 (C-DMTr-*A*,*B*); 93.60 (C≡**C**CH_2-*B*); 93.62 (C≡**C**CH_2-*A*); 100.09 (CH-5-*B*); 100.15 (C-5-*A*); 114.09 (CH-*m*-C_6H_4OMe-DMTr-*A*); 114.11 (CH-*m*-C_6H_4OMe-DMTr-*B*); 119.37 (OCH_2CH_2**C**N-*B*); 119.50 (OCH_2CH_2**C**N-*A*); 127.81 (CH-*p*-C_6H_5-DMTr-*A*,*B*); 128.92 and 128.99 (CH-*o*,*m*-C_6H_5-DMTr-*A*,*B*); 130.98 and 131.07 (CH-*o*-C_6H_4OMe-DMTr-*A*); 131.02 and 131.10 (CH-*o*-C_6H_4OMe-DMTr-*B*); 136.63 and 136.82 (C-*i*-C_6H_4OMe-DMTr-*A*); 136.70 and 136.86 (C-*i*-C_6H_4OMe-DMTr-*B*); 144.89 (CH-6-*A*,*B*); 145.90 (C-*i*-C_6H_5-DMTr-*A*); 145.92 (C-*i*-C_6H_5-DMTr-*B*); 155.14 (C-2-*A*); 155.15 (C-2-*B*); 158.92 (NCH=N-*B*); 158.93 (NCH=N-*A*); 159.65 and 159.67 (C-*p*-C_6H_4OMe-DMTr-*A*,*B*); 171.73 (C-4-*A*); 171.75 (C-4-*B*).

^{31}P{^{1}H}i NMR (202.4 MHz, CD_3CN): 150.27 and 150.22.

HR ESI-MS calculated m/z: 851.42556 $[M+H]^+$, 873.40751 $[M+Na]^+$, found m/z: 851.42542 $[M+H]^+$, 873.40737 $[M+Na]^+$.

2.1.8 Synthesis of Modified 2′-Deoxyadenosine

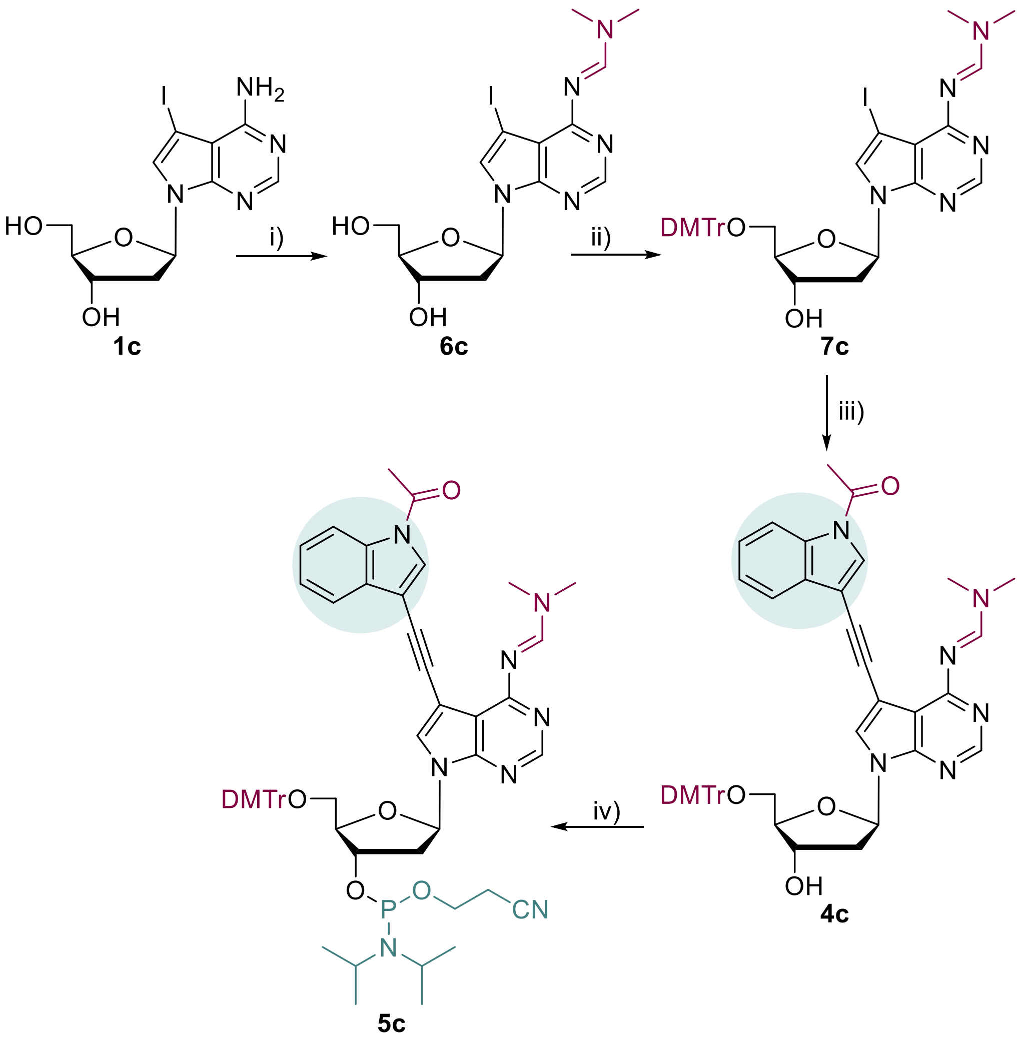

Scheme S4 Reagents and conditions: i) *N,N*-dimethylformamide dimethylacetal (14 equiv.), DMF (dry), 40°C, Ar, 4 h; ii) DMTrCl (1.3 equiv.), DMAP (0.1 equiv.), pyridine (dry), RT, overnight; iii) 1-acetyl-3-[(trimethylsilyl)ethynyl]indole (1.3 equiv.), $(PPh_3)_2PdCl_2$ (0.2 equiv.), CuI (0.4 equiv.), TEA (5 equiv.), TEA·3HF (5 equiv.), DMF, RT, Ar, overnight; iv) 2-cyanoethyl-*N,N*-diisopropylchlorophosphoramidite (1.5 equiv.), DIPEA (2.5 equiv.), DCM (dry), 0 °C to RT, 1.5 h;

N^6-Dimethylformamidine-5'-*O*-[bis(4-methoxyphenyl)phenylmethyl]-7-(1-acetyl-1*H*-indol-3-yl)ethynyl)-2′-deoxy-7-deazaadenosine (4c)

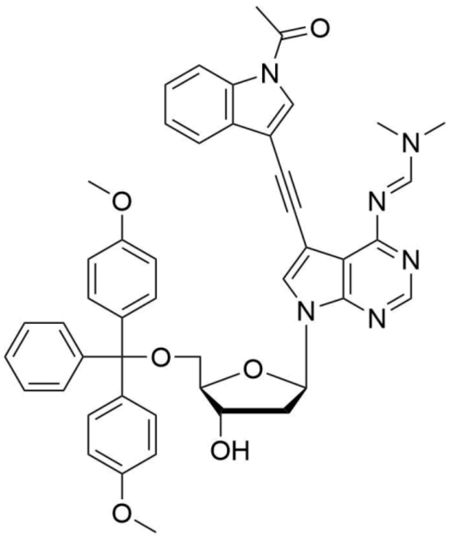

Compound **7c** (100 mg, 0.14 mmol) was dissolved in dry DMF under argon atmosphere along with $(PPh_3)_2PdCl_2$ (19 mg, 0.03 mmol) and CuI (10 mg, 0.05 mmol). Then, 1-(3-((trimethylsilyl)ethynyl)-1*H*-indol-1-yl)ethan-1-one (45 mg, 0.18 mmol) and TEA·3HF (111 μL, 0.68 mmol) were added, followed by injection of TEA (95 μL, 0.68 mmol). After overnight stirring, the solvent was evaporated and subjected to HPFC (0–10% MeOH in $CHCl_3$ with 0.5% TEA). Product **4c** was isolated as a brownish powder (86 mg, 80%).

^{1}H NMR (500.0 MHz, DMSO-d_6): 2.33 (bddd, 1H, J_{gem} = 13.1 Hz, $J_{2'a,1'}$ = 6.0 Hz, $J_{2'a,3'}$ = 4.3 Hz, H-2´a); 2.68 (s, 3H, CH_3CO); 2.68 (m, 1H, H-2´b); 3.09 and 3.14 (2×s, 2×3H, $(CH_3)_2N$); 3.13 – 3.22 (m, 2H, H-5´); 3.65 and 3.66 (2×s, 2×3H, CH_3O-DMTr); 3.97 (m, 1H, H-4´); 4.86 (m, 1H, H-3´); 5.37 (d, 1H, $J_{OH,3'}$ = 4.5 Hz, OH-3´); 6.61 (t, 1H, $J_{1',2'b}$ = $J_{1',2'a}$ = 6.2 Hz, H-1´); 6.77 – 6.89 (m, 4H, H-*m*-C_6H_4OMe-DMTr); 7.16 (m, 1H, H-*p*-C_6H_5-DMTr); 7.20 – 7.32 (m, 6H, H-*m*-C_6H_5-DMTr, H-*o*-C_6H_4OMe-DMTr); 7.31 – 7.46 (m, 3H, H-*o*-C_6H_5-DMTr, H-5-indolyl); 7.42 (m, 1H, H-6-indolyl); 7.63 (d, 1H, $J_{4,5}$ = 7.7 Hz, H-4-indolyl); 7.89 (s, 1H, H-6); 7.08 (s, 1H, H-2-indolyl); 8.36 (d, 1H, $J_{7,6}$ = 8.2 Hz, H-7-indolyl), 8.85 (s, 1H, NCH=N). *Signal of proton H-2 was not detected.*

^{13}C NMR (125.7 MHz, DMSO-d_6): 23.77 (**C**H_3CO); 34.55 ($(CH_3)_2N$); 39.54 (CH_2-2´); 40.34 ($(CH_3)_2N$); 54.90 and 54.92 (CH_3O-DMTr); 64.10 (CH_2-5´); 70.64 (CH-3´); 81.13 (5-C≡**C**); 82.81 (CH-1´); 85.45 (C-DMTr); 85.49 (CH-4´); 87.81 (5-**C**≡C); 97.18 (C-5); 104.13 (C-3-indolyl); 110.45 (C-4a); 113.10 (CH-*m*-C_6H_4OMe-DMTr); 116.10 (CH-7-indolyl); 119.61 (CH-4-indolyl); 123.80 (CH-5-indolyl); 125.66 (CH-6-indolyl); 126.59 (CH-*p*-C_6H_5-DMTr); 127.71 and 127.78 (CH-*o,m*-C_6H_5-DMTr); 128.10 (CH-6); 129.11 (CH-2-indolyl); 126.63 and 129.70 (CH-*o*-C_6H_4OMe-DMTr); 129.98 (C-3a-indolyl); 134.30 (C-7a-indolyl); 135.55 and 135.58 (C-*i*-C_6H_4OMe-DMTr); 144.85 (C-*i*-C_6H_5-DMTr); 151.11 (C-7a); 156.52 (NCH=N); 157.96 and 157.99 (C-*p*-C_6H_4OMe-DMTr); 169.37 (CH_3**C**O). *Signals of carbon C-2 and C-4 were not detected.*

HR ESI-MS calculated m/z: 789.33951 $[M+H]^+$, 811.32145 $[M+Na]^+$, found m/z: 789.33967 $[M+H]^+$, 811.32156 $[M+Na]^+$.

***N*[6]-Dimethylformamidine-5'-*O*-[bis(4-methoxyphenyl)phenylmethyl]-7-(1-acetyl-1*H*-indol-3-yl)ethynyl)-2′-deoxy-7-deazaadenosine-3′-(2-cyanoethyl *N,N*-diisopropylphosphoramidite) (5c)**

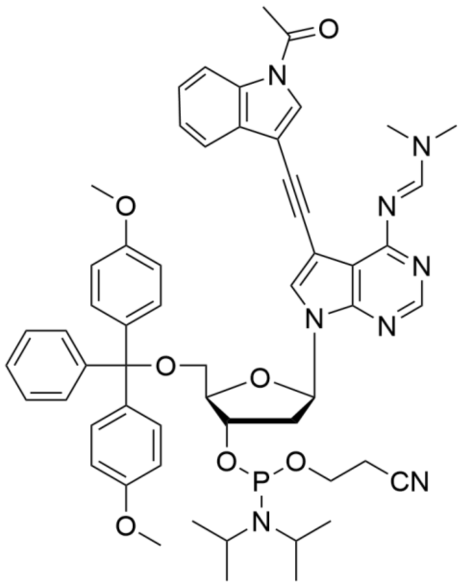

Protected nucleoside **4c** (480 mg, 0.61 mmol) was converted to the corresponding phosphoramidite using General method C. Freshly distilled DIPEA (265 μL, 1.52 mmol) and subsequently 2-cyanoethyl-*N*,*N*-diisopropylchlorophosphoramidite (204 μL, 0.91 mmol) were injected. The completion of the reaction was confirmed by TLC analysis (cyclohexane/EtOAc 1:3, 1 h). Purification provided final compound **5c** as a single diastereomer (595 mg, 81%) in a form of a white foam.

^{1}H NMR (500.0 MHz, CD_3CN): 1.18 (d, 2×6H, $J_{CH3,CH}$ = 6.8 Hz, (C**H₃**)₂CHN); 2.53 (t, 2H, $J_{CH2,CH2}$ = 6.0 Hz, $OCH_2C\mathbf{H_2}CN$); 2.56 (ddd, 1H, J_{gem} = 13.6 Hz, $J_{2'a,1'}$ = 6.7 Hz, $J_{2'a,3'}$ = 4.8 Hz, H-2´a); 2.62 (s, 3H, CH_3CO); 2.83 (dt, 1H, J_{gem} = 13.6 Hz, $J_{2'b,1'}$ = $J_{2'b,3'}$ = 6.4 Hz, H-2´b); 3.08 and 3.12 (2×s, 2×3H, $(CH_3)_2N$); 3.25 (dd, 1H, J_{gem} = 10.6 Hz, $J_{5'a,4'}$ = 4.7 Hz, H-5´a); 3.34 (dd, 1H, J_{gem} = 10.6 Hz, $J_{5'b,4'}$ = 3.0 Hz, H-5´b); 3.57 – 3.67 (m, 2H, $(CH_3)_2C\mathbf{H}N$); 3.66 – 3.71 (m, 2H, $OC\mathbf{H_2}CH_2CN$), 3.667 and 3.672 (2×s, 2×3H, CH_3O-DMTr); 4.17 (m, 1H, H-4´); 4.86 (ddt, 1H, $J_{3',P}$ = 10.9 Hz, $J_{3',2'b}$ = 6.7 Hz, $J_{3',2'a}$ = $J_{3',4'}$ = 4.5 Hz, H-3´); 6.63 (t, 1H, $J_{1',2'b}$ = $J_{1',2'a}$ = 6.4 Hz, H-1´); 6.77 – 6.84 (m, 4H, H-*m*-C_6H_4OMe-DMTr); 7.17 (m, 1H, H-*p*-C_6H_5-DMTr); 7.23 – 7.35 (m, 7H, H-*m*-C_6H_5-DMTr, H-*o*-C_6H_4OMe-DMTr, H-5-indolyl); 7.38 – 7.45 (m, 3H, H-*o*-C_6H_5-DMTr, H-6-indolyl); 7.62 (bdt, 1H, $J_{4,5}$ = 7.7 Hz, $J_{4,6}$ = $J_{4,7}$ = 1.0 Hz, H-4-indolyl); 7.72 (s, 1H, H-6); 7.77 (s, 1H, H-2-indolyl); 8.40 (m, 1H, H-7-indolyl), 8.79 (s, 1H, NCH=N).

^{13}C NMR (125.7 MHz, CD_3CN): 20.98 (d, $J_{C,P}$ = 7.1 Hz, $OCH_2\mathbf{C}H_2CN$); 24.34 (**C**H_3CO); 24.90 and 24.91 (2×d, $J_{C,P}$ = 7.3 Hz, $(\mathbf{C}H_3)_2CHN$); 35.28 ($(CH_3)_2N$); 39.90 (d, $J_{C,P}$ = 4.3 Hz, CH_2-2´); 41.20 ($(CH_3)_2N$); 44.02 ($(CH_3)_2\mathbf{C}HN$); 55.81 (CH_3O-DMTr); 59.49 (d, $J_{C,P}$ = 19.0 Hz, $O\mathbf{C}H_2CH_2CN$); 64.31 (CH_2-5´); 73.82 (d, $J_{C,P}$ = 16.4 Hz, CH-3´); 82.06 (5-C≡**C**); 84.46 (CH-1´); 85.95 (d, $J_{C,P}$ = 4.1 Hz, CH-4´); 87.10 (C-DMTr); 88.69 (5-**C**≡C); 98.37 (C-5); 105.77 (C-3-indolyl); 111.59 (C-4a); 114.01 (CH-*m*-C_6H_4OMe-DMTr); 117.28 (CH-7-indolyl); 119.43 ($OCH_2CH_2\mathbf{C}N$); 120.83 (CH-4-

indolyl); 124.88 (CH-5-indolyl); 126.73 (CH-6-indolyl); 127.80 (CH-*p*-C_6H_5-DMTr); 128.82 (CH-*m*-C_6H_5-DMTr); 128.92 (CH-6); 129.11 (CH-*o*-C_6H_5-DMTr); 129.53 (CH-2-indolyl); 130.98 and 131.03 (CH-*o*-C_6H_4OMe-DMTr); 131.41 (C-3a-indolyl); 135.96 (C-7a-indolyl); 136.90 and 136.92 (C-*i*-C_6H_4OMe-DMTr); 145.97 (C-*i*-C_6H_5-DMTr); 152.59 (C-7a); 153.52 (CH-2); 157.63 (NCH=N); 159.58 and 158.60 (C-*p*-C_6H_4OMe-DMTr); 162.52 (C-4); 170.15 (CH_3**CO**).

^{31}P NMR (202.4 MHz, CD_3CN): 149.32 (s, 1P).

HR ESI-MS calculated m/z: 989.44736 $[M+H]^+$, found m/z: 989.44782 $[M+H]^+$.

2.1.9 Synthesis of Modified 2′-Deoxyguanosine

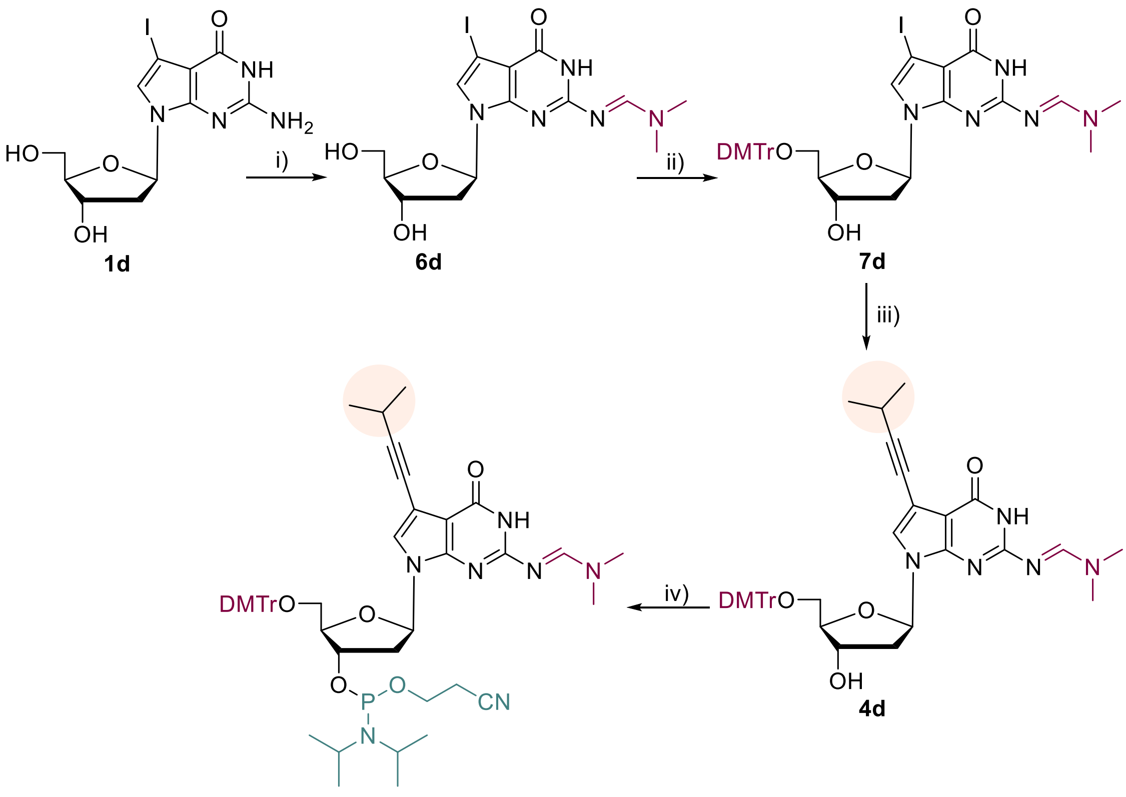

Scheme S5 Reagents and conditions: i) *N*,*N*-dimethylformamide dimethylacetal (14 equiv.), DMF (dry), 40°C, Ar, 4 h; ii) DMTrCl (1.3 equiv.), DMAP (0.1 equiv.), pyridine (dry), RT, overnight; iii) 3-methylbut-1-yne (25 equiv.), $(PPh_3)_2PdCl_2$ (0.2 equiv.), CuI (0.4 equiv.), TEA (5 equiv.), , DMF, RT, Ar, overnight; iv) 2-cyanoethyl-*N*,*N*-diisopropylchlorophosphoramidite (1.5 equiv.), DIPEA (2.5 equiv.), DCM (dry), 0 °C to RT, 1.5 h;

N^2-Dimethylformamidine-7-iodo-2′-deoxy-7-deazaguanosine (6d)

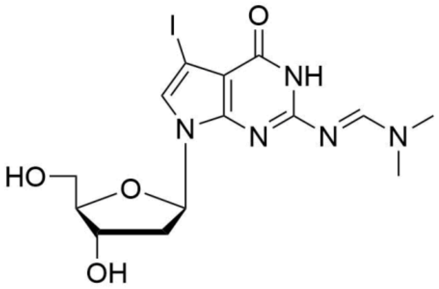

General method B was used to synthesize compound **6d**. Compound **1d** (1 g, 2.6 mmol) was dissolved in dry DMF (15 mL) and DMF-DMA (4.8 mL, 35.7 mmol) was added. Afforded product **6d** (694 mg, 67%) was in a form of a white powder.

^{1}H NMR (500 MHz, DMSO-d$_6$): 2.11 (ddd, 1H, J_{gem} = 13.1 Hz, $J_{2´a,1´}$ = 6.0 Hz, $J_{2´a,3´}$ = 2.7 Hz, H-2´a); 2.38 (ddd, 1H, J_{gem} = 13.1 Hz, $J_{2´b,1´}$ = 8.2 Hz, $J_{2´b,3´}$ = 5.8 Hz, H-2´b); 3.01 and 3.15 (2×s, 2×3H, $(CH_3)_2N$); 3.49 (bdt, 1H, J_{gem} = 11.9 Hz, $J_{5´a,4´}$ = $J_{5´a,OH}$ = 4.9 Hz, H-5´a); 3.53 (bdt, 1H, J_{gem} = 11.9 Hz, $J_{5´b,4´}$ = $J_{5´b,OH}$ = 5.1 Hz, H-5´b); 3.77 (td, 1H, $J_{4´,5´a}$ = $J_{4´,5´b}$ = 4.5 Hz, $J_{4´,3´}$ = 2.5 Hz, H-4´); 4.30 (bdq, 1H, $J_{3´,2´b}$ = 5.8 Hz, $J_{3´,2´a}$ = $J_{3´,OH}$ = $J_{3´,4´}$ = 2.4 Hz, H-3´); 4.90 (t, 1H, $J_{OH,5´}$ = 5.4 Hz, OH-5´); 5.25 (d, 1H, $J_{OH,3´}$ = 3.7 Hz, OH-3´); 6.40 (dd, 1H, $J_{1´,2´b}$ = 8.2 Hz, $J_{1,2´a}$ = 6.0 Hz, H-1´); 7.25 (s, 1H, H-6); 8.54 (s, 1H, NCHN); 11.09 (s, 1H, NH).

^{13}C NMR (125.7 MHz, DMSO-d$_6$): 34.64 ($(CH_3)_2N$); 39.54 (CH_2-2´); 40.59 ($(CH_3)_2N$); 55.27 (C-5); 61.92 (CH_2-5´); 70.99 (CH-3´); 82.28 (CH-1´); 87.27 (CH-4´); 102.77 (C-4a); 123.25 (CH-6); 149.39 (C-7a); 156.19 (CH-2); 157.53 (NCHN); 158.95 (C-4).

HR ESI-MS calculated m/z: 448.04762 $[M+H]^+$, 470.02957 $[M+Na]^+$, found m/z: 448.04890 $[M+H]^+$, 470.02988 $[M+Na]^+$.

N^2-Dimethylformamidine-5'-*O*-[bis(4-methoxyphenyl)phenylmethyl]-7-iodo-2′-deoxy-7-deazaguanosine (7d)

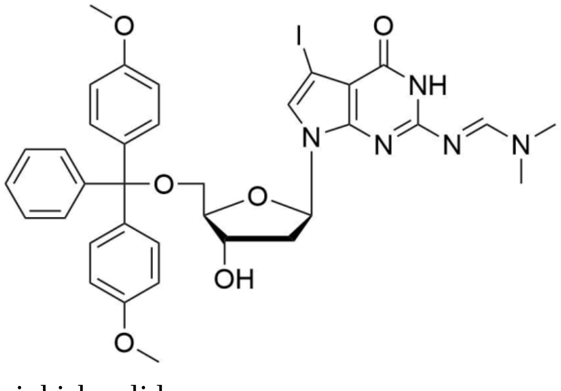

General method A was used to prepare compound **7d**. Upon dissolution of compound **6d** (654 mg, 1.46 mmol) in dry pyridine (6 mL), DMAP (16 mg, 0.15 mmol) and DMTrCl (644 mg, 1.90 mmol) were added. The purification afforded compound **7d** (936 mg, 85%) as a pinkish solid.

^{1}H NMR (500.0 MHz, DMSO-d$_6$): 2.19 (ddd, 1H, J_{gem} = 13.4 Hz, $J_{2´a,1´}$ = 6.3 Hz, $J_{2´a,3´}$ = 3.5 Hz, H-2´a); 2.45 (bdt, 1H, J_{gem} = 13.4 Hz, $J_{2´b,1´}$ = $J_{2´b,3´}$ = 6.7 Hz, H-2´a); 3.02 (s, 3H, $(CH_3)_2N$); 3.08 (dd, 1H, J_{gem} = 10.2 Hz, $J_{5´a,4'}$ = 3.4 Hz, H-5´a); 3.14 (s, 3H, $(CH_3)_2N$); 3.18 (dd, 1H, J_{gem} = 10.2 Hz, $J_{5´b,4´}$ = 6.0 Hz, H-5´b); 3.736 and 3.739 (s, 2×3H, CH_3O-

DMTr); 3.89 (dt, 1H, $J_{4´,5´b}$ = 5.9 Hz, $J_{4´,5´a}$ = $J_{4´,3´}$ = 3.4 Hz, H-4´); 4.32 (bdq, 1H, $J_{3´,2´b}$ = 7.3 Hz, $J_{3´,4´}$ = $J_{3´,2´a}$ = $J_{3´,OH}$ = 3.7 Hz, H-3´); 5.32 (d, 1H, $J_{OH,3´}$ = 4.2 Hz, OH-3´); 6.44 (bt, 1H, $J_{1´,2´b}$ = $J_{1´,2´a}$ = 6.9 Hz, H-1´); 6.84 – 6.90 (m, 4H, H-*m*-C_6H_4OMe-DMTr); 7.11 (s, 1H, H-6); 7.18 – 7.32 (m, 7H, H-*m*,*p*-C_6H_5-DMTr, H-*o*-C_6H_4OMe-DMTr); 7.35 – 7.40 (m, 2H, H-*o*-C_6H_5-DMTr); 8.56 (s, 1H, NCHN); 11.12 (bs, 1H, NH).

^{13}C NMR (125.7 MHz, DMSO-d$_6$): 34.65 (($CH_3)_2$N); 39.89 (CH_2-2´); 40.60 (($CH_3)_2$N); 55.07 and 55.09 (CH_3O-DMTr); 55.54 (C-5); 64.29 (CH_2-5´); 70.80 (CH-3´); 82.12 (CH-1´); 85.39 (CH-4´); 85.55 (C-DMTr); 102.86 (C-4a); 113.19 and 113.20 (CH-*m*-C_6H_4OMe-DMTr); 122.94 (CH-6); 126.66 (CH-*p*-C_6H_5-DMTr); 127.71 (CH-*o*-C_6H_5-DMTr); 127.87 (CH-*m*-C_6H_5-DMTr); 129.71 (CH-*o*-C_6H_4OMe-DMTr); 135.49 and 135.62 (C-*i*-C_6H_4OMe-DMTr); 144.90 (C-*i*-C_6H_5-DMTr); 149.42 (C-7a); 156.25 (C-2); 157.55 (NCH=N); 158.05 and 158.06 (C-*p*-C_6H_4OMe-DMTr); 158.97 (C-4).

HR ESI-MS calculated m/z: 750.17830 $[M+H]^+$, 772.16025 $[M+Na]^+$, found m/z: 750.17866 $[M+H]^+$, 772.16055 $[M+Na]^+$.

***N*^{2}-Dimethylformamidine-5'-*O*-[bis(4-methoxyphenyl)phenylmethyl]-7-(3-methylbut-1-yn-1-yl)-2′-deoxy-7-deazaguanosine (4d)**

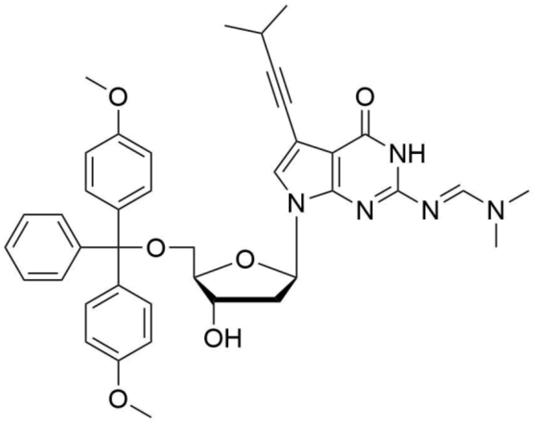

To the solution of compound **7d** (2.5 g, 3.35 mmol) under argon atmosphere in dry DMF (15 mL) in the pressure tube, $(PPh_3)_2PdCl_2$ (470 mg, 0.67 mmol) and CuI (255 mg, 1.34 mmol) were added. Then, 3-methylbut-1yne (8.6 mL, 83.71 mmol) and TEA (2.3 mL, 16.74 mmol) were injected. After overnight stirring, the mixture was evaporated and subjected to HPFC (0–10% MeOH in $CHCl_3$ with 0.5% TEA). Product **4d** was isolated as a yellowish powder (1.9 g, 83%).

^{1}H NMR (500.0 MHz, DMSO-d$_6$): 1.16 and 1.17 (2×d, 2×3H, $J_{CH3,CH}$ = 6.8 Hz, (C**H$_3$**)$_2$CH); 2.18 (ddd, 1H, J_{gem} = 13.4 Hz, $J_{2´a,1´}$ = 6.4 Hz, $J_{2´a,3´}$ = 3.7 Hz, H-2´a); 2.45 (dt, 1H, J_{gem} = 13.4 Hz, $J_{2´b,1´}$ = $J_{2´b,3´}$ = 6.8 Hz, H-2´a); 2.74 (sept, 1H, $J_{CH,CH3}$ = 6.8 Hz, ($CH_3)_2$C**H**); 3.02 (s, 3H, ($CH_3)_2$N); 3.05 (dd, 1H, J_{gem} = 10.1 Hz, $J_{5´a,4'}$ = 3.3 Hz, H-5´a); 3.14 (s, 3H, ($CH_3)_2$N); 3.20 (dd, 1H, J_{gem} = 10.1 Hz, $J_{5´b,4´}$ = 6.4 Hz, H-5´b); 3.727 and 3.732 (s, 2×3H, CH_3O-DMTr); 3.89 (dt, 1H, $J_{4´,5´b}$ = 6.3 Hz, $J_{4´,5´a}$ = $J_{4´,3´}$ = 3.4 Hz, H-4´); 4.30 (bdq, 1H, $J_{3´,2´b}$ = 6.2 Hz, $J_{3´,4´}$ = $J_{3´,2´a}$ = $J_{3´,OH}$ = 3.8 Hz, H-3´); 5.31 (d, 1H, $J_{OH,3´}$ =

4.2 Hz, OH-3´); 6.45 (bt, 1H, $J_{1´,2´b}$ = $J_{1´,2´a}$ = 6.9 Hz, H-1´); 6.81 – 6.89 (m, 4H, H-*m*-C_6H_4OMe-DMTr); 7.15 (s, 1H, H-6); 7.17 – 7.33 (m, 7H, H-*m,p*-C_6H_5-DMTr, H-*o*-C_6H_4OMe-DMTr); 7.34 – 7.42 (m, 2H, H-*o*-C_6H_5-DMTr); 8.56 (s, 1H, NCHN); 11.05 (bs, 1H, NH).

^{13}C NMR (125.7 MHz, DMSO-d$_6$): 20.68 ((CH_3)$_2$**C**HC≡C); 22.98 ((**C**H_3)$_2$CHC≡C); 34.62 ((CH_3)$_2$N); 39.71 (CH_2-2´); 40.57 ((CH_3)$_2$N); 55.01 (CH_3O-DMTr); 64.35 (CH_2-5´); 70.79 (CH-3´); 73.58 ((CH_3)$_2$CHC≡**C**); 82.02 (CH-1´); 85.41 (CH-4´); 85.48 (C-DMTr); 95.29 ((CH_3)$_2$CH**C**≡C); 99.70 (C-5); 102.50 (C-4a); 113.14 and 113.16 (CH-*m*-C_6H_4OMe-DMTr); 122.18 (CH-6); 126.59 (CH-*p*-C_6H_5-DMTr); 127.73 (CH-*o*-C_6H_5-DMTr); 127.83 (CH-*m*-C_6H_5-DMTr); 129.69 and 129.77 (CH-*o*-C_6H_4OMe-DMTr); 135.45 and 135.73 (C-*i*-C_6H_4OMe-DMTr); 144.93 (C-*i*-C_6H_5-DMTr); 149.03 (C-7a); 156.55 (C-2); 157.52 (NCH=N); 158.03 and 158.05 (C-*p*-C_6H_4OMe-DMTr); 158.77 (C-4).

HR ESI-MS calculated m/z: 690.32861 $[M+H]^+$, 712.31056 $[M+Na]^+$, found m/z: 690.32882 $[M+H]^+$, 712.31069 $[M+Na]^+$.

N^2-Dimethylformamidine-5'-*O*-[bis(4-methoxyphenyl)phenylmethyl]-7-(3-methylbut-1-yn-1-yl)-2′-deoxy-7-deazaguanosine-3′-(2-cyanoethyl *N,N*-diisopropylphosphoramidite) (5d)

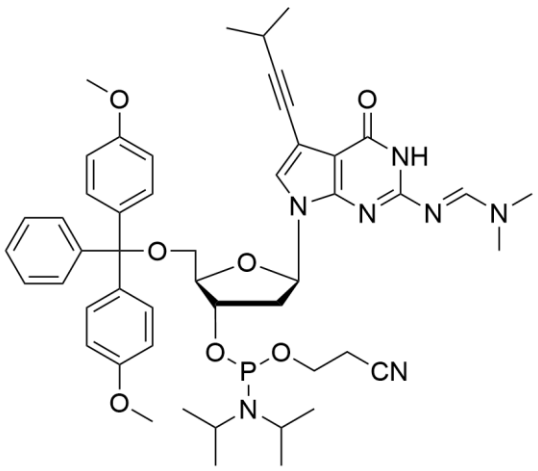

General method C was used to synthesize compound **5d**. Compound **4d** (810 mg, 1.17 mmol) was dissolved in dry DCM (10 mL), subsequently DIPEA (511 μL, 2.94 mmol) and 2-cyanoethyl-*N,N*-diisopropylchlorophosphoramidite (392 μL, 1.76 mmol) were added. After purification final compound **5d** (816 mg, 70%) was obtained as a mixture of two diastereomers in ratio 1:1.2 in a form of white foam.

^{1}H NMR (500.0 MHz, CD_3CN): 0.99 and 1.06 – 1.12 (12×d, 12×3H, $J_{CH3,CH}$ = 6.8 Hz, (C**H$_3$**)$_2$CHC≡C, (C**H$_3$**)$_2$CHN); 2.41 (ddd, 1H, J_{gem} = 13.6 Hz, $J_{2´a,1´}$ = 6.4 Hz, $J_{2´a,3´}$ = 3.7 Hz, H-2´a); 2.48 (ddd, 1H, J_{gem} = 13.9 Hz, $J_{2´a,1´}$ = 6.5 Hz, $J_{2´a,3´}$ = 3.9 Hz, H-2´a); 2.53 (t, 2×2H, $J_{CH2,CH2}$ = 6.0 Hz, OCH_2C**H$_2$**CN); 2.55 - 2.62 (m, 2×1H, H-2´b); 2.64 (t, 2×2H, $J_{CH2,CH2}$ = 6.0 Hz, OCH_2C**H$_2$**CN); 2.69 – 2.79 (m, 2×1H, (CH_3)$_2$C**H**C≡C); 3.038, 3.040,

3.106 and 3.112 (4×s, 4×3H, $(CH_3)_2N$); 3.17 -3.30 (m, 2×2H, H-5´); 3.752, 3.758 and 3.763 (4×s, 4×3H, CH_3O-DMTr); 3.53 -3.86 (m, 2×4H, $(CH_3)_2C\mathbf{H}N$, $OC\mathbf{H_2}CH_2CN$), 4.08 and 4.12 (2×m, 2×1H, H-4´); 4.64 – 4.72 (m, 2×1H, H-3´); 6.486 and 6.492 (2×t, 2×1H, $J_{1´,2´b}$ = $J_{1´,2´a}$ = 6.8 Hz, H-1´); 6.80 – 6.87 (m, 2×4H, H-*m*-C_6H_4OMe-DMTr); 7.021 and 7.023 (2×s, 2×1H, H-6); 7.19 – 7.25 (m, 2×1H, H-*p*-C_6H_5-DMTr); 7.26 – 7.34 (m, 2×6H, H-*m*-C_6H_5-DMTr, H-*o*-C_6H_4OMe-DMTr); 7.40 – 7.47 (m, 2×2H, H-*o*-C_6H_5-DMTr); 8.60 and 8.61 (2×s, 2×1H, NCH=N); 9.09 (bs, 2×1H, NH).

^{13}C NMR (125.7 MHz, CD_3CN): 20.98 and 21.05 (2×d, $J_{C,P}$ = 7.1 Hz, $OCH_2\mathbf{C}H_2CN$); 22.03 ($(CH_3)_2\mathbf{C}HC{\equiv}C$); 23.44 and 23.45 ($(\mathbf{C}H_3)_2CHC{\equiv}C$); 24.81 – 24.95 (m, $(\mathbf{C}H_3)_2CHN$); 35.17 ($(CH_3)_2N$); 40.10 and 40.20 (2×d, $J_{C,P}$ = 4.4 and 3.3 Hz, CH_2-2´); 41.53 ($(CH_3)_2N$); 44.02 (d, $J_{C,P}$ = 12.3 Hz, $(CH_3)_2\mathbf{C}HN$); 55.88 and 55.90 (CH_3O-DMTr); 59.42 and 59.57 (2×d, $J_{C,P}$ = 19.0 Hz, $O\mathbf{C}H_2CH_2CN$); 64.67 and 64.79 (CH_2-5´); 74.04 ($(CH_3)_2CHC{\equiv}\mathbf{C}$); 74.39 and 74.69 (2×d, $J_{C,P}$ = 17.1 and 17.7 Hz, CH-3´); 83.83 and 83.90 (CH-1´); 85.60 and 85.84 (2×d, $J_{C,P}$ = 5.9 and 4.6 Hz, CH-4´); 87.13 and 87.16 (C-DMTr); 96.77 ($(CH_3)_2CH\mathbf{C}{\equiv}C$); 101.41 and 101.43 (C-5); 103.98 and 104.00 (C-4a); 114.05 (CH-*m*-C_6H_4OMe-DMTr); 119.43 and 119.57 ($OCH_2CH_2\mathbf{C}N$); 123.11 and 123.14 (CH-6); 127.79 and 127.82 (CH-*p*-C_6H_5-DMTr); 128.86 and 127.87 (CH-*m*-C_6H_5-DMTr); 128.98 and 129.04 (CH-*o*-C_6H_5-DMTr); 130.98, 131.01, 131.05 and 131.08 (CH-*o*-C_6H_4OMe-DMTr); 136.69, 136.75, 136.86 and 136.90 (C-*i*-C_6H_4OMe-DMTr); 146.07 and 146.08 (C-*i*-C_6H_5-DMTr); 150.52 and 152.56 (C-7a); 157.56 and 157.58 (CH-2); 158.75 and 158.80 (NCH=N); 159.646, 159.653, 159.662, 159.671, 159.680 and 158.685 (C-*p*-C_6H_4OMe-DMTr, C-4).

^{31}P NMR (202.4 MHz, CD_3CN): 149.31 and 159.39 (2×s, 2×1P).

HR ESI-MS calculated m/z: 890.43646 $[M+H]^+$, found m/z: 890.43598 $[M+H]^+$.

3 Synthesis of Oligonucleotides

3.1 Solid-phase Synthesis

Synthesis of partially and hyper-modified oligonucleotides **ON1*–ON12*** with the phosphoramidites **5a**, **5b**, **5c** and **5d** was performed in a 1 μmole scale. The trityl-off mode has been used to prevent the loss of the products in another purification round (which was shown crucial with the hyper-modified **ON11***, **ON12***, where the yield of the solid-phase synthesis was rather low) and also to avoid the risk of a strong attachment to the reverse phase and possible problems with the elution due to the increased hydrophobicity of the modified ONs. For partially modified **ON1*–ON10*** the standard solid-phase columns were used, for hyper-modified ONs* (**ON11***, **ON12***) the universal solid-phase columns were used. Each phosphoramidite was diluted to a 0.1 M solution and 0.3 M 5-(benzylthio)-1*H*-tetrazole (BTT) solution in MeCN was used as an activator. Iodine solution (0.02 M) in THF/pyridine/water (ratio 70:20:10) was used for the oxidation step. Standard cycle procedures provided by BioAutomation Corporation were applied for the unmodified as well as for the modified phosphoramidites. The coupling volume and duration for the natural phosphoramidites were 220 μL and 1 minute 30 seconds whereas for the modified phosphoramidite the coupling time was increased to 6 minutes to maximize the incorporation efficiency. Cleavage from the solid-phase was performed by 30% aqueous NH_3 for 2×45 minutes (2×1 mL). Following deprotection step was carried out by incubation of the oligonucleotide solutions at 65 °C for 6 hours.

3.2 Purification and Characterization

The purification of the oligonucleotides was performed using HPLC with a linear gradient of MeCN (0–100%) in 0.1 M triethylammonium bicarbonate (TEAB) buffer (pH 7.6). The final lyofilization from H_2O provided pure products. The approximate concentrations were measured by UV/VIS Spectrophotometer at 260 nm, mass and purity were then measured on UHPLC-MS. Most of the modified oligonucleotides (ONs*) are >90% pure, except **ON4*** showing 84% purity. The exact concentrations and yields were determined based on the phosphorus content measured by the elemental analysis of pure ONs. The sequences, calculated and measured masses, purities and isolated yields of the chemically-synthesized oligonucleotides are shown in **Table S1**.

Table S1 List of the chemically synthesized modified oligonucleotides **ON1*–ON12***, their sequences, calculated and measured masses [Da], their purities [%] and isolated yields [%].

**Code**	**Sequence (5′⟶3′)**	**Mass calc. [Da]**	**Mass found [Da]**	**Purity [%]**	Yield[a] [%]
**ON1***	ATCTCAG**A***GAACTGC	4699.17	4699.03	100	65
**ON2***	ATCTAG**A*A*A***GACTGC	4999.61	4999.09	100	3 (11)[a]
**ON3***	ATCTCAG**G***AAGCTGC	4642.12	4642.71	100	64
**ON4***	ATGTCA**G*G*G***AGCTGC	4828.45	4827.80	84	13
**ON5***	ATGTC**G*A*G*A***AGCTGC	5023.67	5023.07	95	6 (10)[a]
**ON6***	GCTCCGT**C***GATTGAA	4634.10	4633.91	98	51
**ON7***	GCTCC**C*C*C***GATTGAA	4711.31	4710.97	100	42
**ON8***	GCTCCGT**U***GATTGAA	4669.19	4668.71	98	34
**ON9***	GCTCCG**U*U*U***ATTGAA	4816.28	4815.83	97	8 (12)[a]
**ON10***	GCTCG**C*U*C*U***ATTGAA	4847.39	4846.76	100	11 (17)[a]
**ON11***	**G*U*A*G*A*U*G*C*A*C*U*C*G*U*C***	5851.75	5851.11	90	6 (17)[a]
**ON12***	**G*A*C*G*A*G*U*G*C*A*U*C*U*A*C***	5912.83	5911.88	98	5 (20)[a]

[a] HPLC yields given in parenthesis in case of lower isolated yield

3.3 Annealing

To anneal the **ON1*–ON12*** with the complementary strands **ON1–ON12** (shown in **Table S2**) a solution of complementary single stranded ONs (50 μL, 5 μM each) with the annealing buffer 2X (20 mM Tris, pH 7.5–8.0, 100 mM NaCl, 2 mM EDTA) were heated on a thermo-block to 95 °C for 5 min and then let cool down at RT. The formed **DNA1*–DNA10*** (**Table S3**) were visualised on an agarose gel (3%) stained with GelRed and analyzed in 0.5X TBE buffer. The resulting gel can be seen on **Figure S1**.

Table S2 List of sequences **ON1–ON12** complementary to **ON1*–ON12***.

Code	Sequence (5′⟶3′)
**ON1**	GCAGTTCTCTGAGAT
**ON2**	GCAGTCTTTCTAGAT
**ON3**	GCAGCTTCCTGAGAT
**ON4**	GCAGCTCCCTGACAT
**ON5**	GCAGCTTCTCGACAT
**ON6**	TTCAATCGACGGAGC
**ON7**	TTCAATCGGGGGAGC
**ON8**	TTCAATCAACGGAGC
**ON9**	TTCAATAAACGGAGC
**ON10**	TTCAATAGAGCGAGC
**ON11**	GACGAGTGCATCTAC
**ON12**	GTAGATGCACTCGTC

Table S3 List of annealed double-stranded non modified (DNA) and modified oligonucleotides (DNA*).

Code	DNA[a]	DNA*[b]
**DNA1**	5′-ATCTCAGAGAACTGC-3′<br>3′-TAGAGTCTCTTGACG-5′	5′-ATCTCAGA*GAACTGC-3′<br>3′-TAGAGTC T CTTGACG-5′
**DNA2**	5′-ATCTAGAAAGACTGC-3′<br>3′-TAGATCTTTCTGACG-5′	5′-ATCTAGA*A*A*GACTGC-3′<br>3′-TAGATC T T T CTGACG-5′
**DNA3**	5′-ATCTCAGGAAGCTGC-3′<br>3′-TAGAGTC C TTCGACG-5′	5′-ATCTCAGG*AAGCTGC-3′<br>3′-TAGAGTC C TTCGACG-5′
**DNA4**	5′-ATGTCAGGGAGCTGC-3′<br>TACAGTCCCTCGACG-5′	5′-ATGTCAG*G*G*AGCTGC-3′<br>TACAGT C C C TCGACG-5′
**DNA5**	5′-ATGTCGAGAAGCTGC-3′<br>3′-TACAGCTCTTCGACG-5′	5′-ATGTCG*A*G*A*AGCTGC-3′<br>3′-TACAG C T C T TCGACG-5′
**DNA6**	5′-GCTCCGTCGATTGAA-3′<br>3′-CGAGGCAGCTAACTT-5′	5′-GCTCCGTC*GATTGAA-3′<br>3′-CGAGGCA G CTAACTT-5′
**DNA7**	5′-GCTCCCCCGATTGAA-3′<br>3′-CGAGGGGGCTAACTT-5′	5′-GCTCCC*C*C*GATTGAA-3′<br>3′-CGAGG G G G CTAACTT-5′
**DNA8**	5′-GCTCCGTUGATTGAA-3′<br>3′-CGAGGCAACTAACTT-5′	5′-GCTCCGTU*GATTGAA-3′<br>3′-CGAGGCA A CTAACTT-5′
**DNA9**	5′-GCTCCGUUUATTGAA-3′<br>3′-CGAGGCAAATAACTT-5′	5′-GCTCCGU*U*U*ATTGAA-3′<br>3′-CGAGGC A A A TAACTT-5′
**DNA10**	5′-GCTCGCUCUATTGAA-3′<br>3′-CGAGCGAGATAACTT-5′	5′-GCTCGC*U*C*U*ATTGAA-3′<br>3′-CGAGC G A G A TAACTT-5′
**DNA11**[c]	5′-GUAGAUGCACUCGUC-3′<br>3′-CATCTACGTGAGCAG-5′	5′-G*U*A*G*A*U*G*C*A*C*U*C*G*U*C*-3′<br>3′-C A T C T A C G T G A G C A G-5′
**DNA12**	-[c]	5′-G*A*C*G*A*G*U*G*C*A*U*C*U*A*C*-3′<br>3′-C T G C T C A C G T A G A T G-5′
**DNA13**	-[c]	5′-G*U*A*G*A*U*G*C*A*C*U*C*G*U*C*-3′<br>3′-C*A*U*C*U*A*C*G*U*G*A*G*C*A*G*-5′

[a] **DNA1–DNA11** are the non-modified double-stranded DNA sequences; [b] **DNA1*–DNA12*** are dsDNA made by annealing of base-modified **ON1*–ON12*** with the corresponding non-modified complementary strands **ON1–ON12**, whilst the **DNA13*** was made by annealing of two modified strands **ON11*** and **ON12***; [c] non-modified **DNA11** corresponds to the same sequence as the non-modified **DNA12** and **DNA13** and also modified **DNA11*–DNA13***.

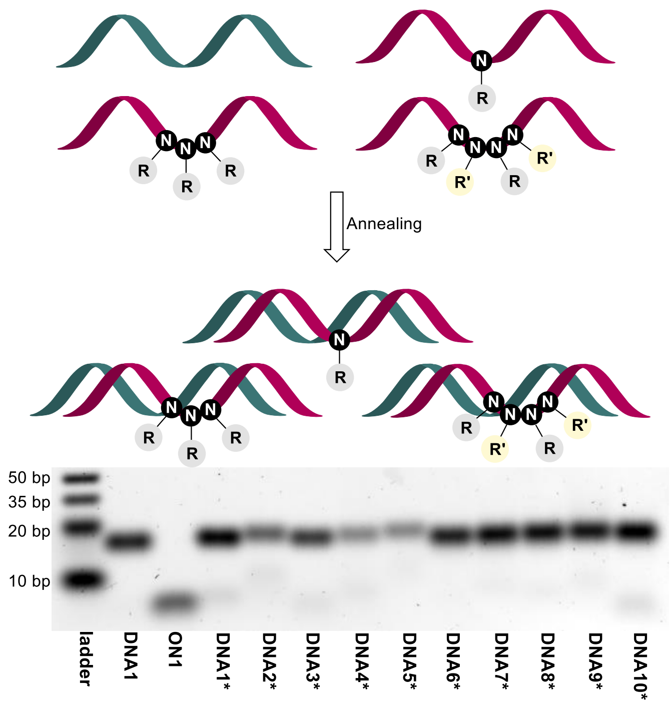

Figure S1 Annealing of the partially modified oligonucleotides (ONs*) with non-modified ONs visualised on agarose gel (3%) via GelRed. Ladder: ultra low-range DNA ladder; **DNA1**: non-modified ds-DNA with the sequence corresponding to modified **DNA1***; **ON1**: non-modified ss-ON with the sequence corresponding to partially modified **ON1***; **DNA1*–10*:** partially modified **ON1*–10*** annealed with their non-modified complementary strands.

3.4 Measurement of Melting Temperatures

The melting (T_m) and annealing temperatures (T_a), depicted in **Table S4**, were recorded in 1 mm rectangular quartz cell in UV-VIS Spectrophotometer, in a temperature range 25 °C–95 °C with the temperature increment 1 °C/min under 260 nm detection and were obtained from three cycles (6 ramps in total). T_m and T_a values (in °C) were calculated using the first negative derivative of the intensity over the temperature.

Table S4 Melting (T_m, [°C]) and annealing temperatures (T_a, [°C]) of non-modified double-stranded oligonucletides (DNA) and double-stranded modified oligonucleotides (DNA*) determined by UV-VIS spectroscopy. The difference of T_m and T_a between DNA* and DNA (ΔT_m and ΔT_a) and the difference per one modification (ΔT_m/modification).

<table>
<tr><th rowspan="2">#</th><th colspan="4">T_m [°C]</th><th colspan="4">T_a [°C]</th></tr>
<tr><th>DNA</th><th>mDNA</th><th>ΔT_m</th><th>ΔT_m / modification</th><th>DNA</th><th>mDNA</th><th>ΔT_a</th><th>ΔT_a / modification</th></tr>
<tr><td>1</td><td>51.8</td><td>49.6</td><td>-2.2</td><td>-2.2</td><td>46.3</td><td>43.8</td><td>-2.5</td><td>-2.5</td></tr>
<tr><td>2</td><td>47.6</td><td>44.4</td><td>-3.2</td><td>-1.1</td><td>43.2</td><td>40.3</td><td>-2.9</td><td>-1.0</td></tr>
<tr><td>3</td><td>55.9</td><td>54.7</td><td>-1.2</td><td>-1.2</td><td>50.9</td><td>50.2</td><td>-0.7</td><td>-0.7</td></tr>
<tr><td>4</td><td>59.8</td><td>57.9</td><td>-1.9</td><td>-0.6</td><td>55.7</td><td>54.2</td><td>-1.5</td><td>-0.5</td></tr>
<tr><td>5</td><td>58.6</td><td>55.7</td><td>-2.9</td><td>-0.7</td><td>54.0</td><td>50.9</td><td>-3.1</td><td>-0.8</td></tr>
<tr><td>6</td><td>57.3</td><td>57.5</td><td>+0.2</td><td>+0.2</td><td>52.0</td><td>52.4</td><td>+0.4</td><td>+0.4</td></tr>
<tr><td>7</td><td>57.4</td><td>64.6</td><td>+7.2</td><td>+2.4</td><td>53.3</td><td>59.9</td><td>+6.6</td><td>+2.2</td></tr>
<tr><td>8</td><td>53.3</td><td>52.2</td><td>-1.1</td><td>-1.1</td><td>48.9</td><td>47.1</td><td>-1.8</td><td>-1.8</td></tr>
<tr><td>9</td><td>48.5</td><td>47.5</td><td>-1.0</td><td>-0.3</td><td>44.5</td><td>42.8</td><td>-1.7</td><td>-0.6</td></tr>
<tr><td>10</td><td>52.3</td><td>58.6</td><td>+6.3</td><td>+1.6</td><td>47.8</td><td>52.4</td><td>+4.6</td><td>+1.2</td></tr>
<tr><td>11</td><td rowspan="3">54.9</td><td>62.0</td><td>+7.1</td><td>+0.5</td><td rowspan="3">52.5</td><td>56.3</td><td>+3.8</td><td>+0.3</td></tr>
<tr><td>12</td><td>62.3</td><td>+7.4</td><td>+0.5</td><td>56.4</td><td>+3.9</td><td>+0.3</td></tr>
<tr><td>13</td><td>61.5</td><td>+6.6</td><td>+0.2</td><td>56.2</td><td>+3.7</td><td>+0.1</td></tr>
</table>

4 Copies of Chromatograms, Spectra and Graphs

4.1 Absorbance Chromatograms of Oligonucleotides

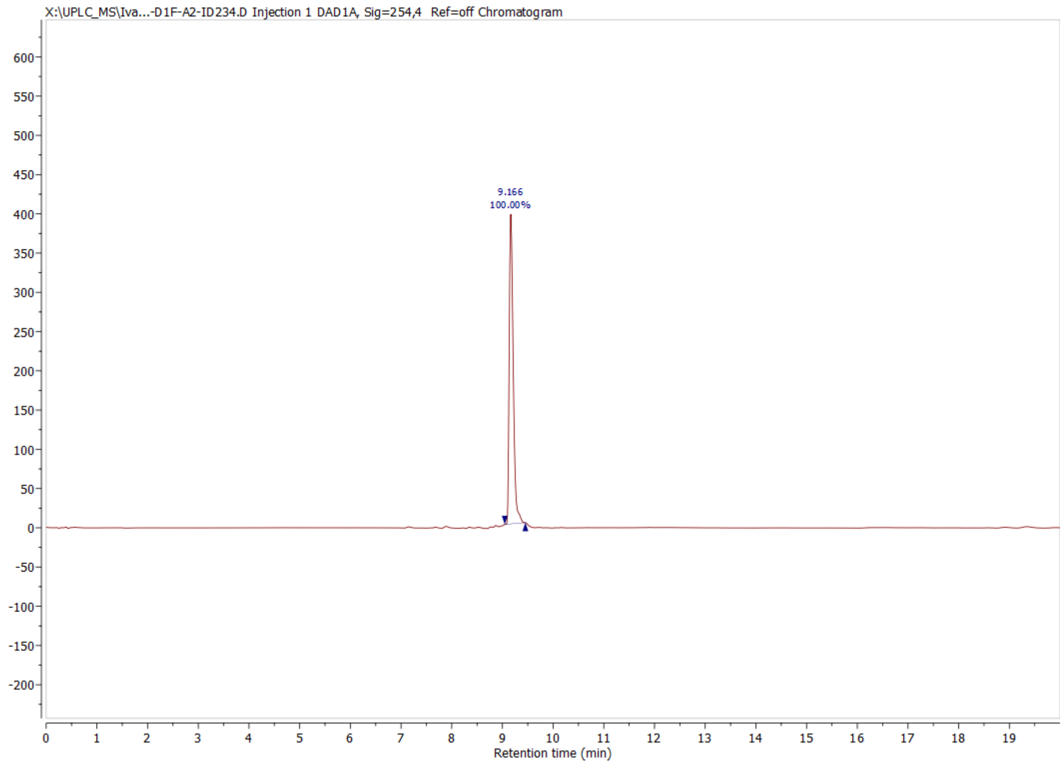

Figure S2 Absorbance chromatogram at 254 nm of ON1*.

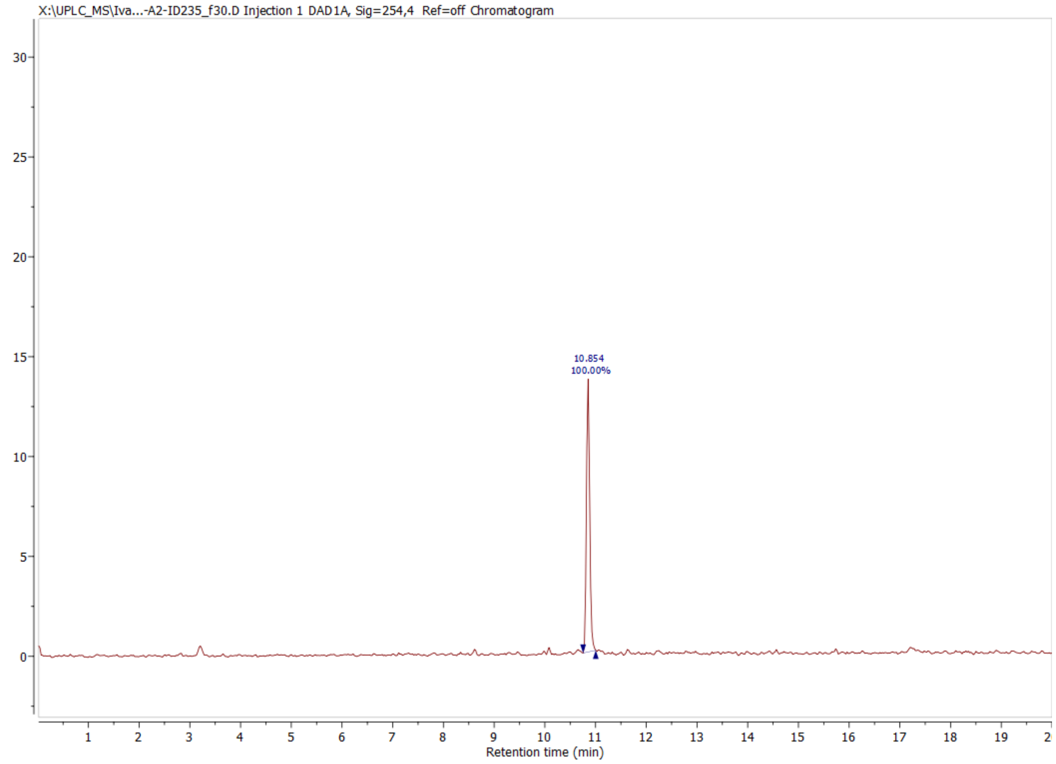

Figure S3 Absorbance chromatogram at 254 nm of ON2*.

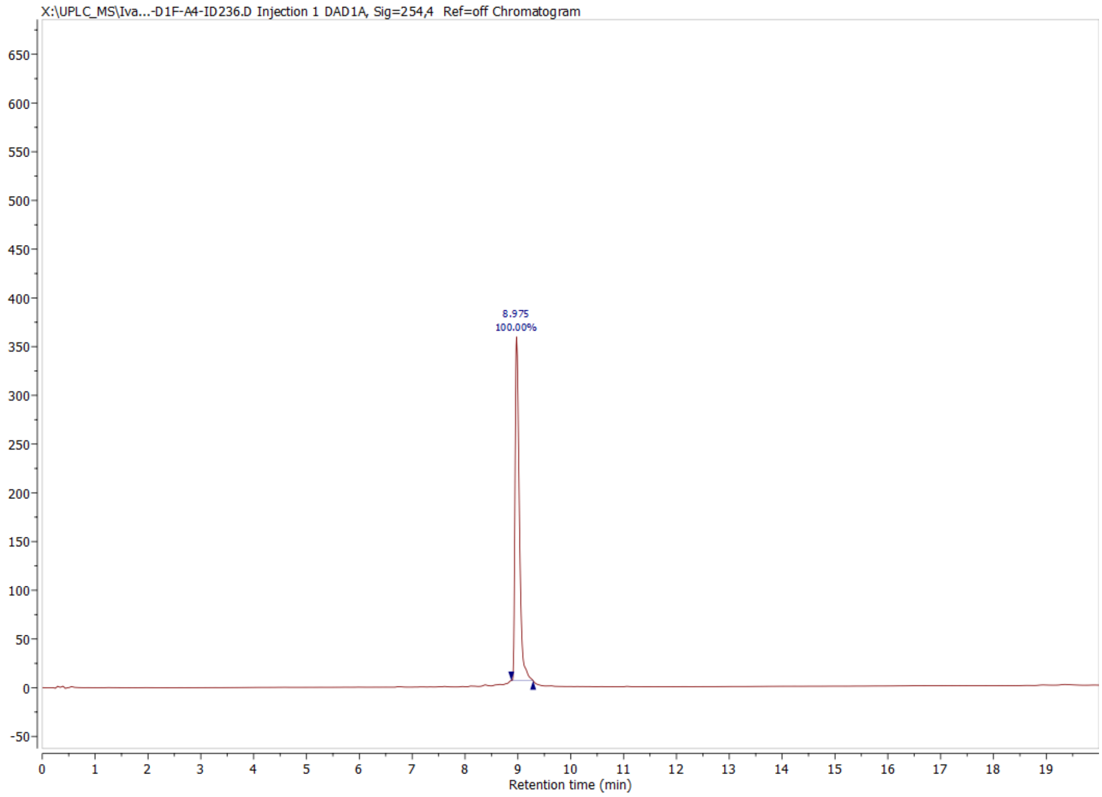

Figure S4 Absorbance chromatogram at 254 nm of ON3*.

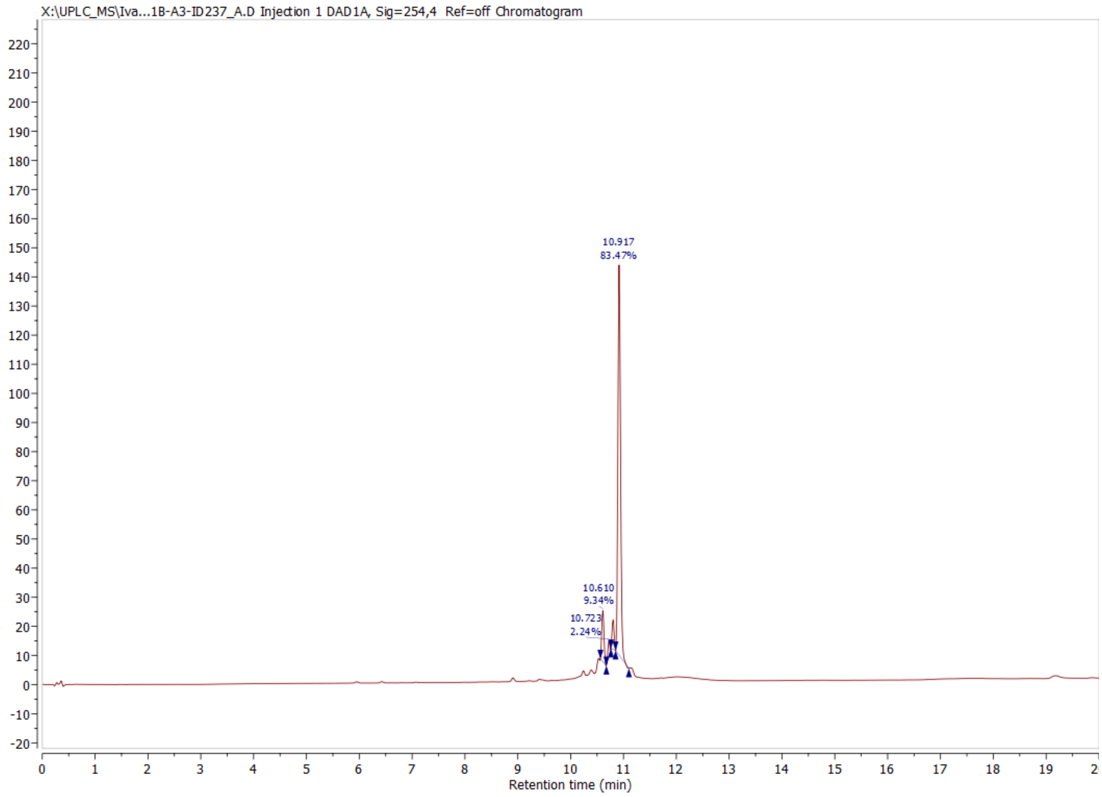

Figure S5 Absorbance chromatogram at 254 nm of ON4*.

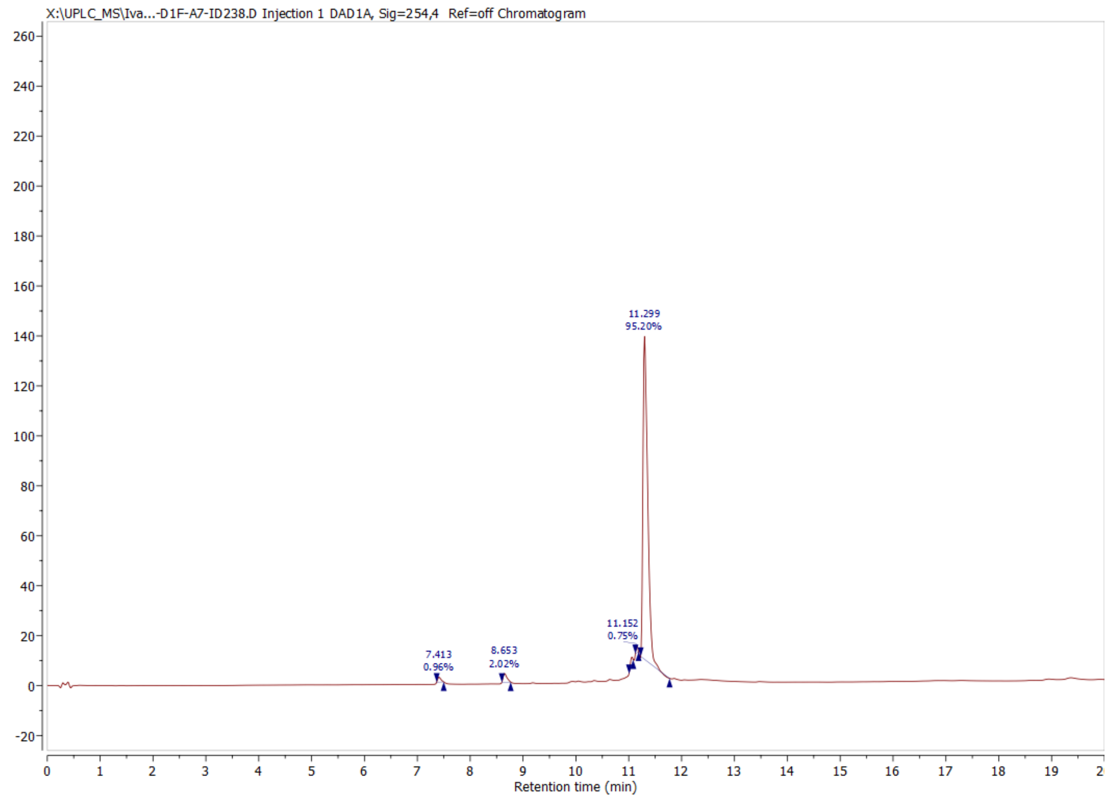

Figure S6 Absorbance chromatogram at 254 nm of ON5*.

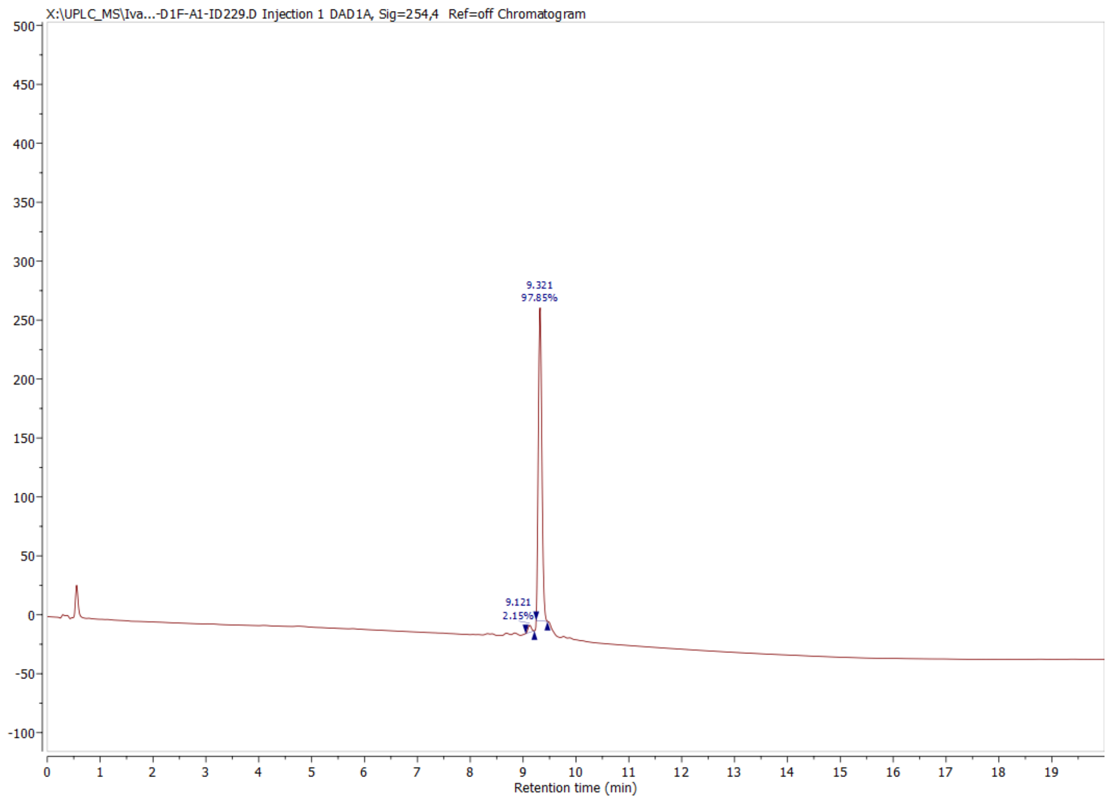

Figure S7 Absorbance chromatogram at 254 nm of ON6*.

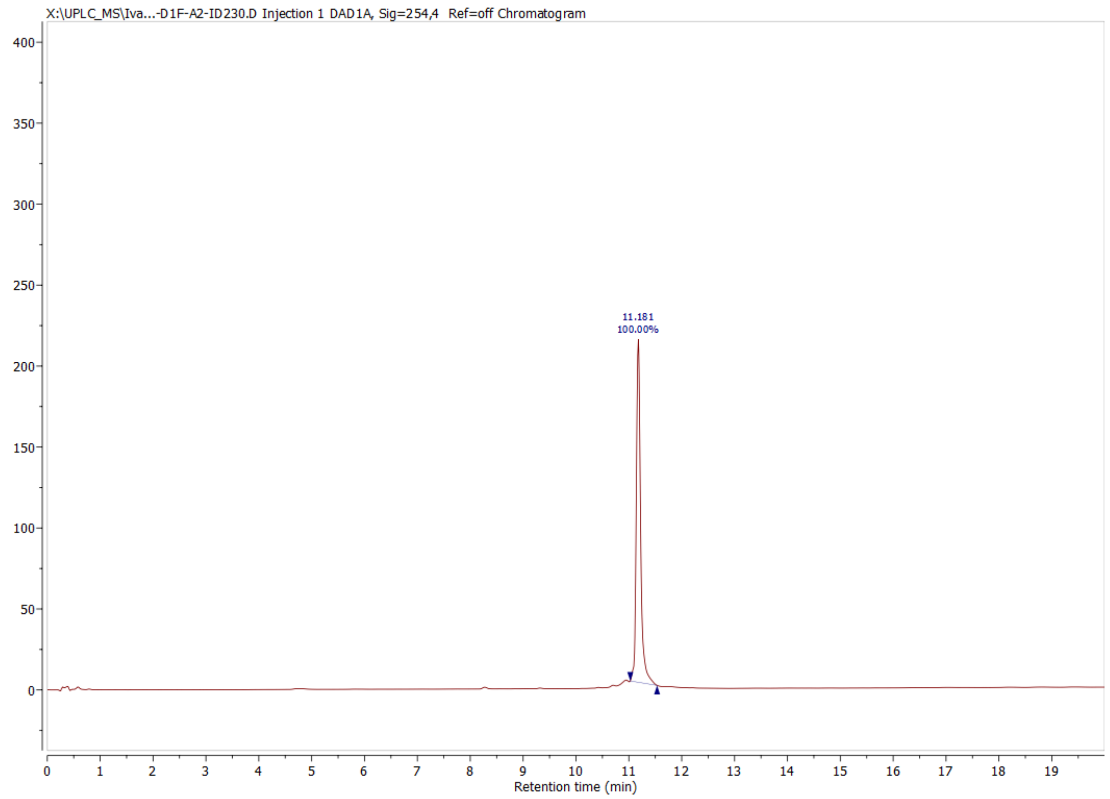

Figure S8 Absorbance chromatogram at 254 nm of ON7*.

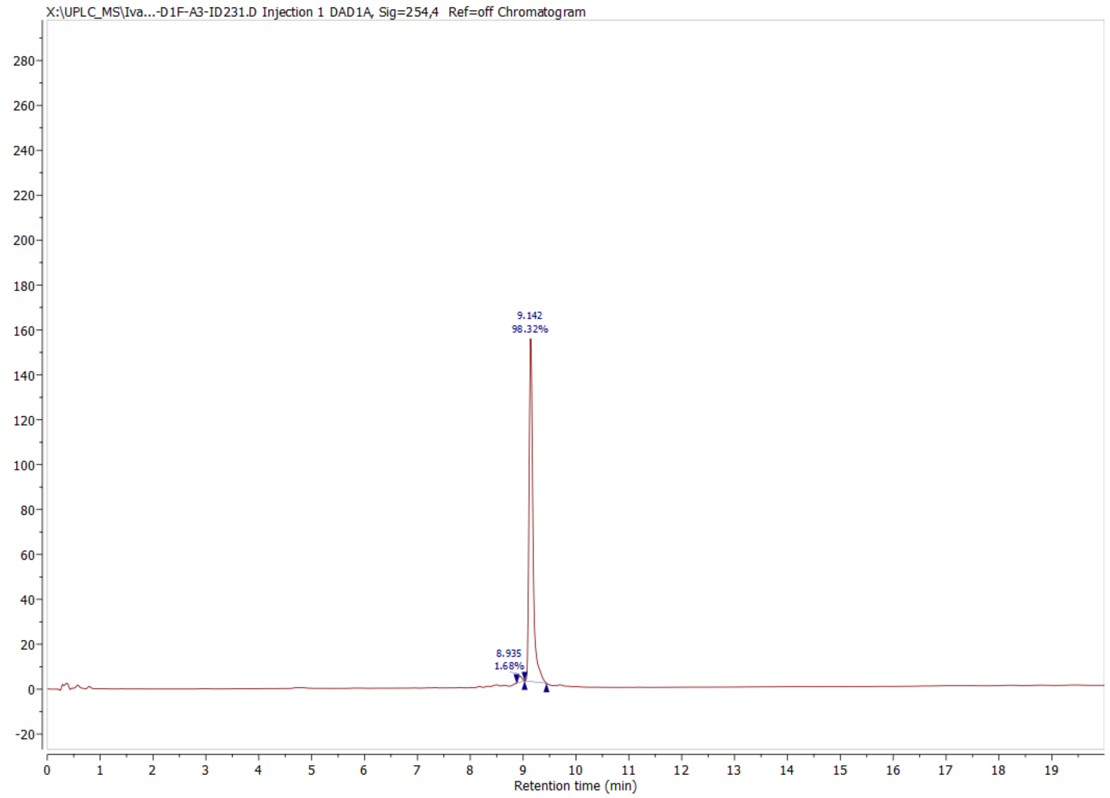

Figure S9 Absorbance chromatogram at 254 nm of ON8*.

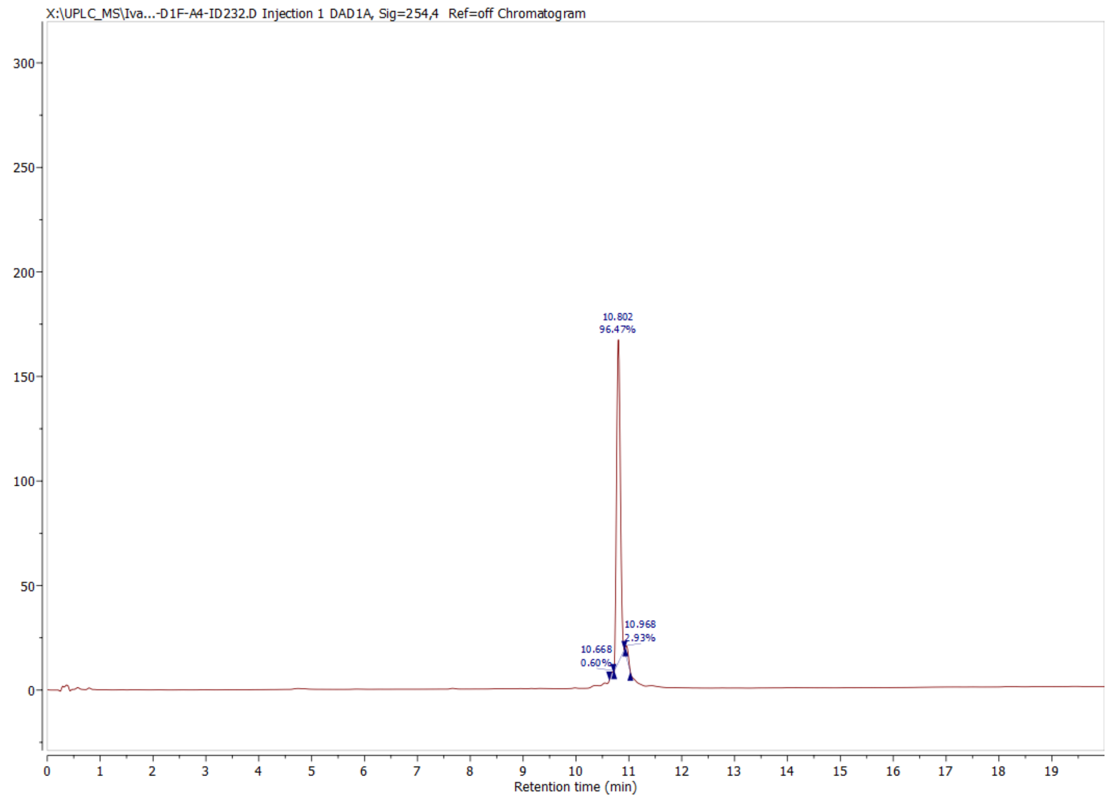

Figure S10 Absorbance chromatogram at 254 nm of ON9*.

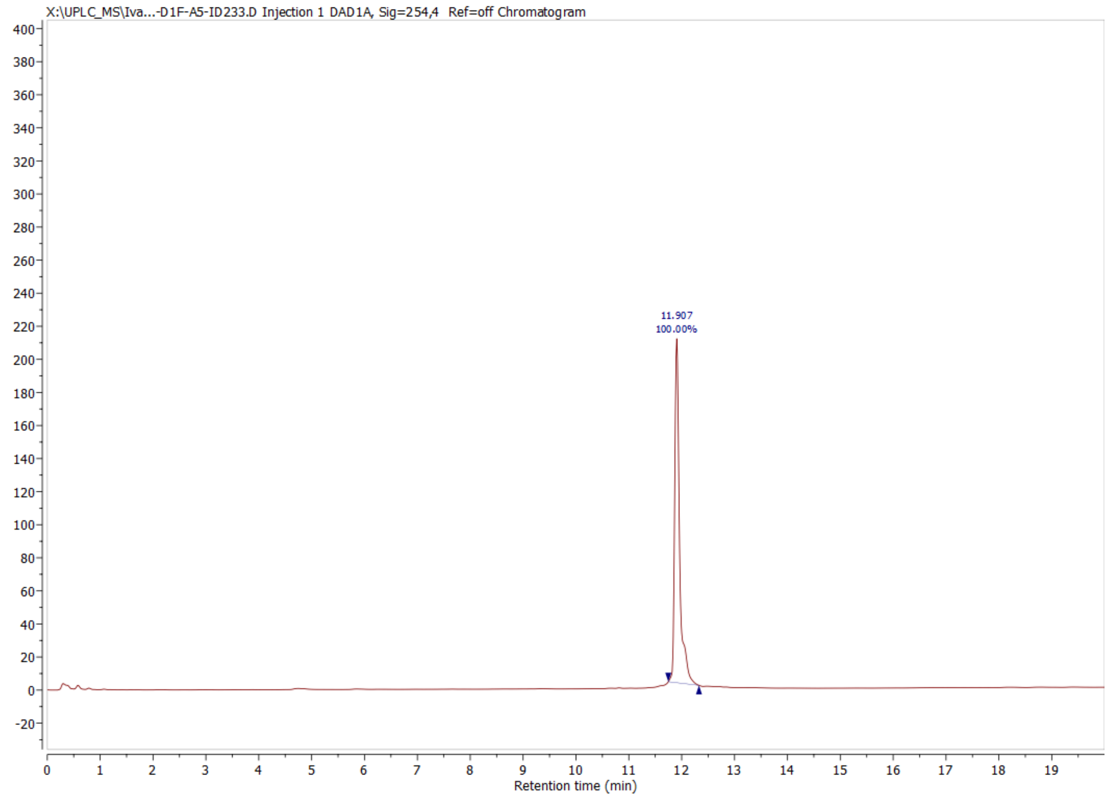

Figure S11 Absorbance chromatogram at 254 nm of ON10*.

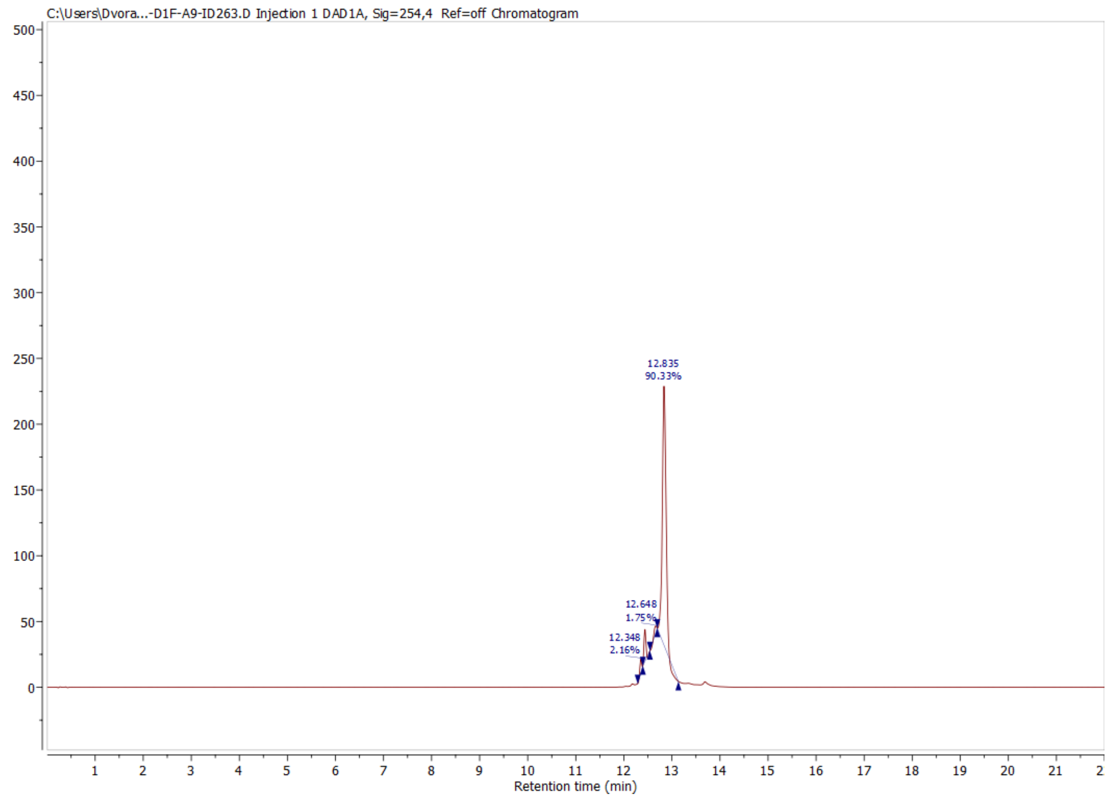

Figure S12 Absorbance chromatogram at 254 nm of ON11*.

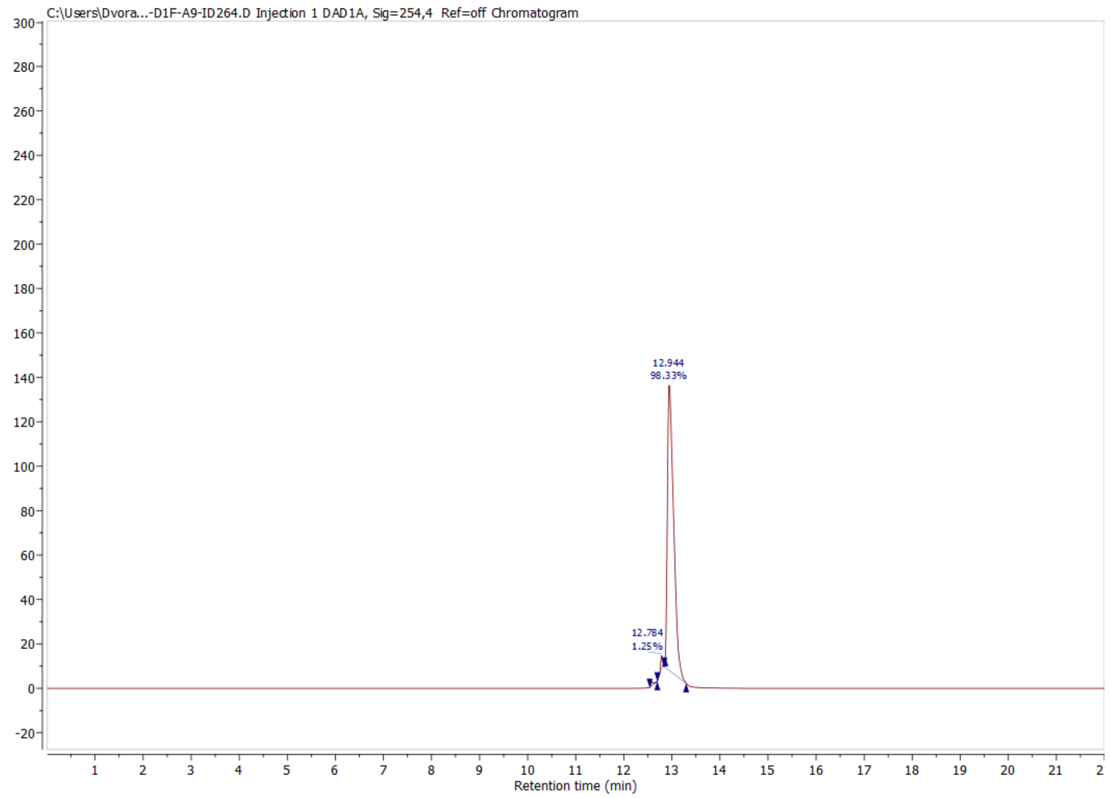

Figure S13 Absorbance chromatogram at 254 nm of ON12*.

4.2 ESI Spectra of Oligonucletotides

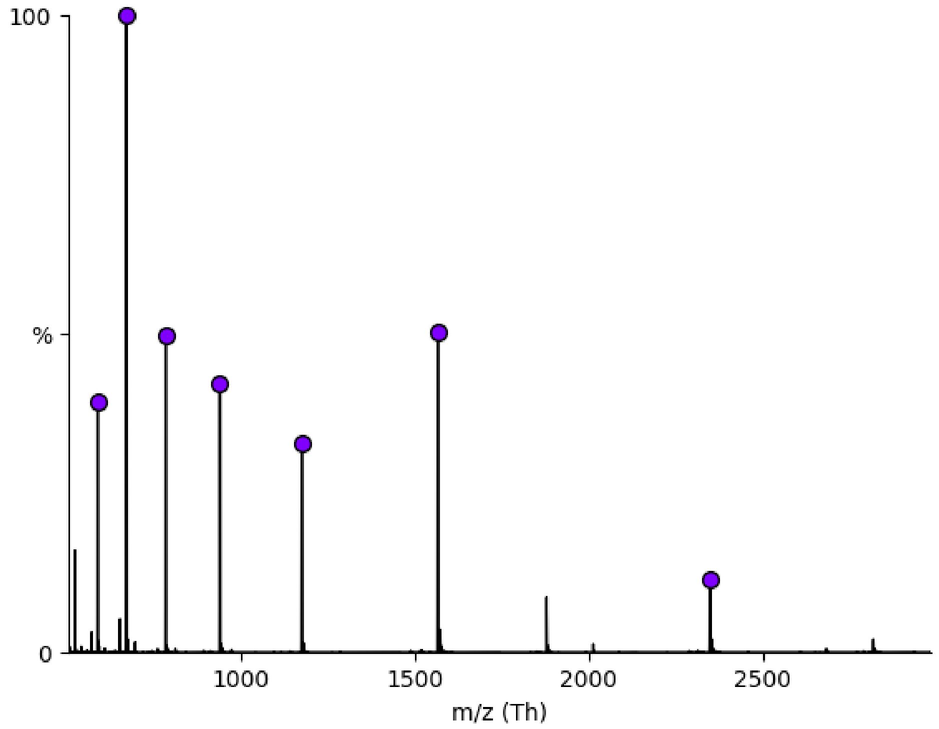

Figure S14 Raw MS spectrum of ON1*.

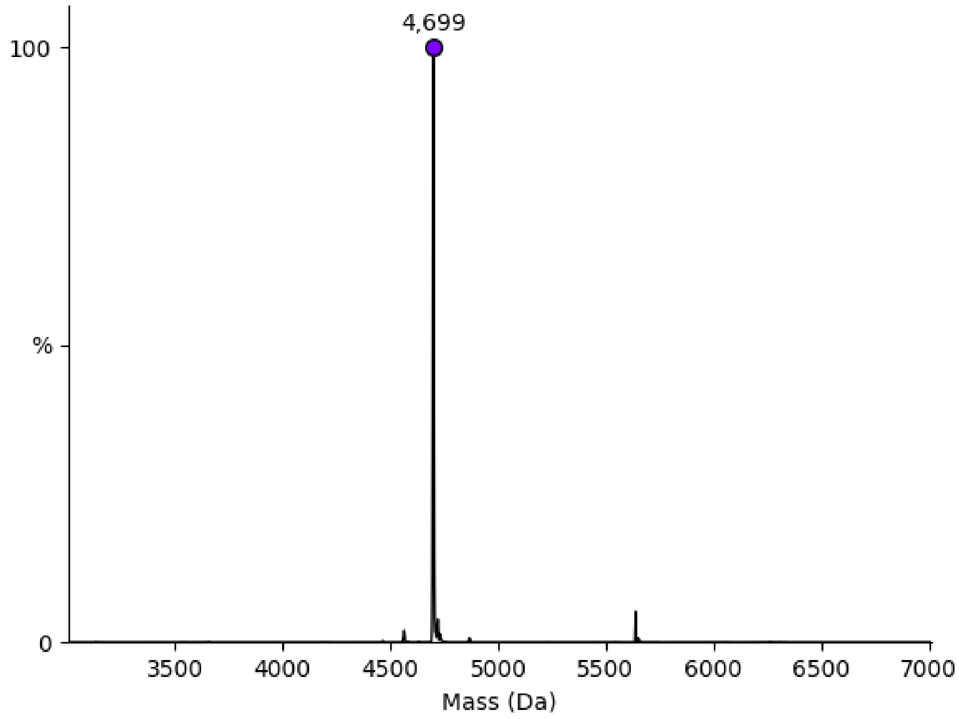

Figure S15 Deconvoluted MS spectrum of ON1*: calculated: 4699.17 Da; found: 4699 Da, Δ = 0.17 Da.

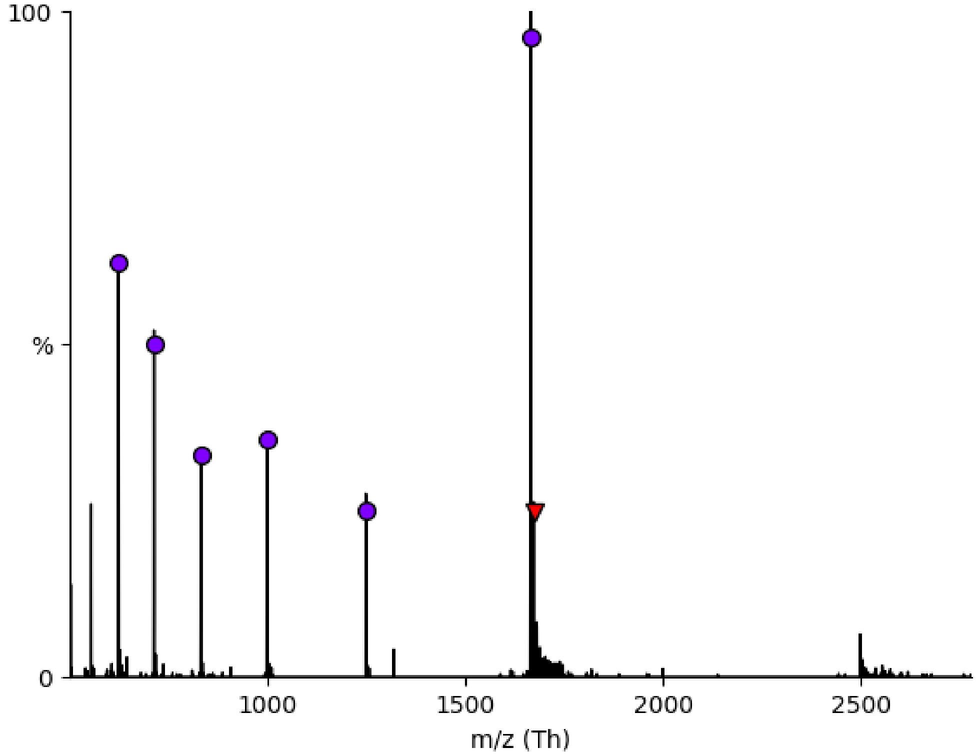

Figure S16 Raw MS spectrum of ON2*.

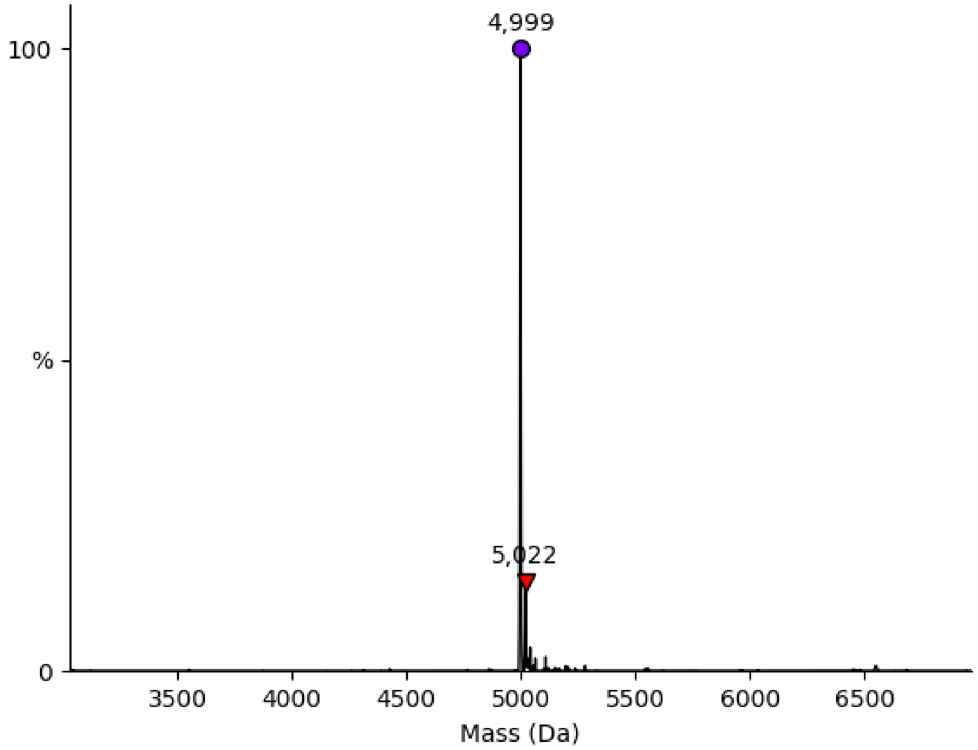

Figure S17 Deconvoluted MS spectrum of ON2*: calculated: 4999.61 Da; found: 4999 Da, Δ = 0.61 Da.

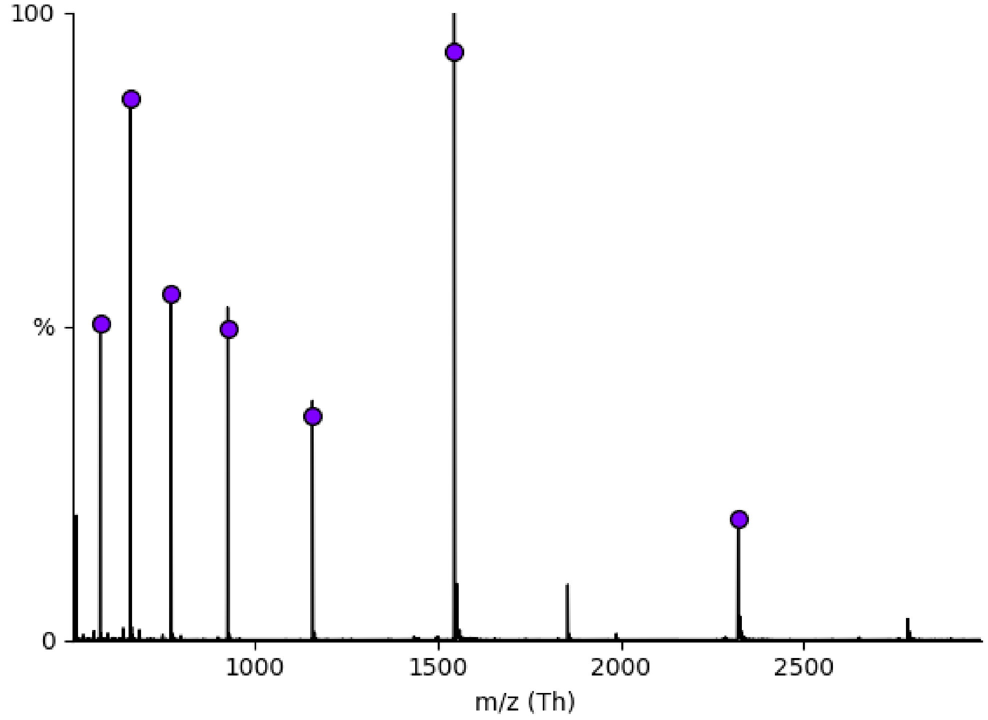

Figure S18 Raw MS spectrum of ON3*.

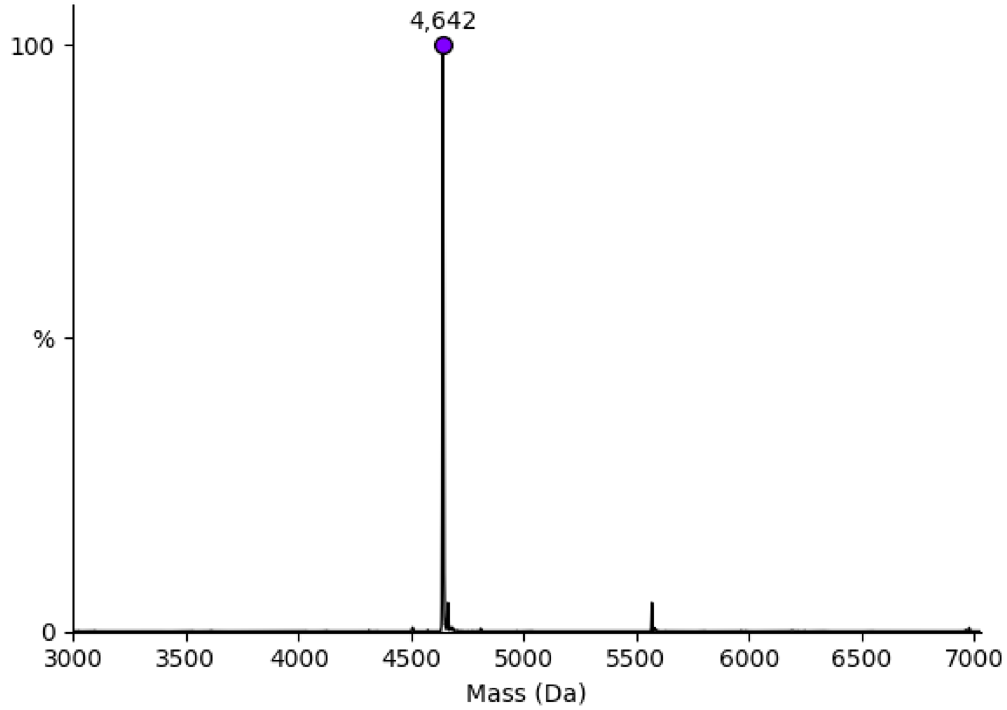

Figure S19 Deconvoluted MS spectrum of ON3*: calculated: 4642.12 Da; found: 4642 Da, Δ = 0.12 Da.

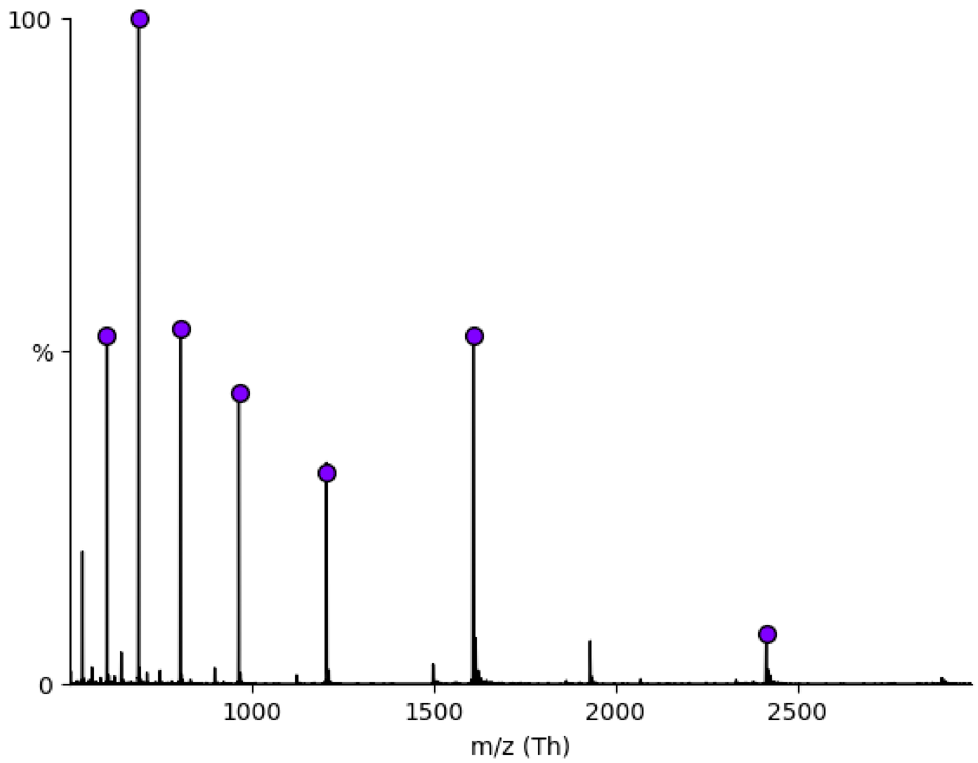

Figure S20 Raw MS spectrum of ON4*.

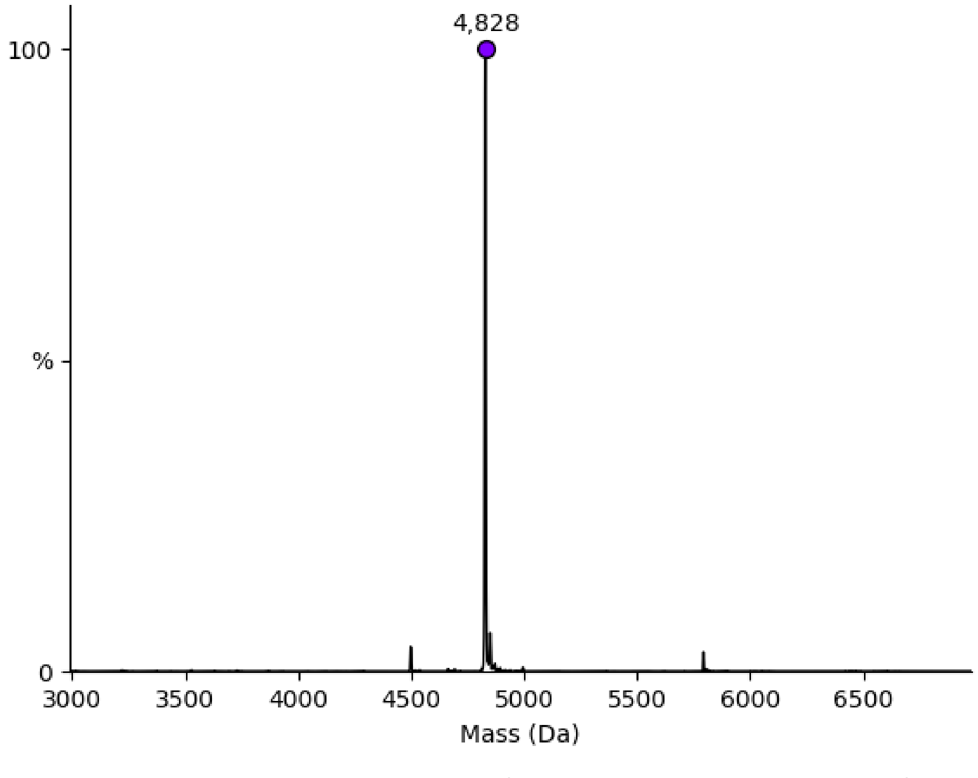

Figure S21 Deconvoluted MS spectrum of ON4*: calculated: 4828.45 Da; found: 4828 Da, Δ = 0.45 Da.

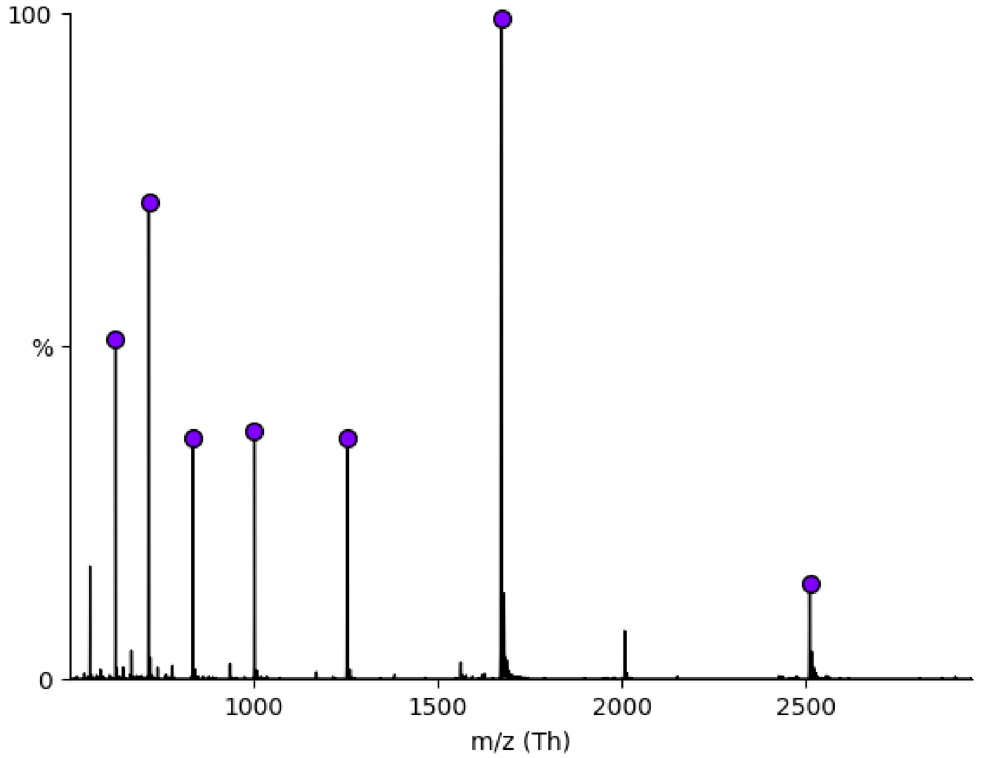

Figure S22 Raw MS spectrum of ON5*.

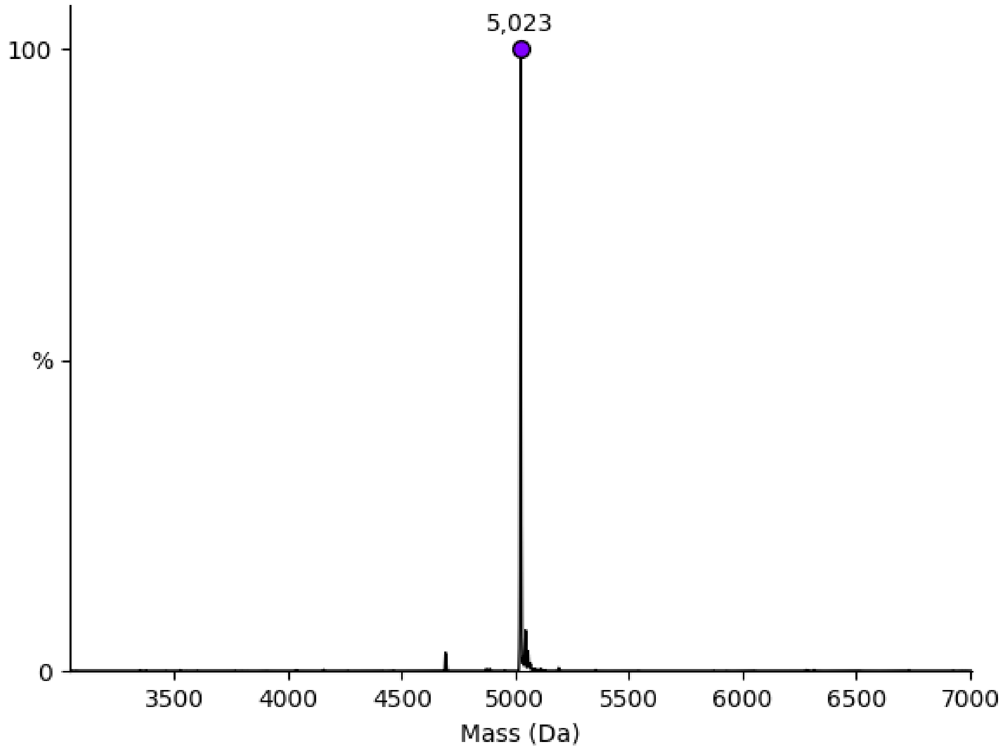

Figure S23 Deconvoluted MS spectrum of ON5*: calculated: 5023.67 Da; found: 5023 Da, Δ = 0.67 Da.

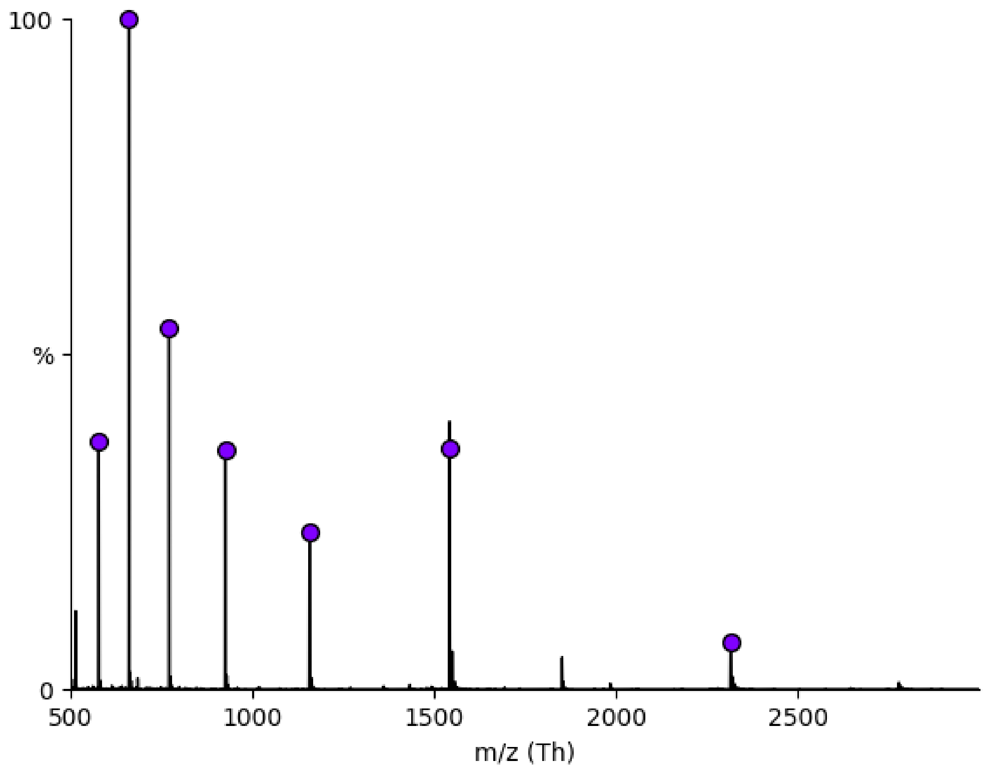

Figure S24 Raw MS spectrum of ON6*.

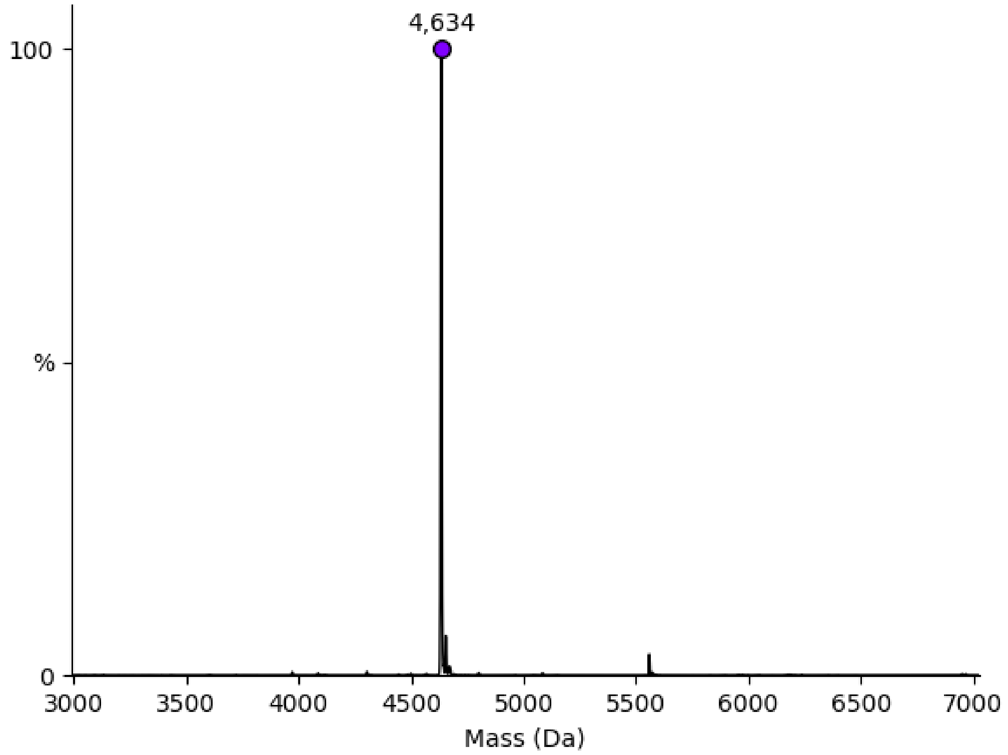

Figure S25 Deconvoluted MS spectrum of ON6*: calculated: 4634.10 Da; found: 4634 Da, Δ = 0.10 Da.

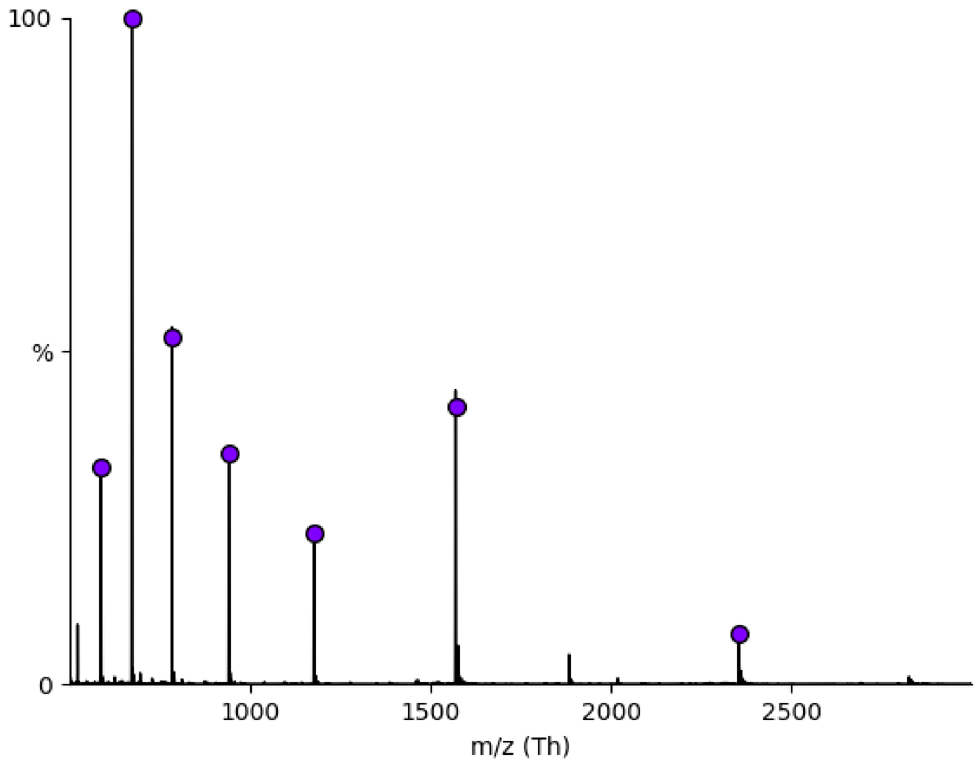

Figure S26 Raw MS spectrum of ON7*.

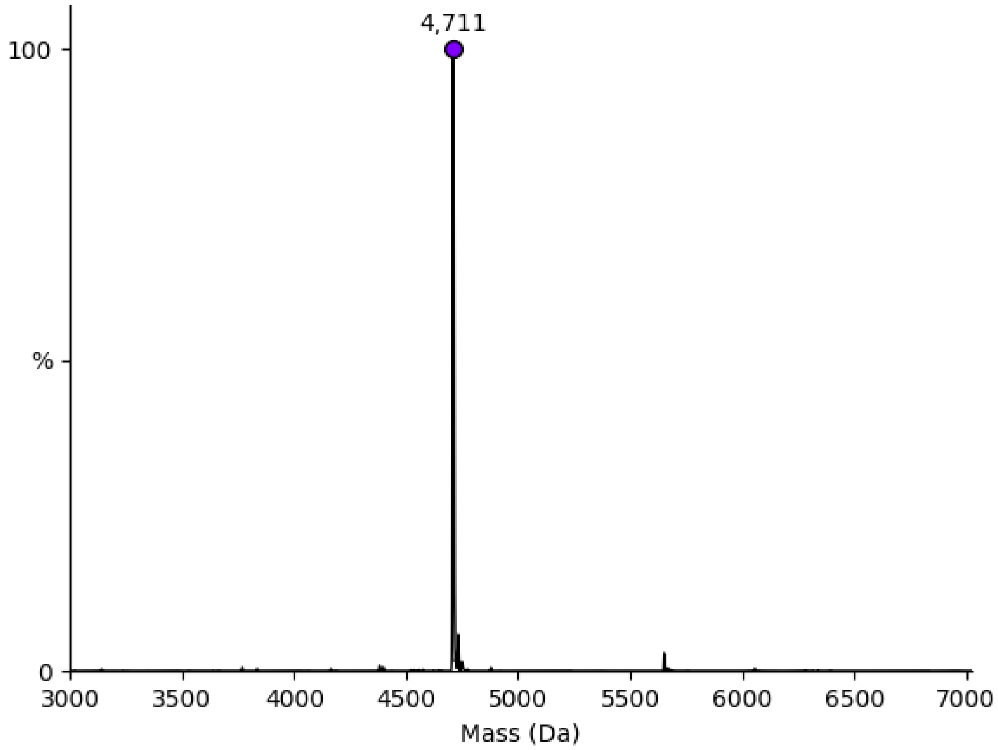

Figure S27 Deconvoluted MS spectrum of ON7*: calculated: 4711.31 Da; found: 4711 Da, Δ = 0.31 Da.

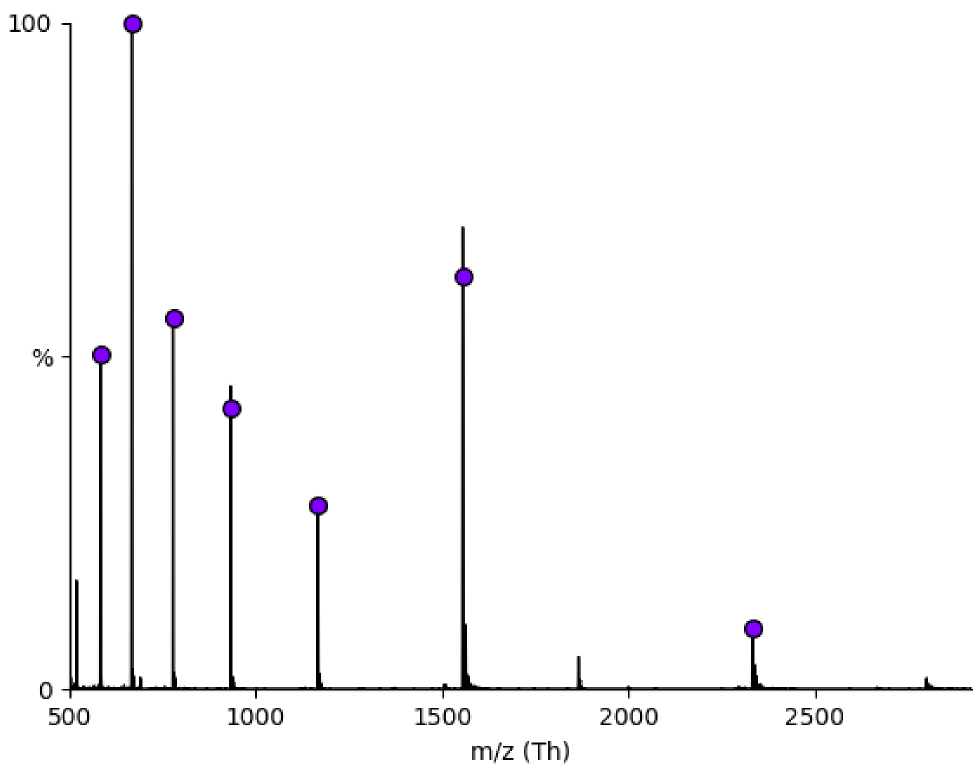

Figure S28 Raw MS spectrum of ON8*.

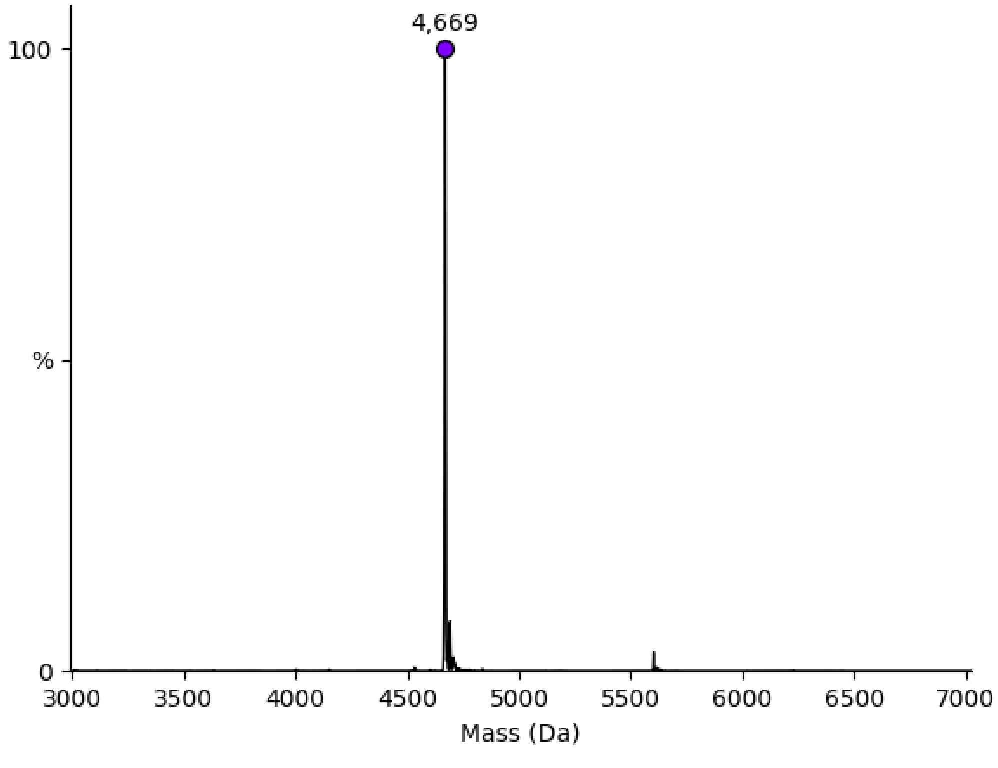

Figure S29 Deconvoluted MS spectrum of ON8*: calculated: 4669.19 Da; found: 4669 Da, Δ = 0.19 Da.

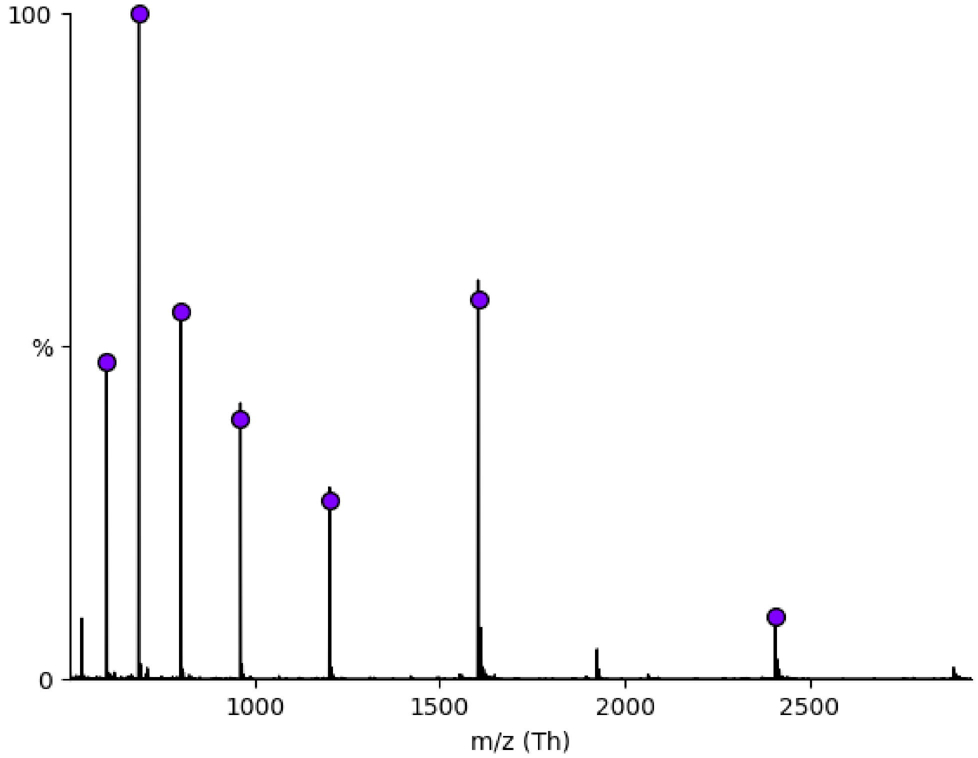

Figure S30 Raw MS spectrum of ON9*.

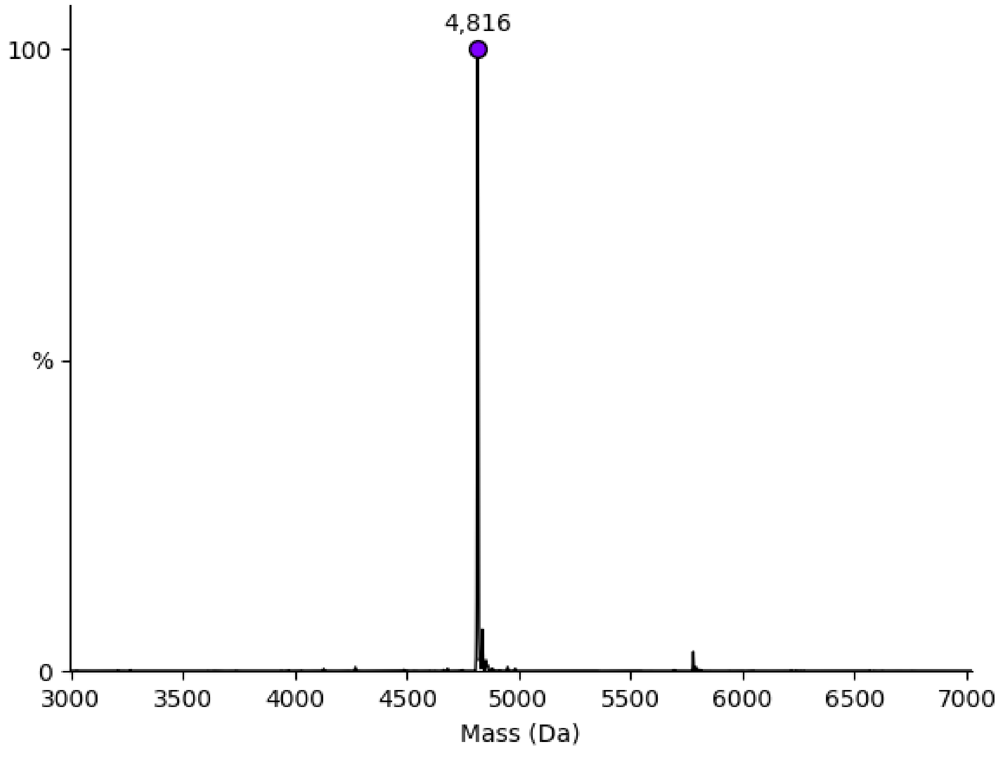

Figure S31 Deconvoluted MS spectrum of ON9*: calculated: 4816.28 Da; found: 4816 Da, Δ = 0.28 Da.

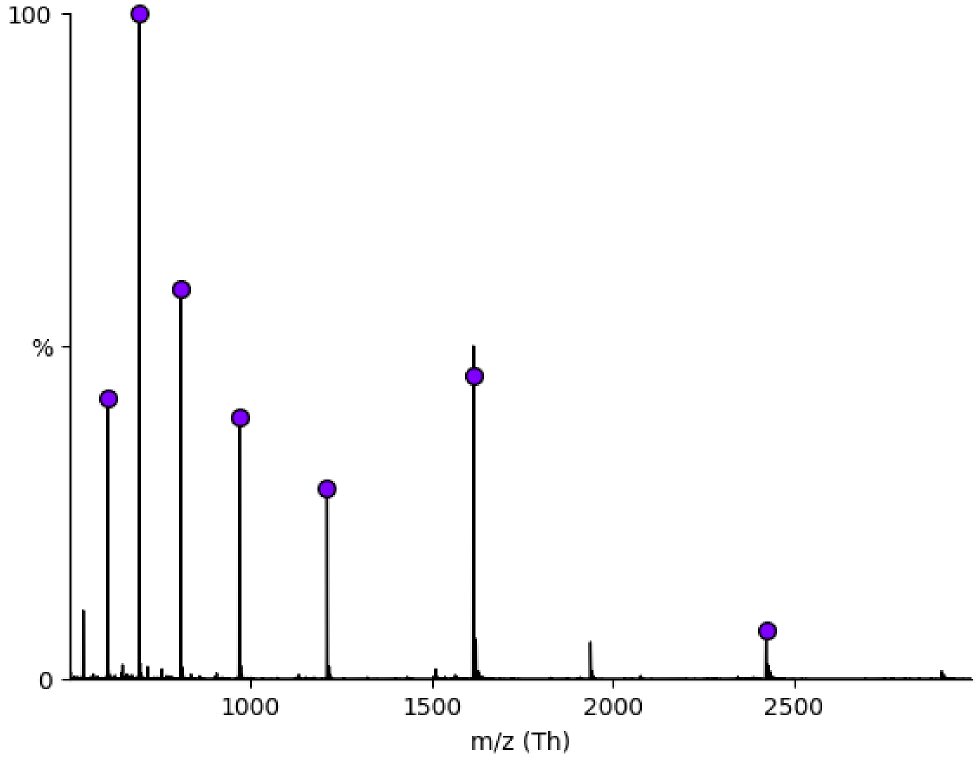

Figure S32 Raw MS spectrum of ON10*.

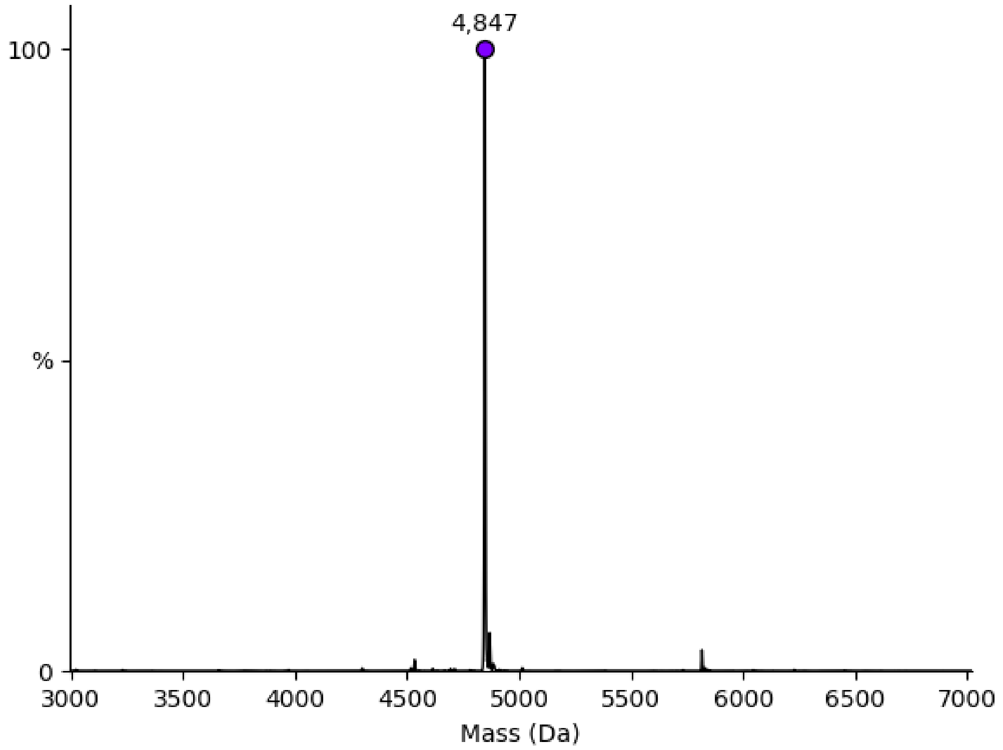

Figure S33 Deconvoluted MS spectrum of ON10*: calculated: 4847.39 Da; found: 4847 Da, Δ = 0.39 Da.

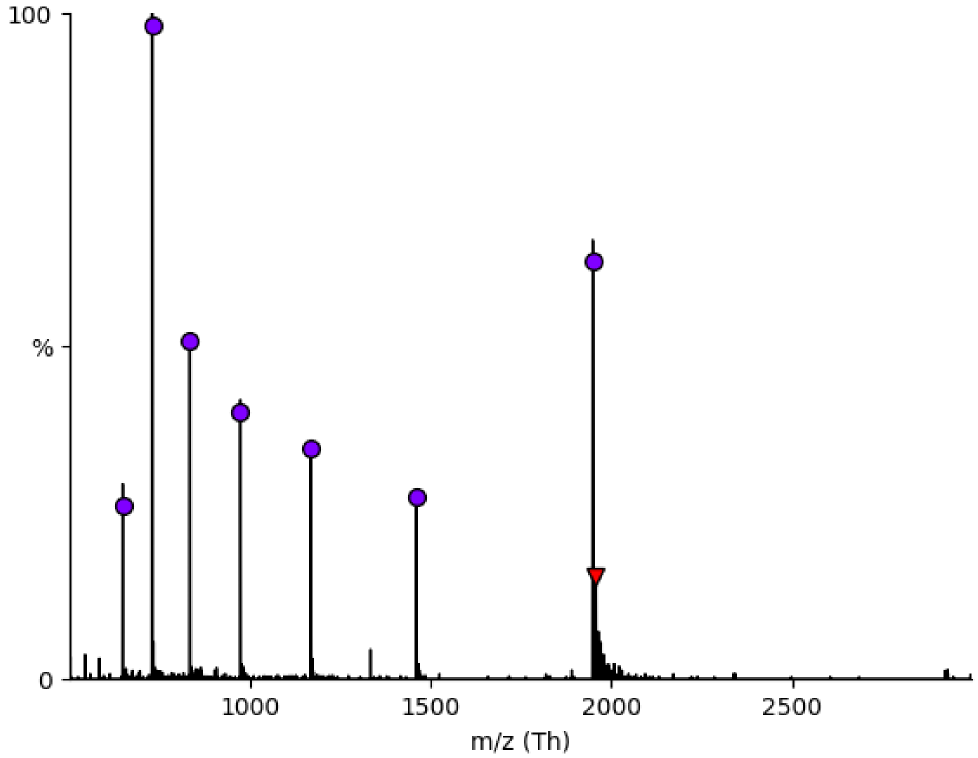

Figure S34 Raw MS spectrum of ON11*.

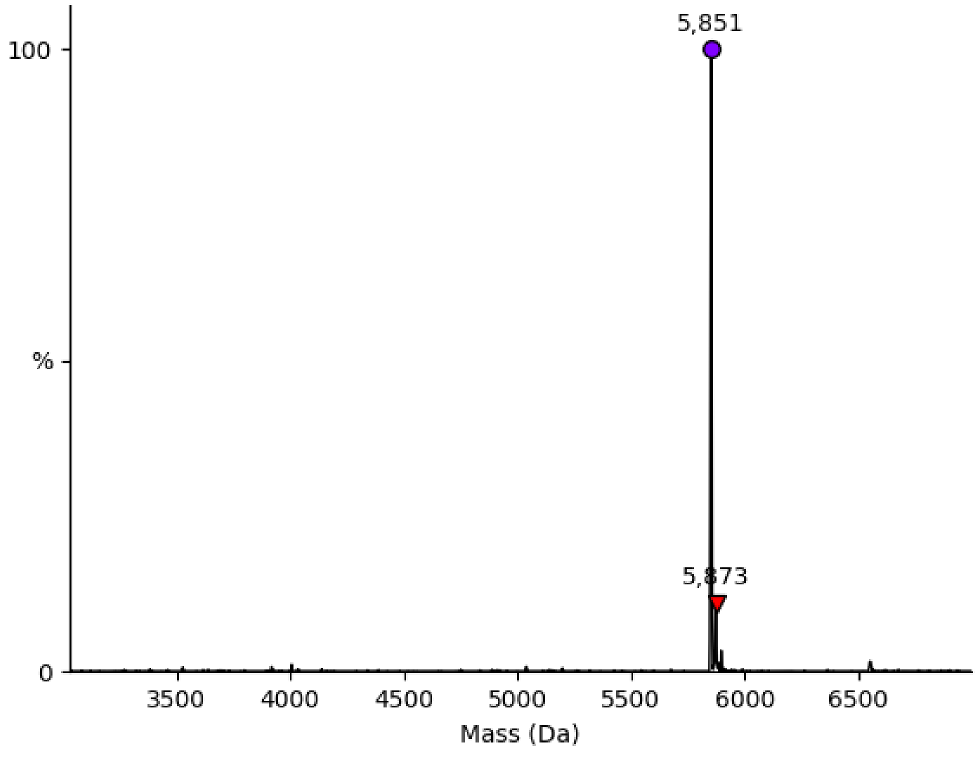

Figure S35 Deconvoluted MS spectrum of ON11*: calculated: 5851.75 Da; found: 5851 Da, Δ = 0.75 Da.

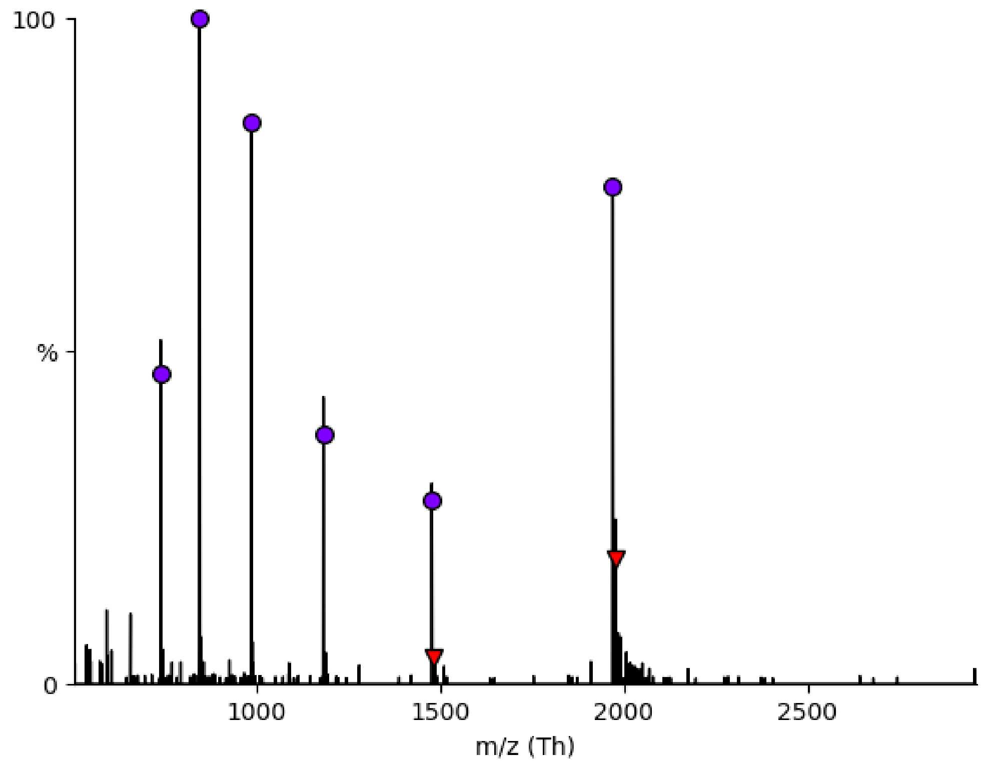

Figure S36 Raw MS spectrum of ON12*.

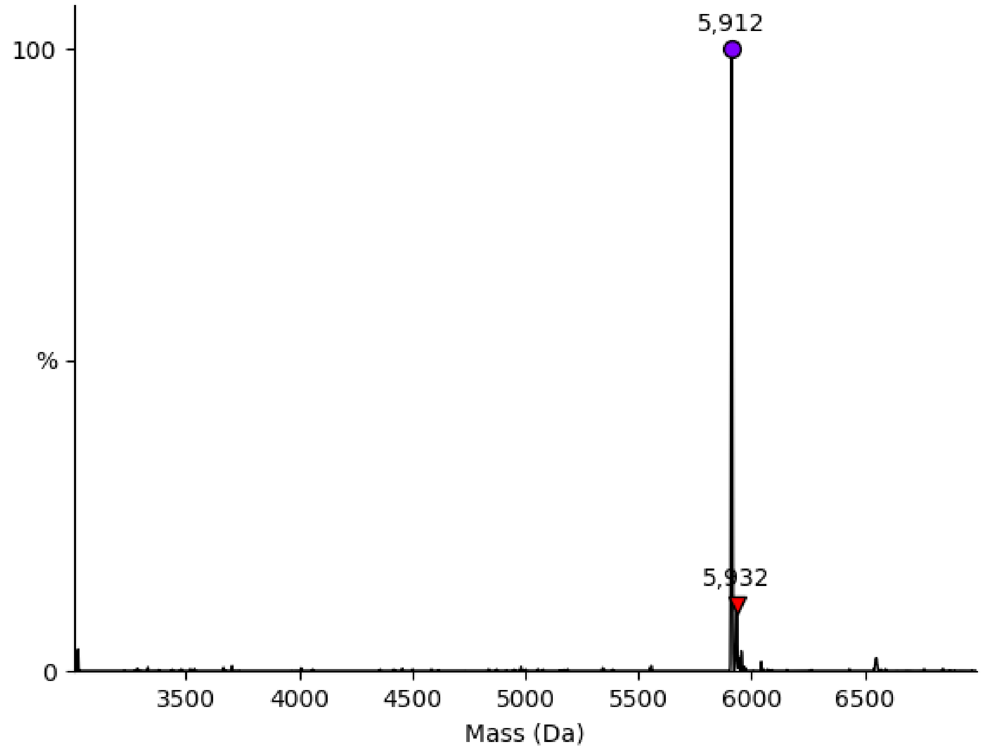

Figure S37 Deconvoluted MS spectrum of ON12*: calculated: 5912.83 Da; found: 5912 Da, Δ = 0.83 Da.

4.3 Melting and Annealing Curves

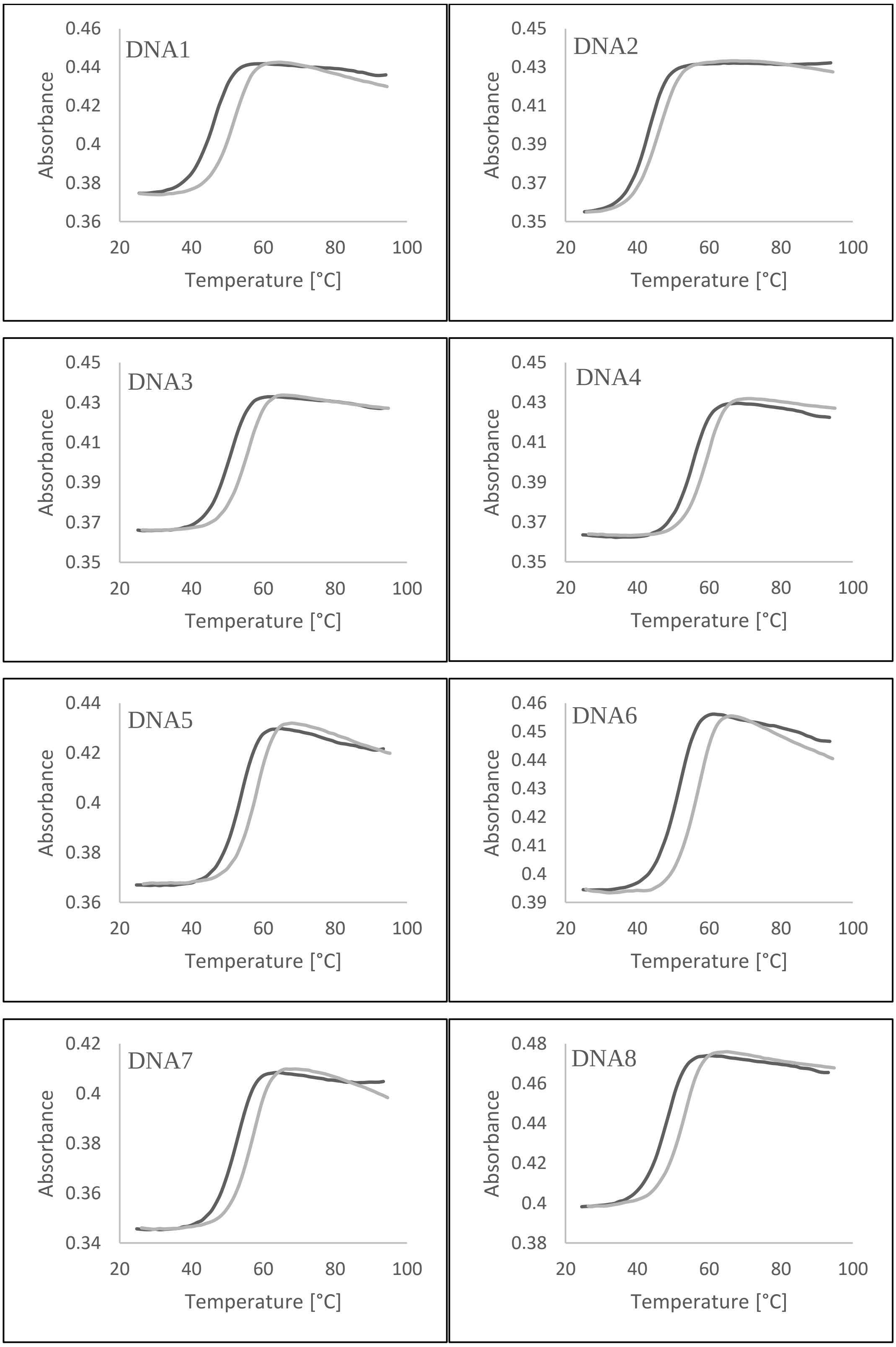

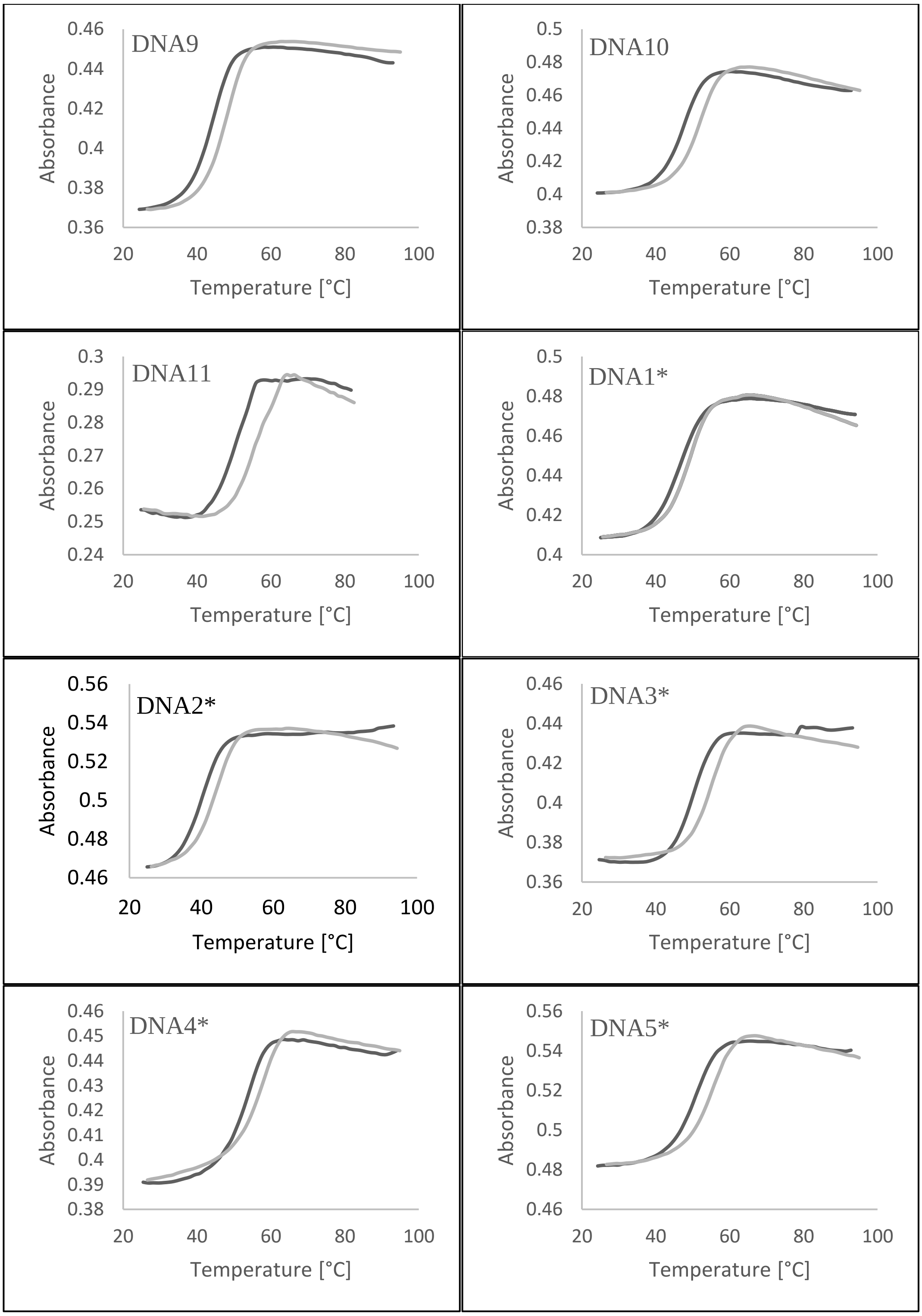
DNA9
DNA10
DNA11
DNA1*
DNA2*
DNA3*
DNA4*
DNA5*
Absorbance
Temperature [°C]

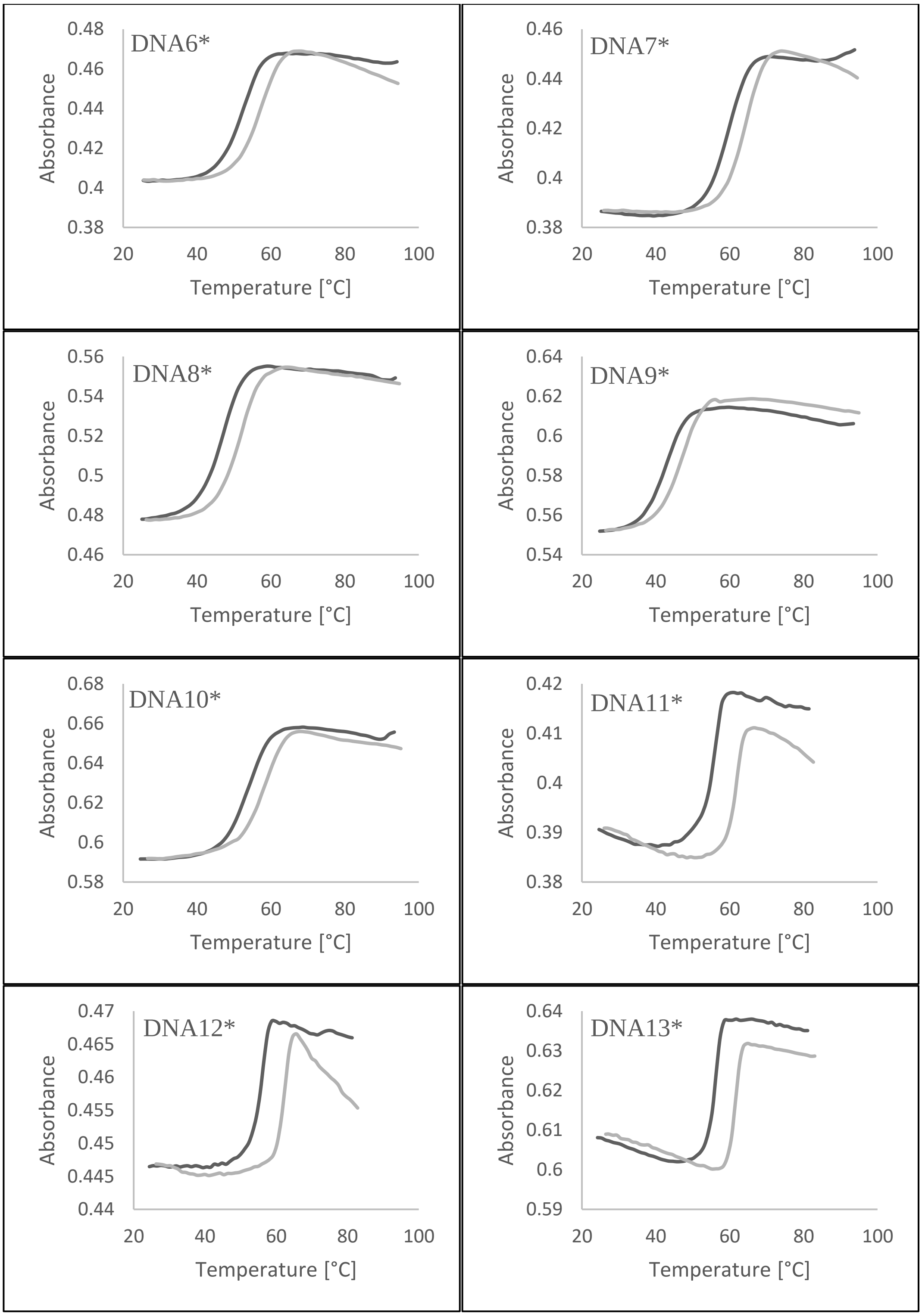
DNA6*
Absorbance
0.48
0.46
0.44
0.42
0.4
0.38
20
40
60
80
100
Temperature [°C]
DNA7*
Absorbance
0.46
0.44
0.42
0.4
0.38
20
40
60
80
100
Temperature [°C]
DNA8*
Absorbance
0.56
0.54
0.52
0.5
0.48
0.46
20
40
60
80
100
Temperature [°C]
DNA9*
Absorbance
0.64
0.62
0.6
0.58
0.56
0.54
20
40
60
80
100
Temperature [°C]
DNA10*
Absorbance
0.68
0.66
0.64
0.62
0.6
0.58
20
40
60
80
100
Temperature [°C]
DNA11*
Absorbance
0.42
0.41
0.4
0.39
0.38
20
40
60
80
100
Temperature [°C]
DNA12*
Absorbance
0.47
0.465
0.46
0.455
0.45
0.445
0.44
20
40
60
80
100
Temperature [°C]
DNA13*
Absorbance
0.64
0.63
0.62
0.61
0.6
0.59
20
40
60
80
100
Temperature [°C]

4.4 Copies of NMR Spectra

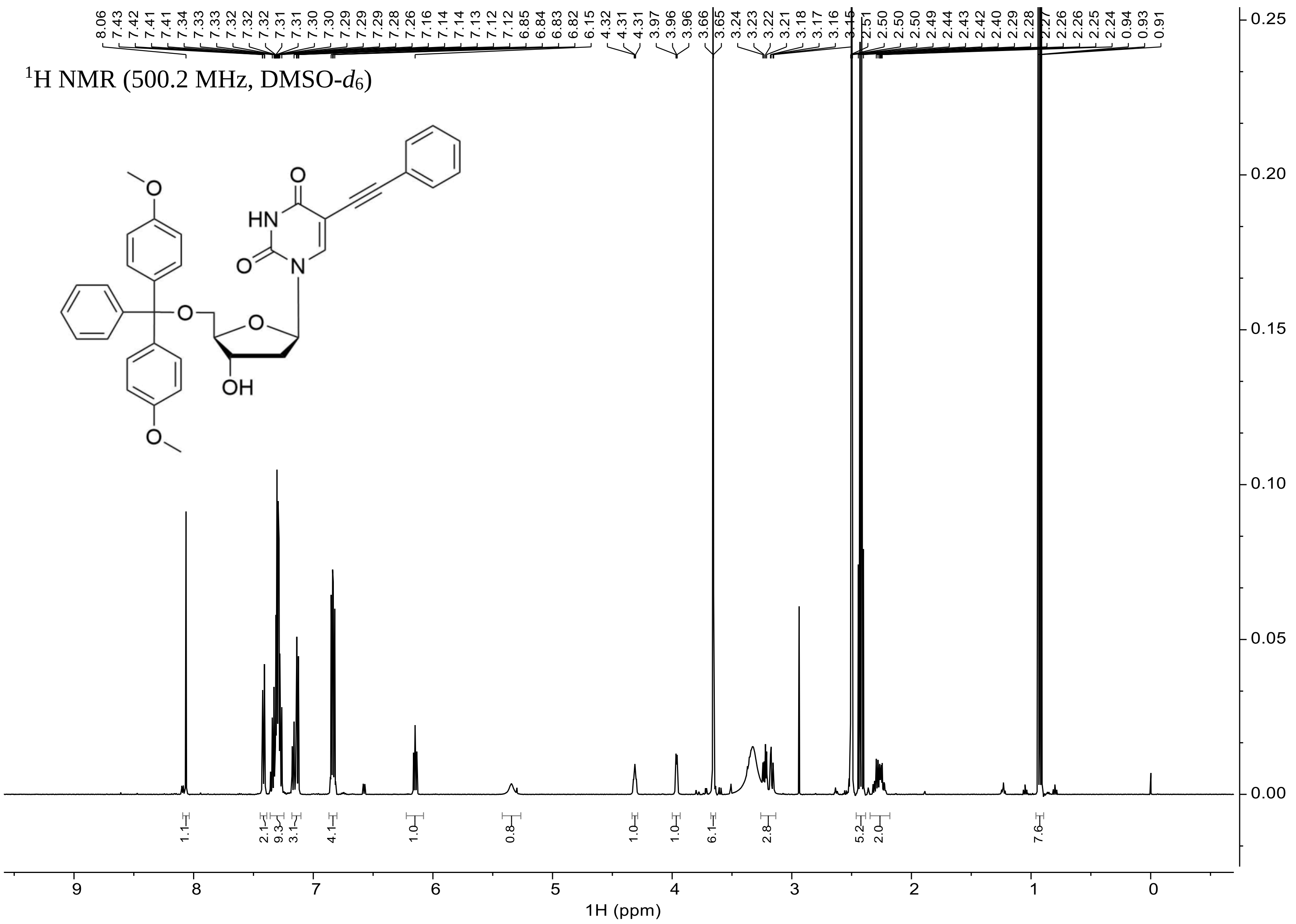

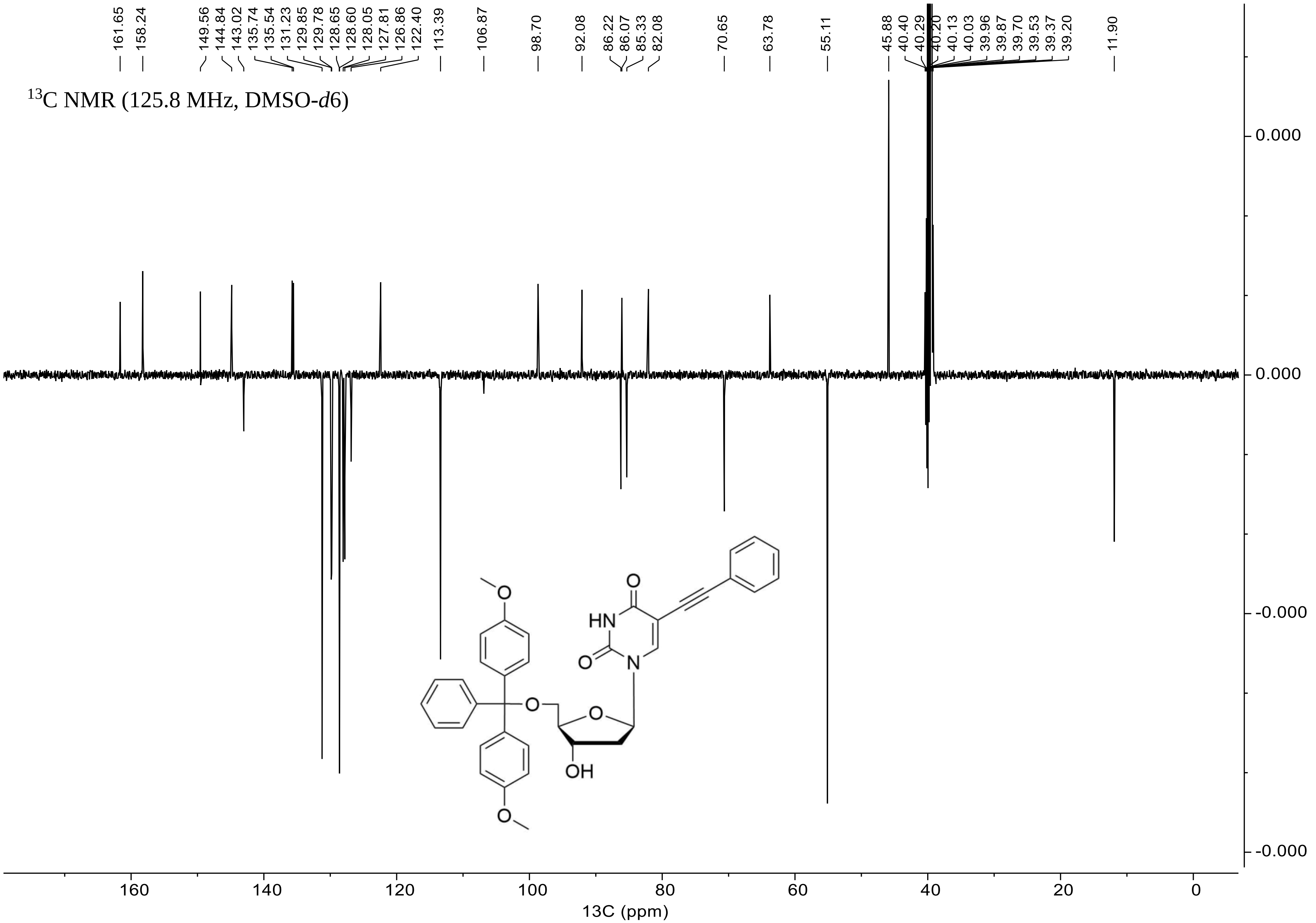

161.65
158.24
149.56
144.84
143.02
135.74
135.54
131.23
129.85
129.78
128.65
128.60
128.05
127.81
126.86
122.40
113.39
106.87
98.70
92.08
86.22
86.07
85.33
82.08
70.65
63.78
55.11
45.88
40.40
40.29
40.20
40.13
40.03
39.96
39.87
39.70
39.53
39.37
39.20
11.90
^{13}C NMR (125.8 MHz, DMSO-*d*6)
0.000
0.000
-0.000
-0.000
160
140
120
100
80
60
40
20
0
13C (ppm)
O
HN
O
N
O
O
O
OH
O

^{1}H NMR (500.2 MHz, CD_3CN)

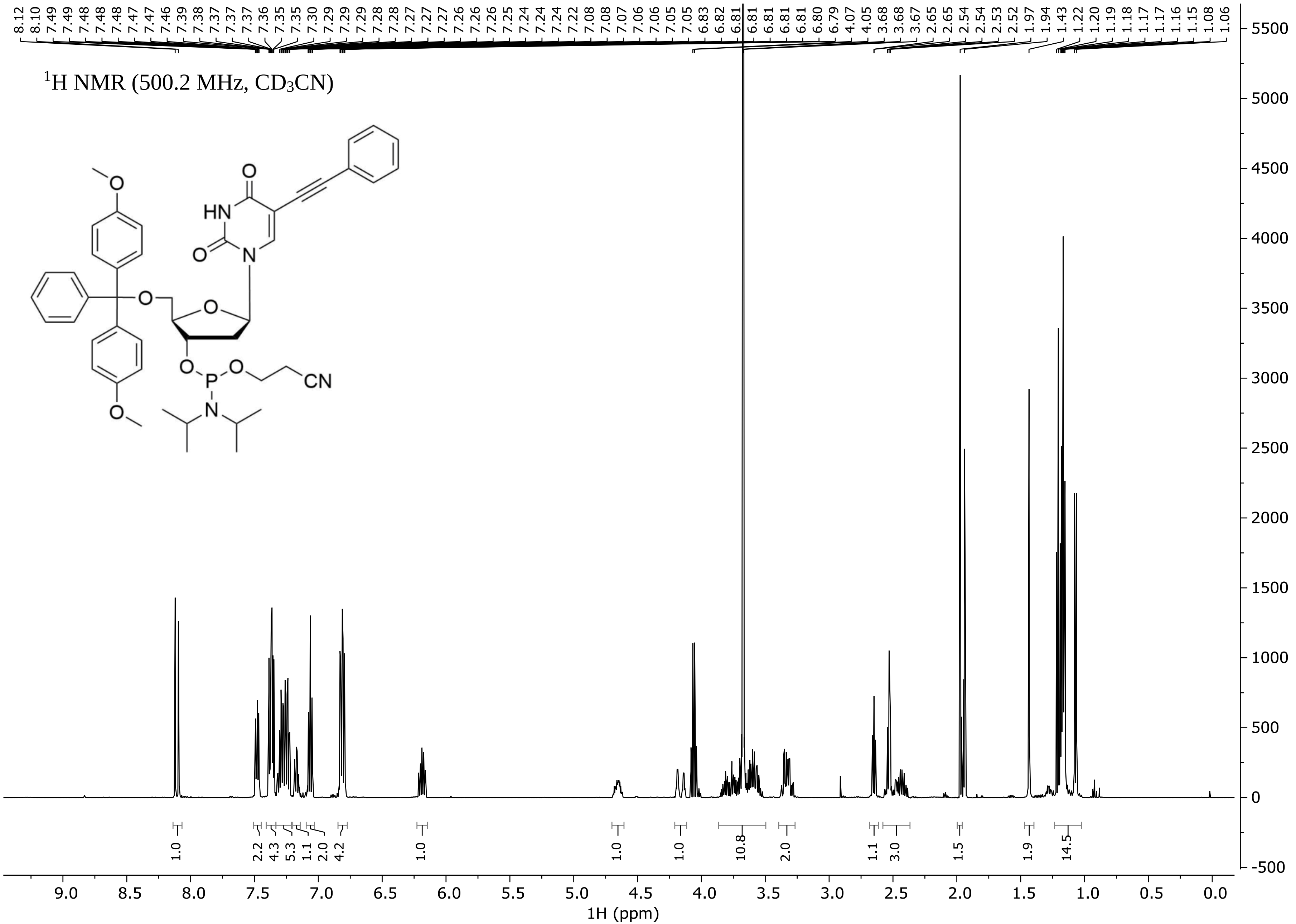

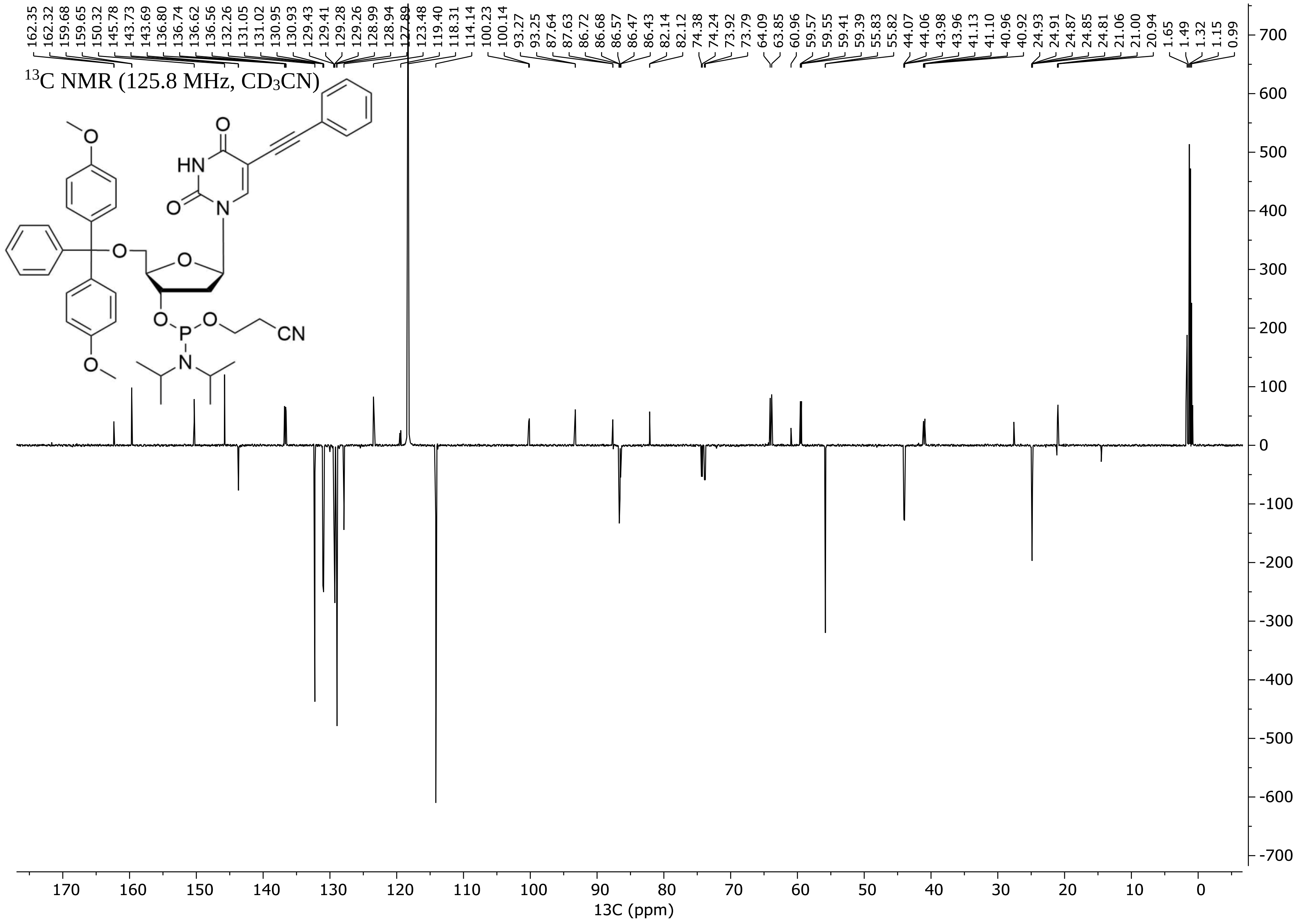

162.35
162.32
159.68
159.65
150.32
145.78
143.73
143.69
136.80
136.74
136.62
136.56
132.26
131.05
131.02
130.95
130.93
129.43
129.41
129.28
129.26
128.99
128.94
127.89
123.48
119.40
118.31
114.14
100.23
100.14
93.27
93.25
87.64
87.63
86.72
86.68
86.57
86.47
86.43
82.14
82.12
74.38
74.24
73.92
73.79
64.09
63.85
60.96
59.57
59.55
59.41
59.39
55.83
55.82
44.07
44.06
43.98
43.96
41.13
41.10
40.96
40.92
24.93
24.91
24.87
24.85
24.81
21.06
21.00
20.94
1.65
1.49
1.32
1.15
0.99
^{13}C NMR (125.8 MHz, CD_3CN)
HN
O
N
O
O
O
O
O
P
N
CN
13C (ppm)

$^{31}P\{^{1}H\}$ NMR (202.4 MHz, CD_3CN)

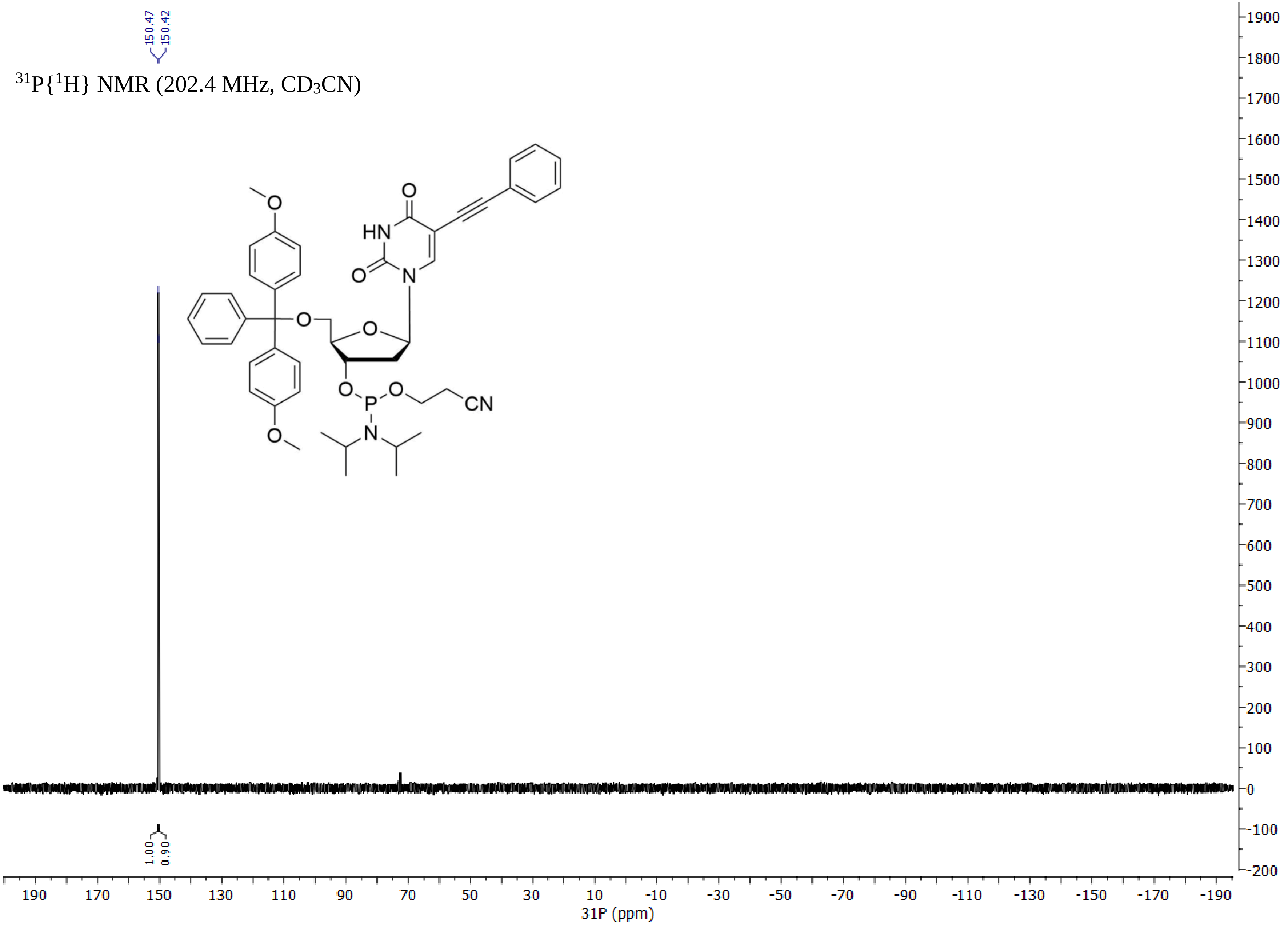

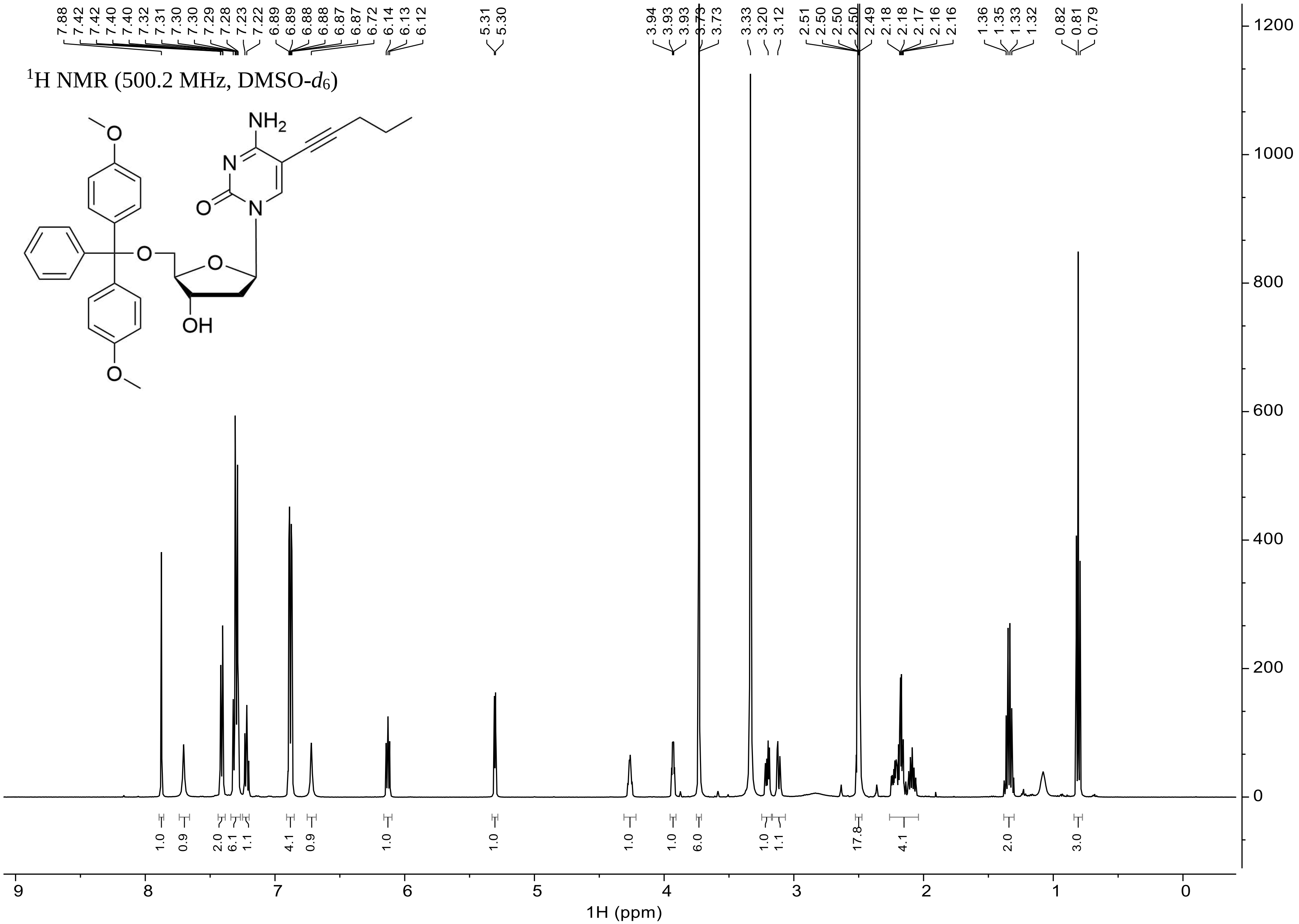

^{1}H NMR (500.2 MHz, DMSO-d_6)
1H (ppm)

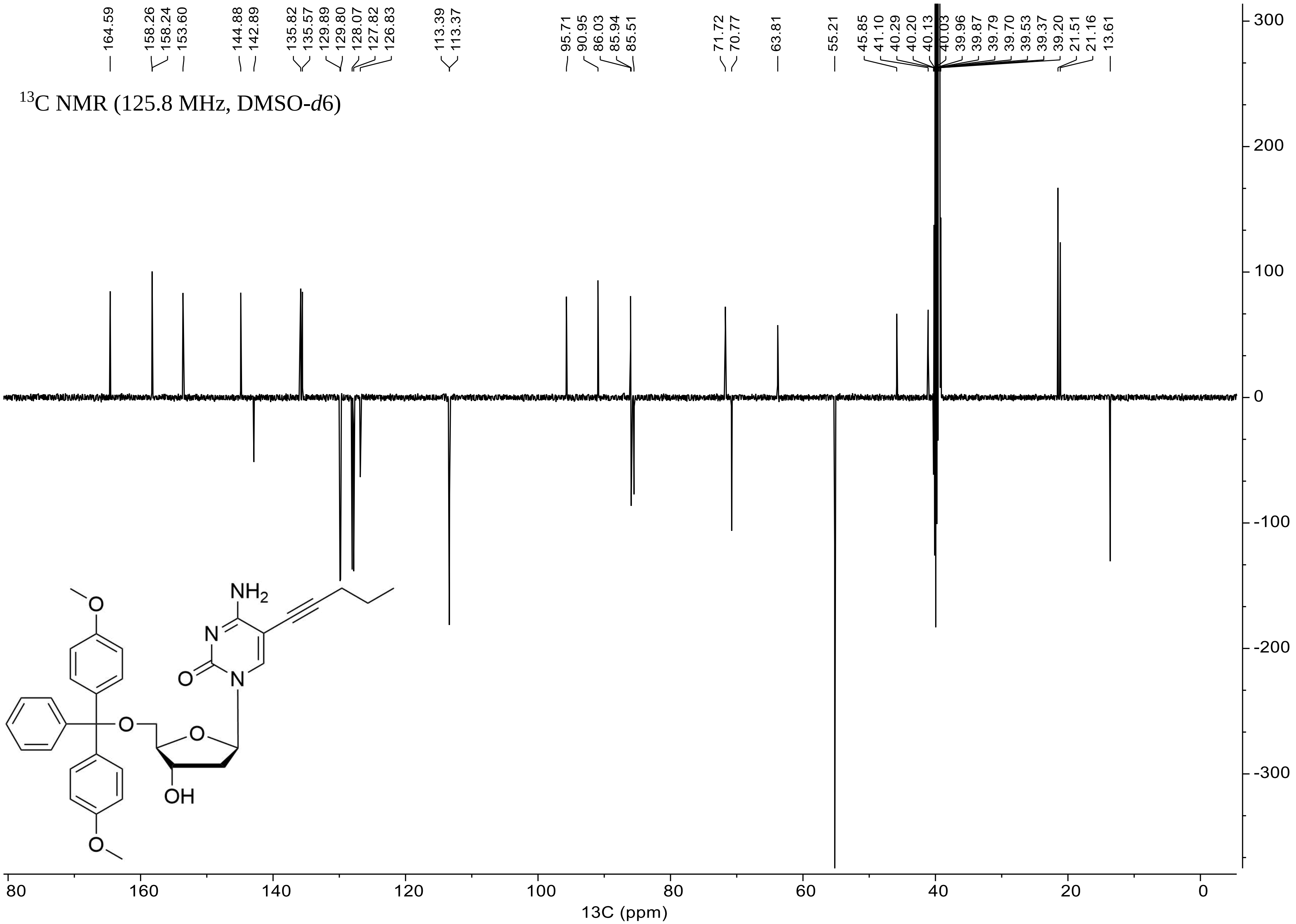
164.59
158.26
158.24
153.60
144.88
142.89
135.82
135.57
129.89
129.80
128.07
127.82
126.83
113.39
113.37
95.71
90.95
86.03
85.94
85.51
71.72
70.77
63.81
55.21
45.85
41.10
40.29
40.20
40.13
40.03
39.96
39.87
39.79
39.70
39.53
39.37
39.20
21.51
21.16
13.61
^{13}C NMR (125.8 MHz, DMSO-*d*6)
13C (ppm)
NH2
N
O
N
O
O
O
O
OH

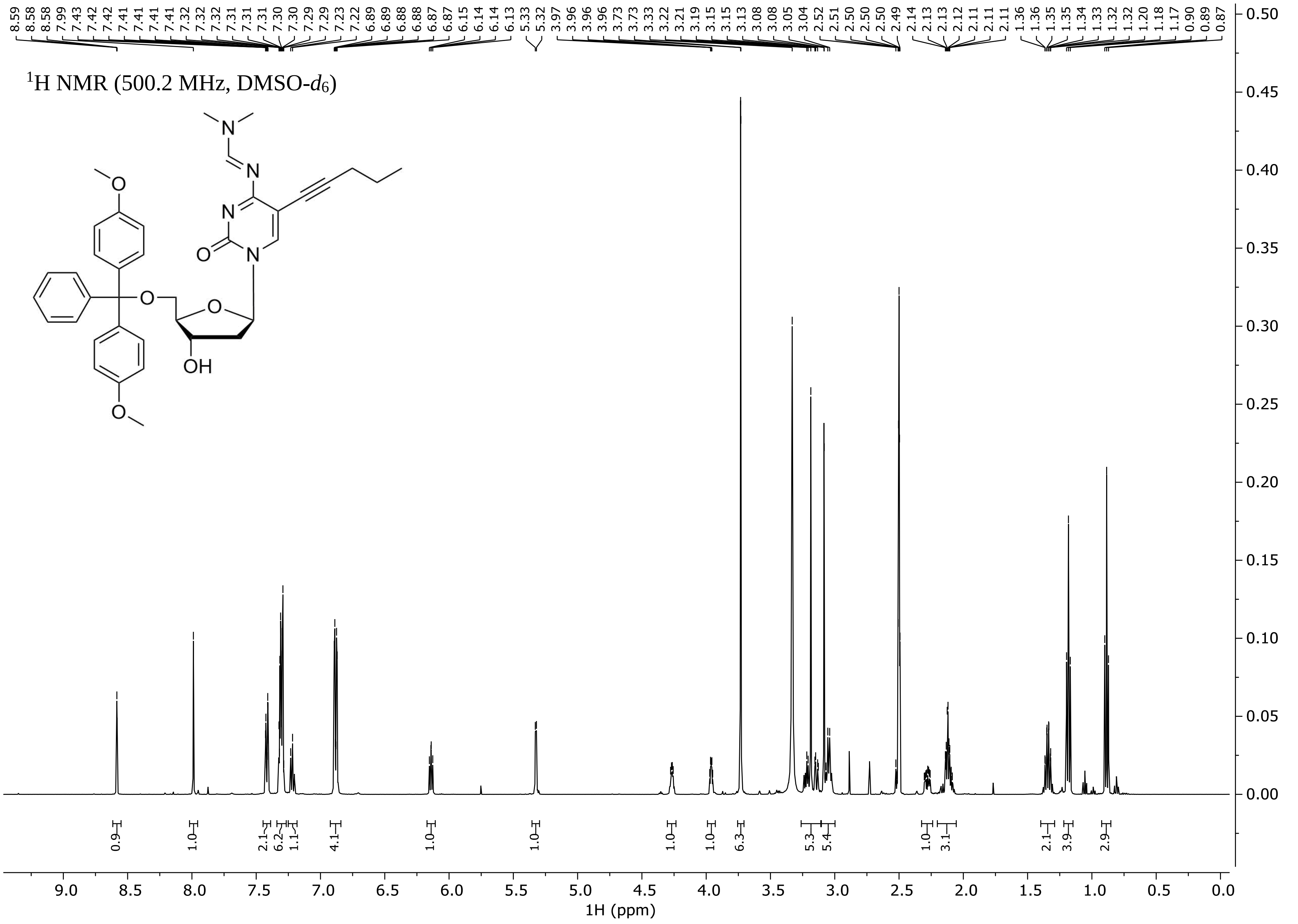

1H NMR (500.2 MHz, DMSO-d6)
1H (ppm)

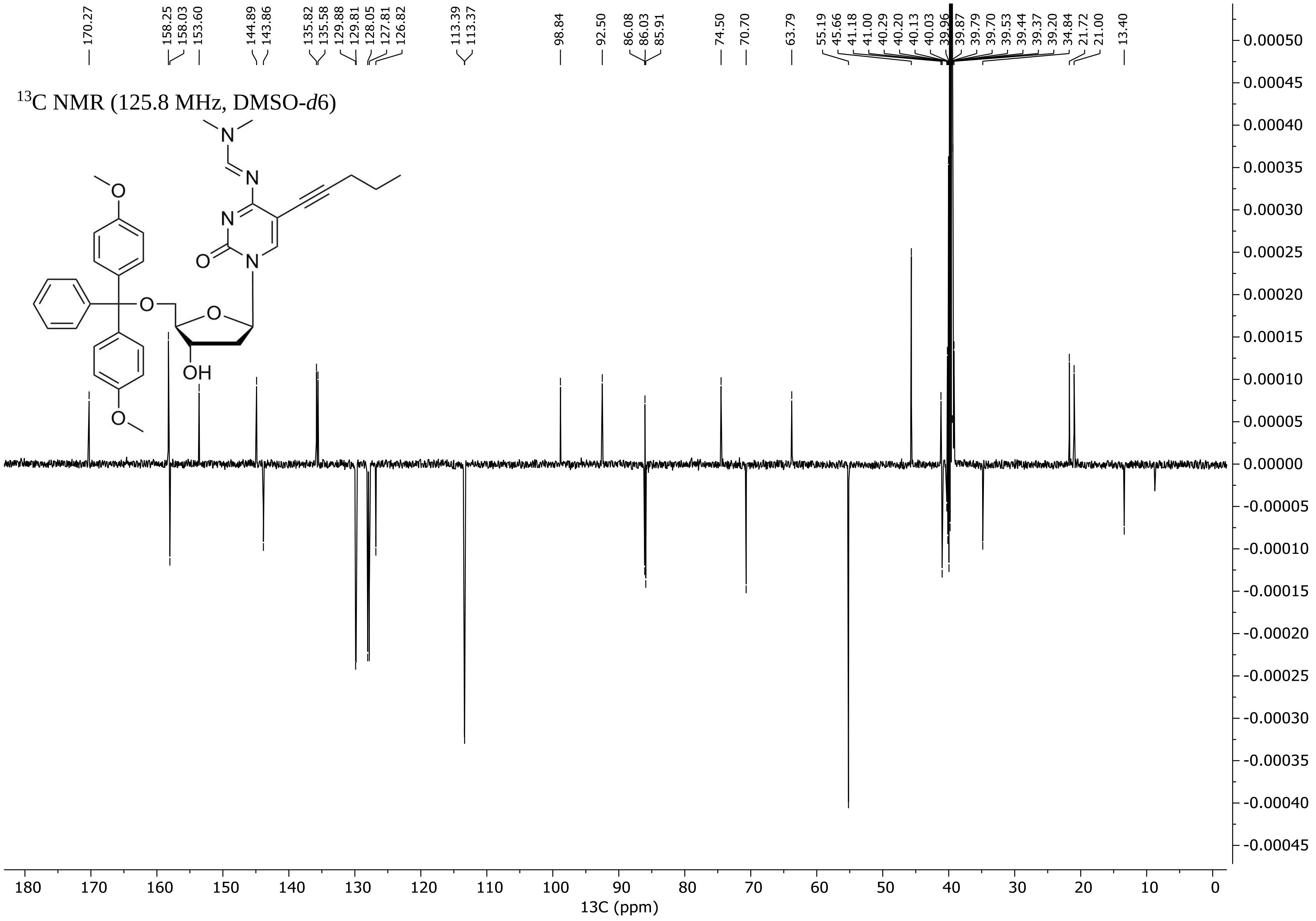

^{13}C NMR (125.8 MHz, DMSO-*d*6)
170.27
158.25
158.03
153.60
144.89
143.86
135.82
135.58
129.88
129.81
128.05
127.81
126.82
113.39
113.37
98.84
92.50
86.08
86.03
85.91
74.50
70.70
63.79
55.19
45.66
41.18
41.00
40.29
40.20
40.13
40.03
39.96
39.87
39.79
39.70
39.53
39.44
39.37
39.20
34.84
21.72
21.00
13.40
13C (ppm)

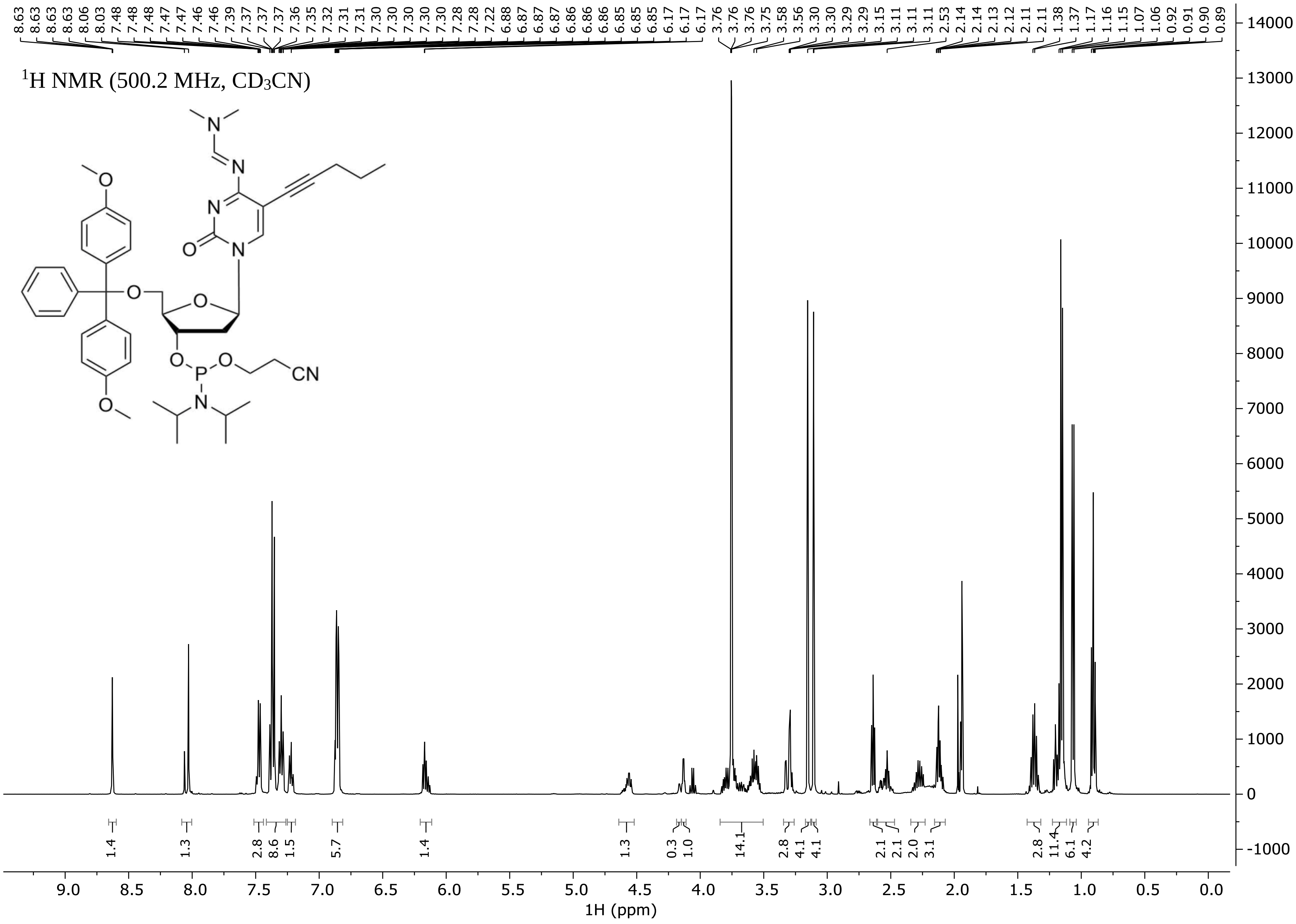

^{1}H NMR (500.2 MHz, CD_3CN)
1H (ppm)

^{13}C NMR (125.8 MHz, CD_3CN)

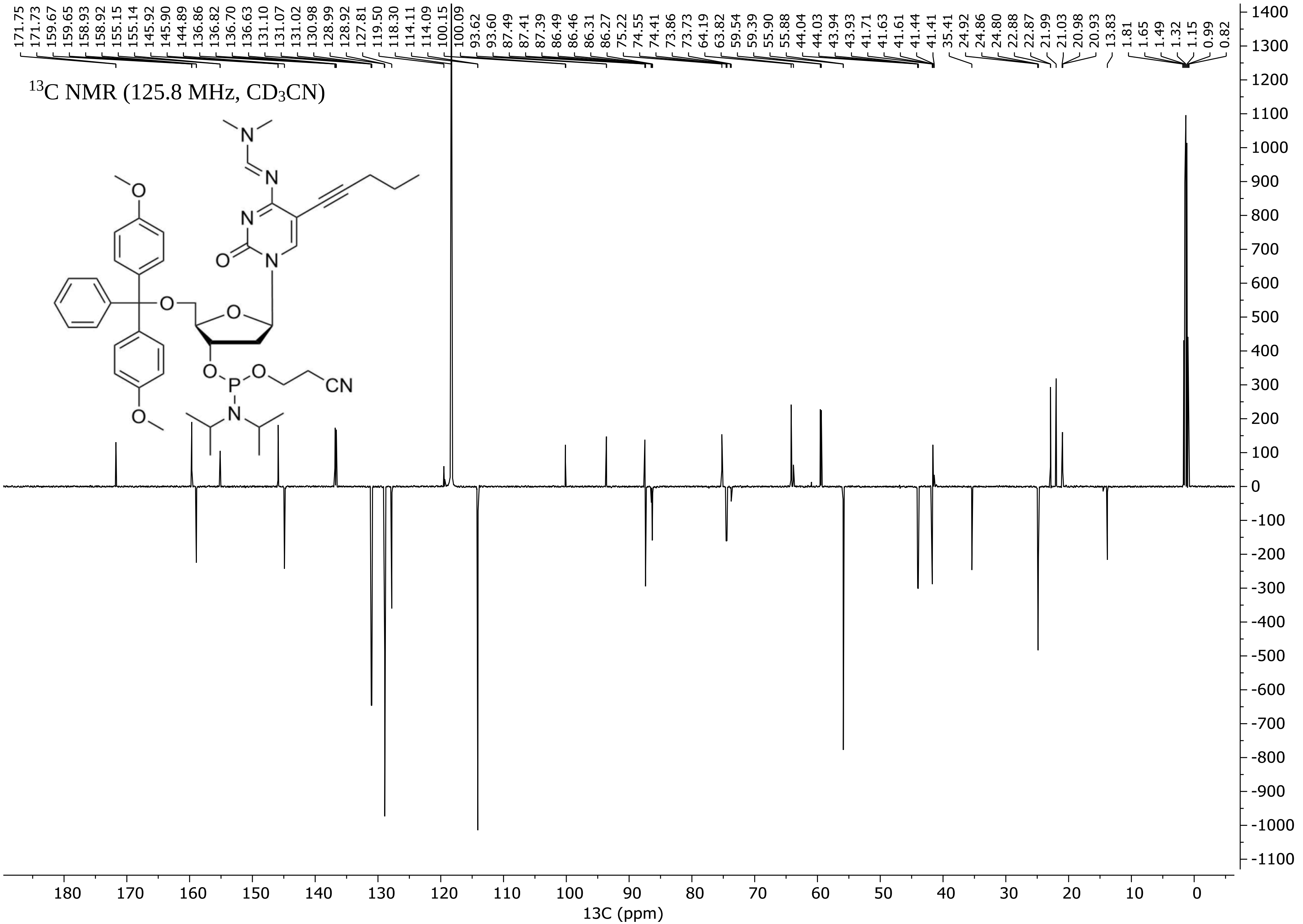

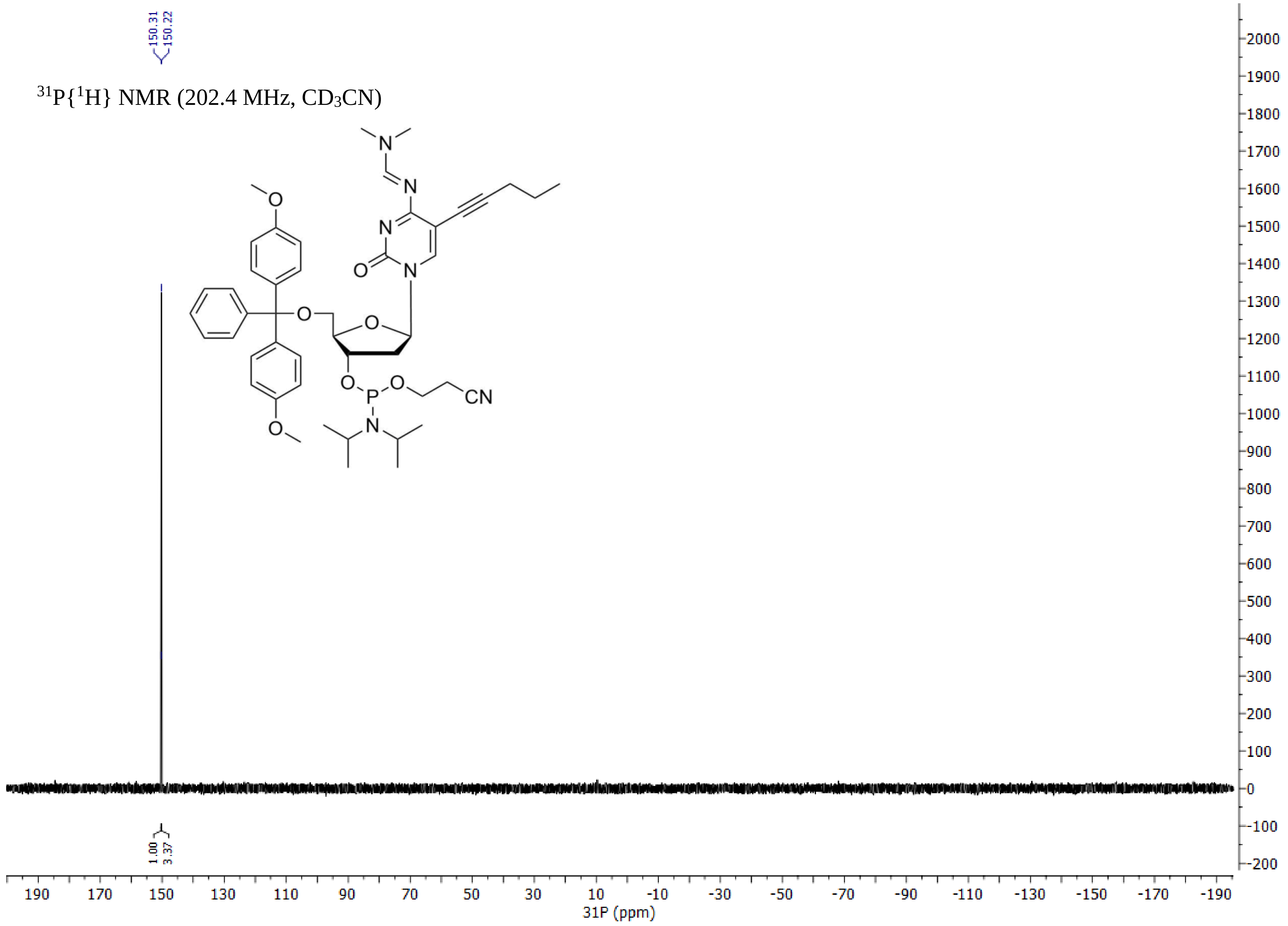
150.31
150.22
$^{31}P\{^{1}H\}$ NMR (202.4 MHz, CD_3CN)
1.00
3.37
31P (ppm)

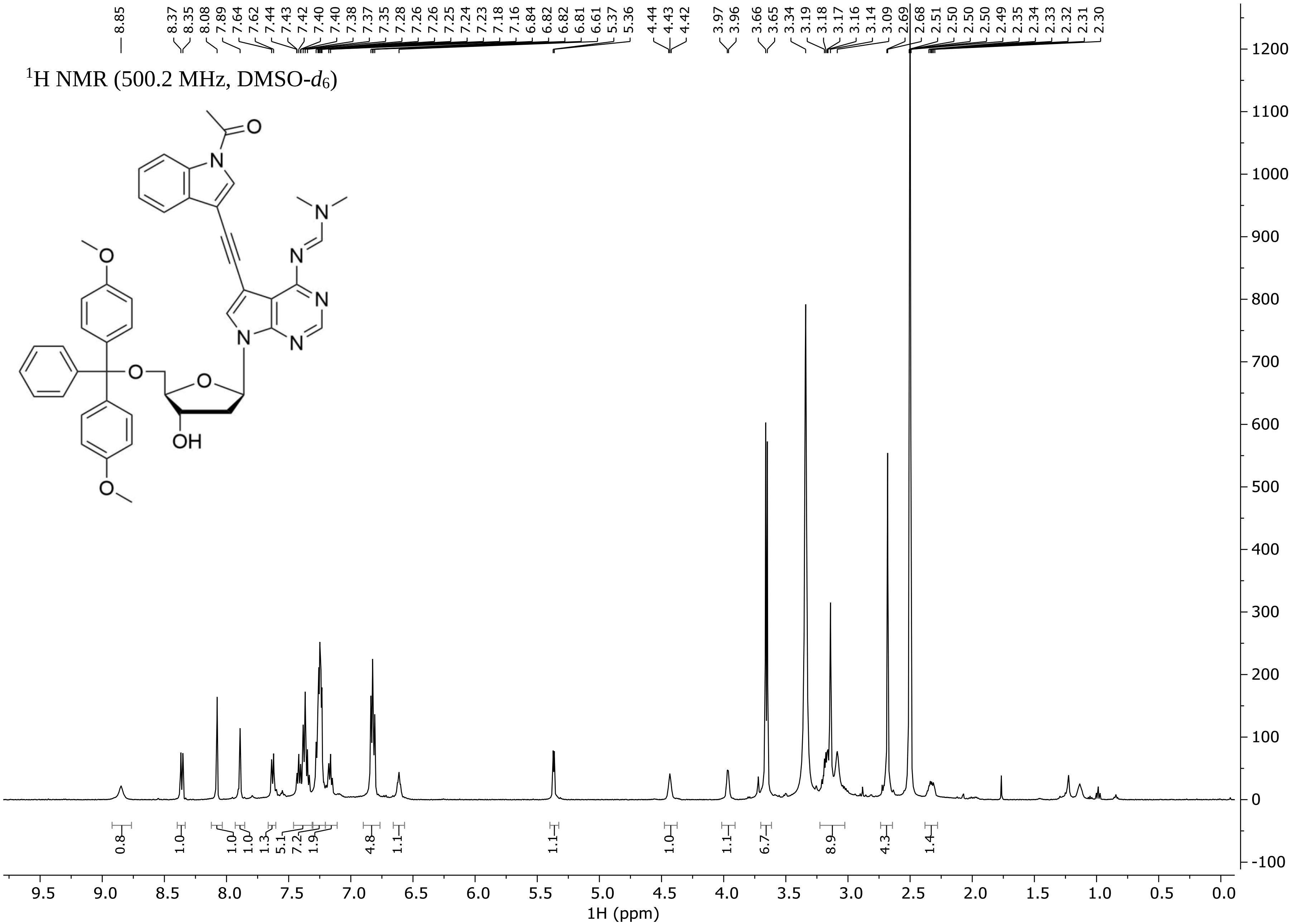

^{1}H NMR (500.2 MHz, DMSO-d_6)
1H (ppm)

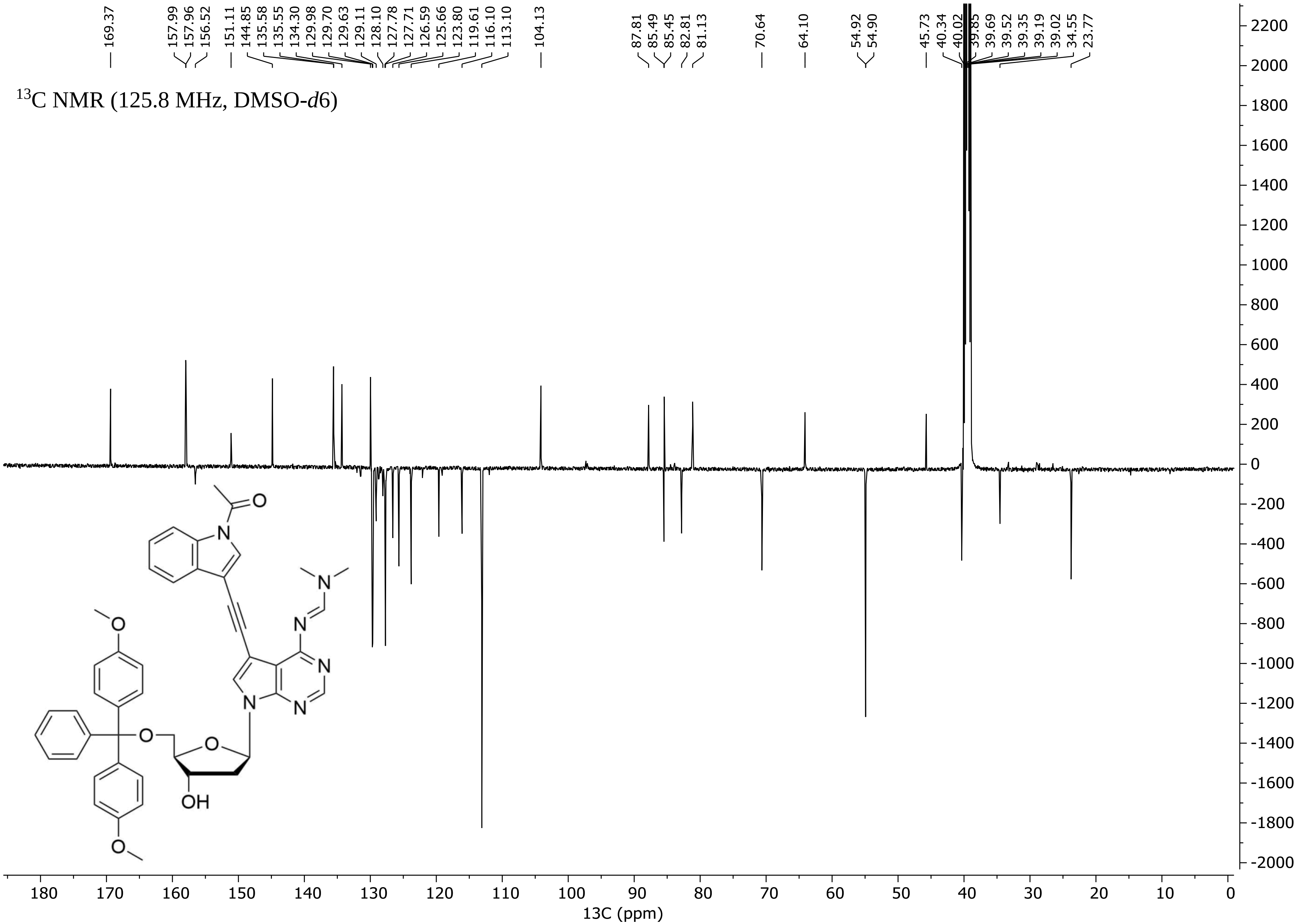

^{13}C NMR (125.8 MHz, DMSO-*d*6)
169.37
157.99
157.96
156.52
151.11
144.85
135.58
135.55
134.30
129.98
129.70
129.63
129.11
128.10
127.78
127.71
126.59
125.66
123.80
119.61
116.10
113.10
104.13
87.81
85.49
85.45
82.81
81.13
70.64
64.10
54.92
54.90
45.73
40.34
40.02
39.85
39.69
39.52
39.35
39.19
39.02
34.55
23.77
13C (ppm)

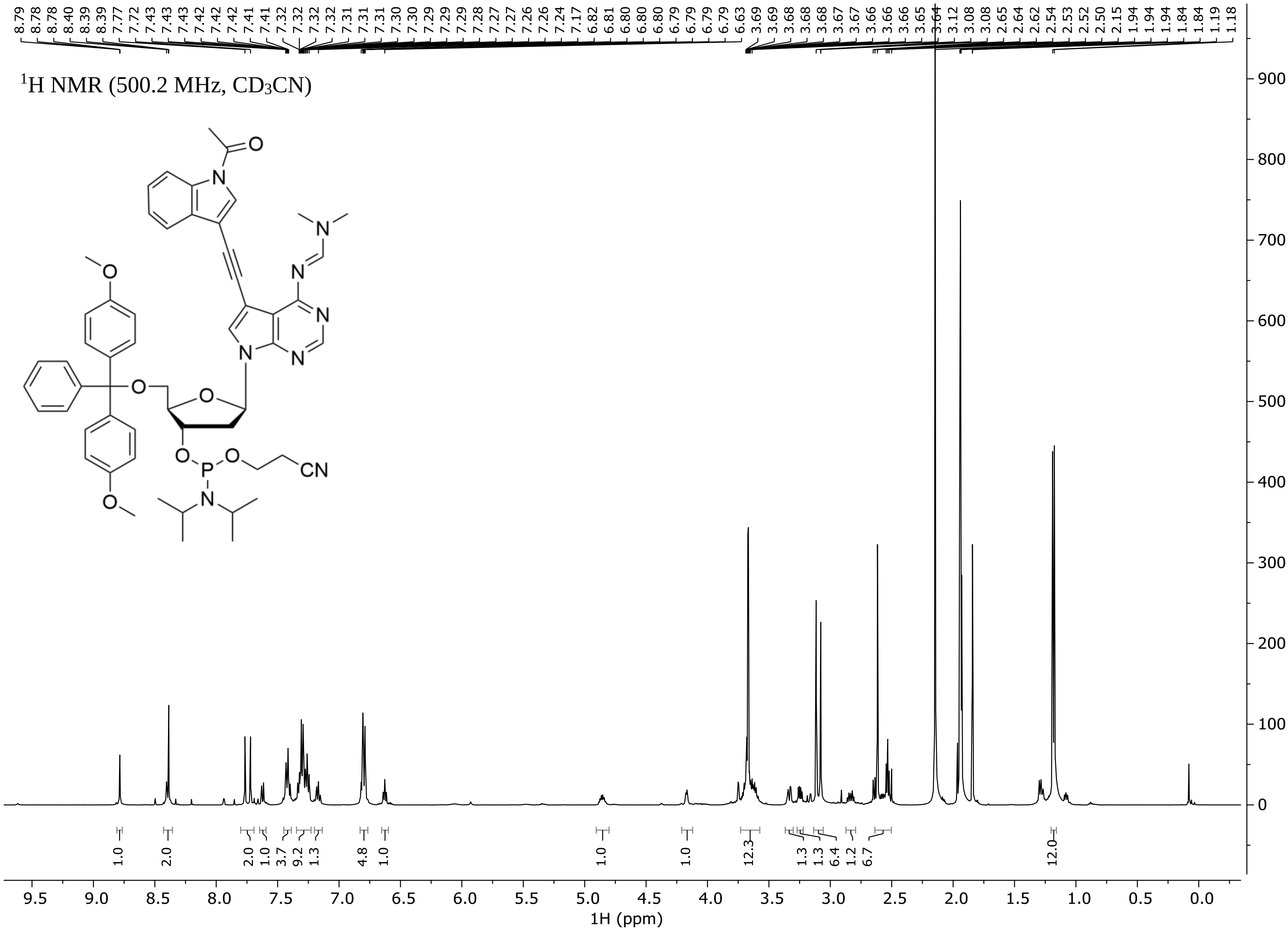

^{1}H NMR (500.2 MHz, CD_3CN)
1H (ppm)

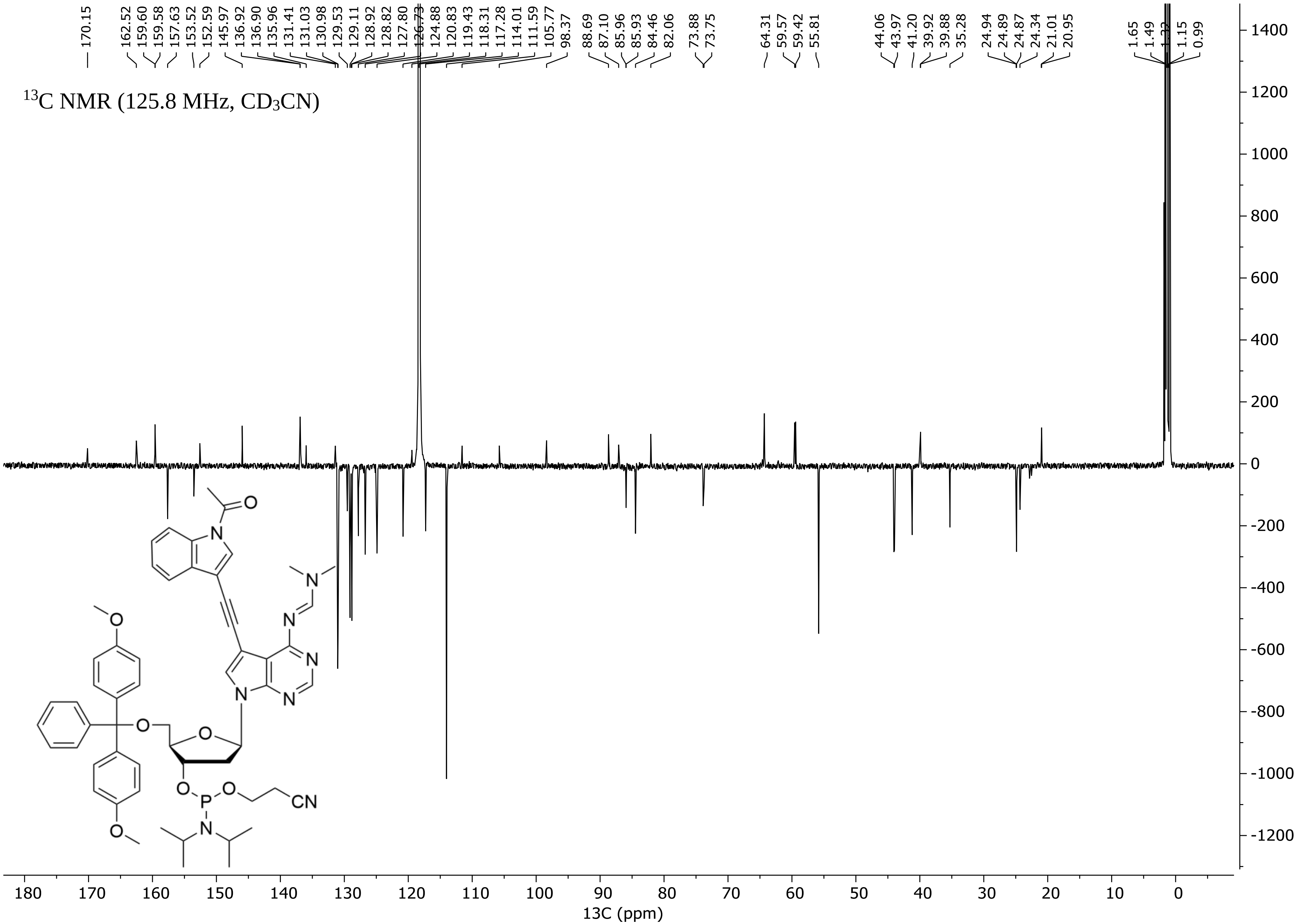

170.15
162.52
159.60
159.58
157.63
153.52
152.59
145.97
136.92
136.90
135.96
131.41
131.03
130.98
129.53
129.11
128.92
128.82
127.80
126.73
124.88
120.83
119.43
118.31
117.28
114.01
111.59
105.77
98.37
88.69
87.10
85.96
85.93
84.46
82.06
73.88
73.75
64.31
59.57
59.42
55.81
44.06
43.97
41.20
39.92
39.88
35.28
24.94
24.89
24.87
24.34
21.01
20.95
1.65
1.49
1.32
1.15
0.99
^{13}C NMR (125.8 MHz, CD_3CN)
13C (ppm)

$^{31}P\{^1H\}$ NMR (202.4 MHz, CD_3CN)

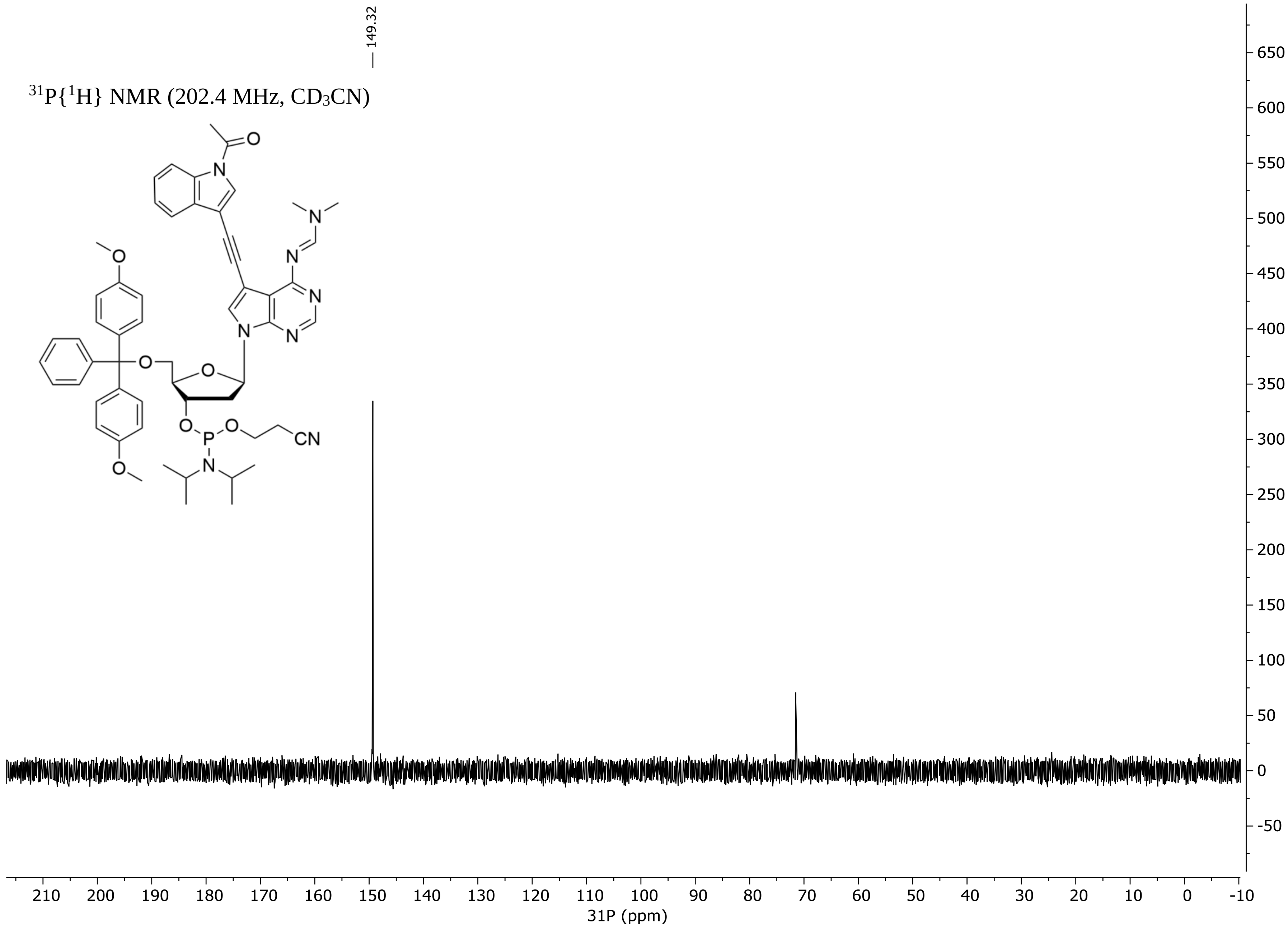

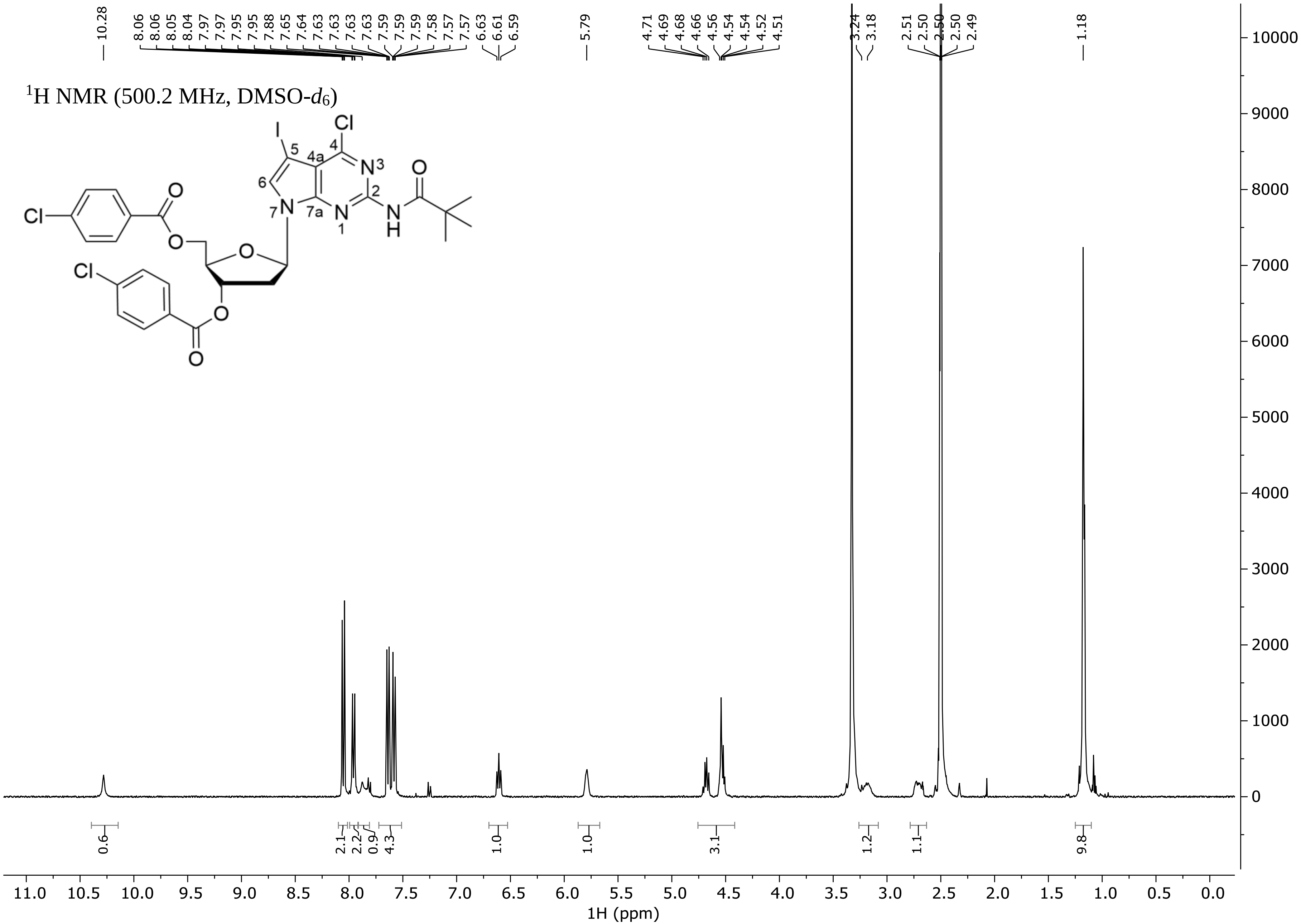

^{1}H NMR (500.2 MHz, DMSO-d_6)
10.28
8.06 8.06 8.05 8.04 7.97 7.97 7.95 7.95 7.88 7.65 7.64 7.63 7.63 7.63 7.63 7.59 7.59 7.59 7.58 7.57 7.57
6.63 6.61 6.59
5.79
4.71 4.69 4.68 4.66 4.56 4.54 4.54 4.52 4.51
3.24 3.18
2.51 2.50 2.50 2.50 2.49
1.18
0.6 2.1 2.2 0.9 4.3 1.0 1.0 3.1 1.2 1.1 9.8
1H (ppm)

^{13}C NMR (125.8 MHz, DMSO-*d*6)

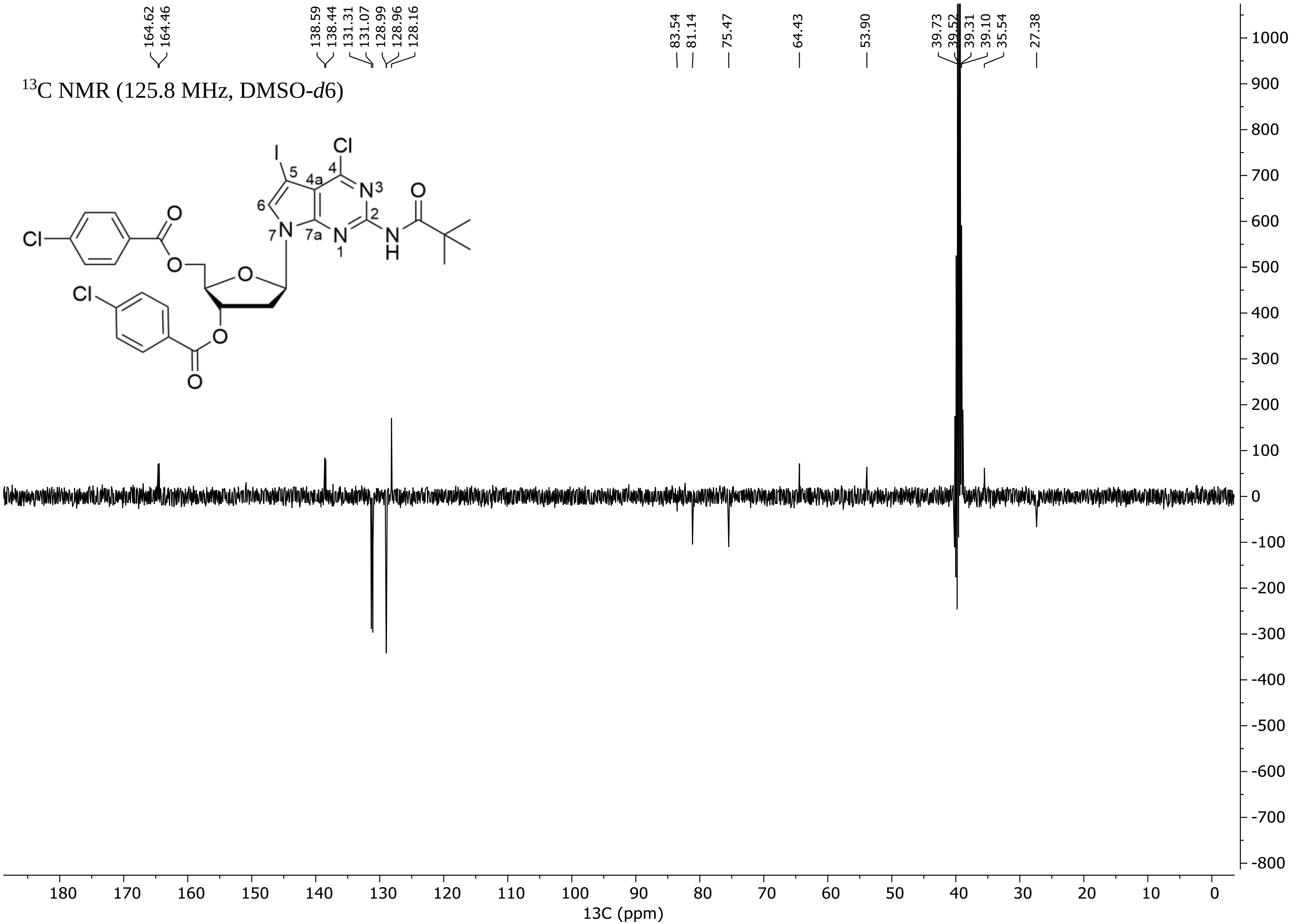

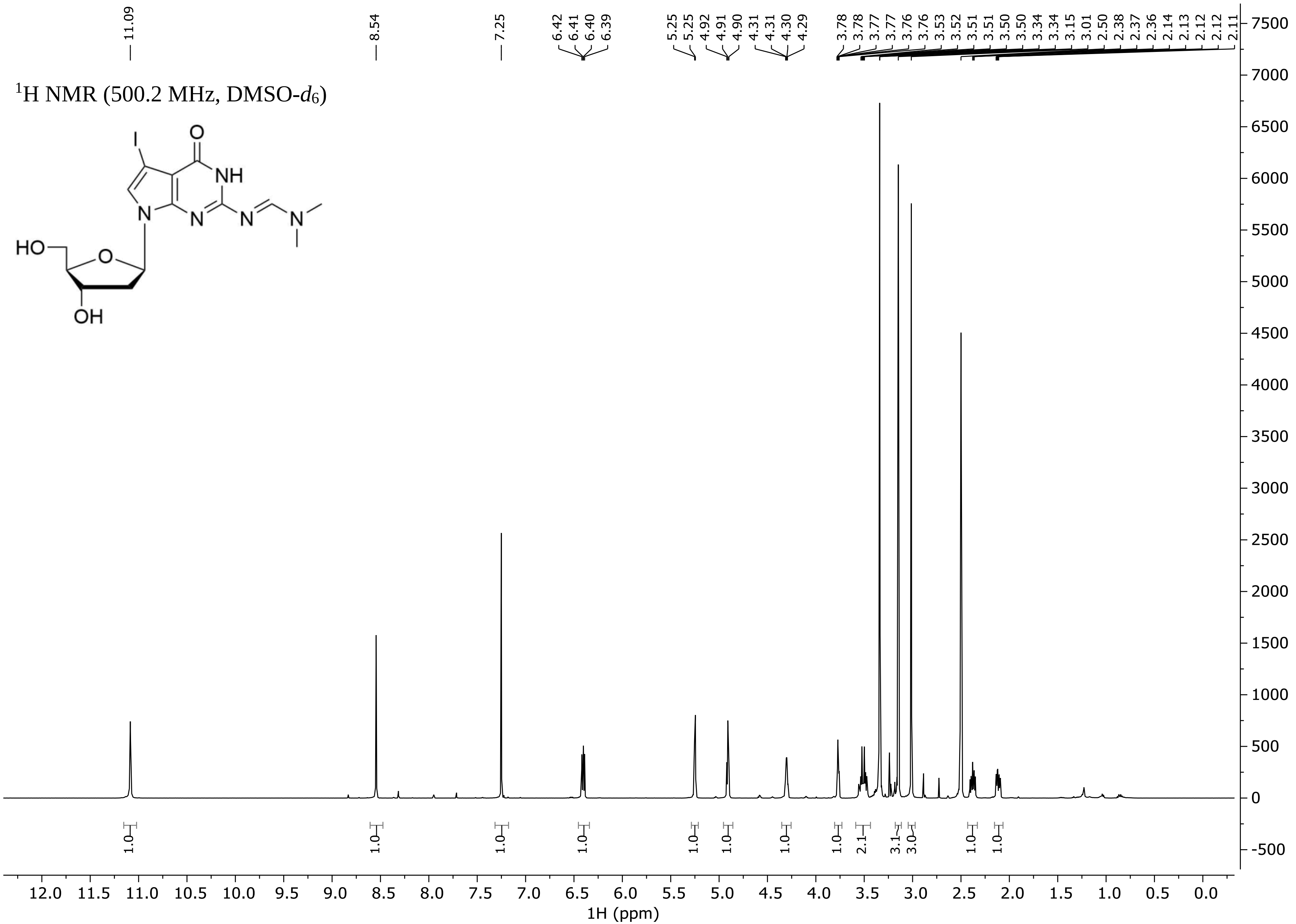

^{1}H NMR (500.2 MHz, DMSO-d_6)
1H (ppm)

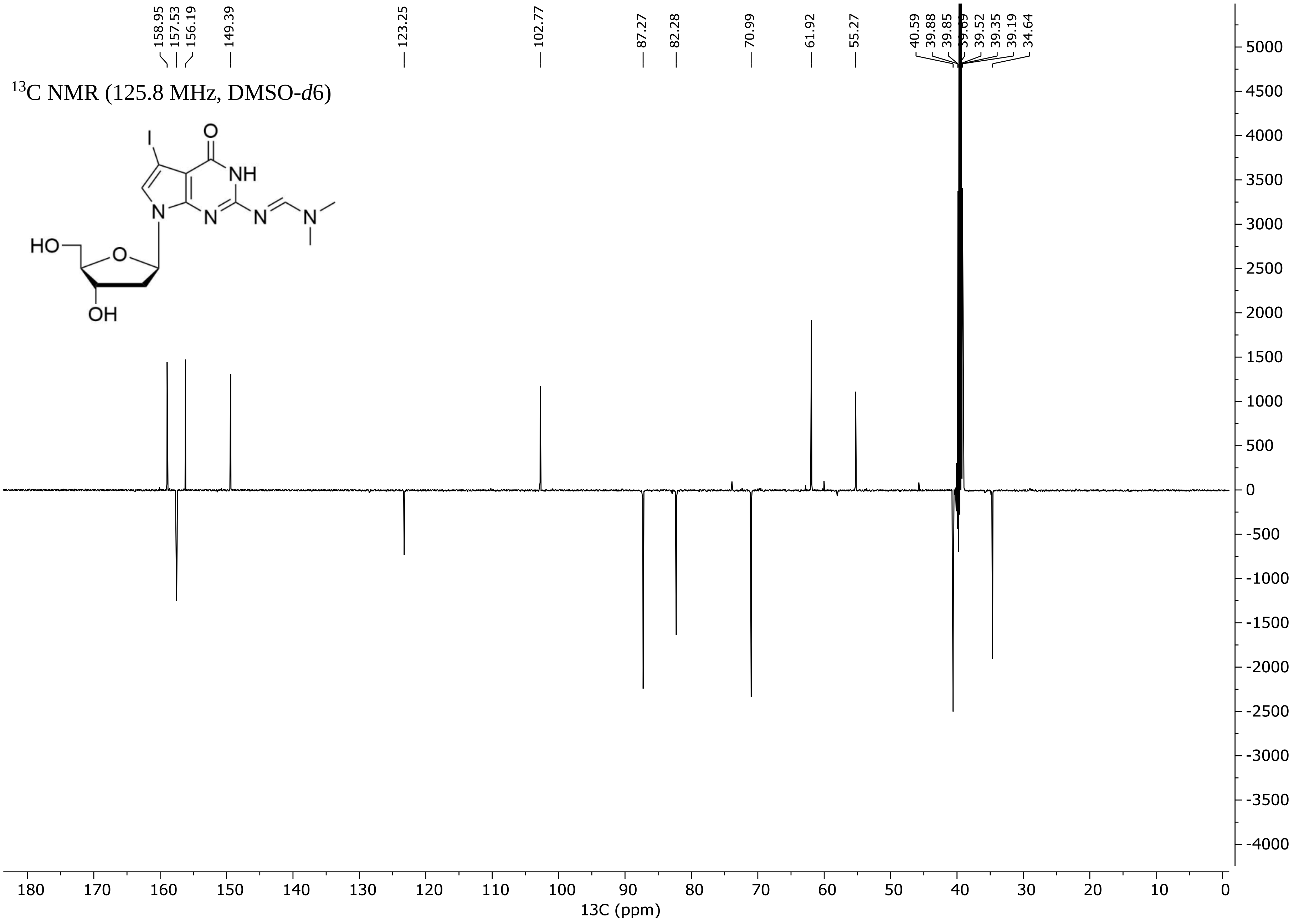
158.95
157.53
156.19
149.39
123.25
102.77
87.27
82.28
70.99
61.92
55.27
40.59
39.88
39.85
39.52
39.35
39.19
34.64
^{13}C NMR (125.8 MHz, DMSO-$d6$)
I
O
NH
N
N
N
N
HO
O
OH
5000
4500
4000
3500
3000
2500
2000
1500
1000
500
0
-500
-1000
-1500
-2000
-2500
-3000
-3500
-4000
180
170
160
150
140
130
120
110
100
90
80
70
60
50
40
30
20
10
0
13C (ppm)

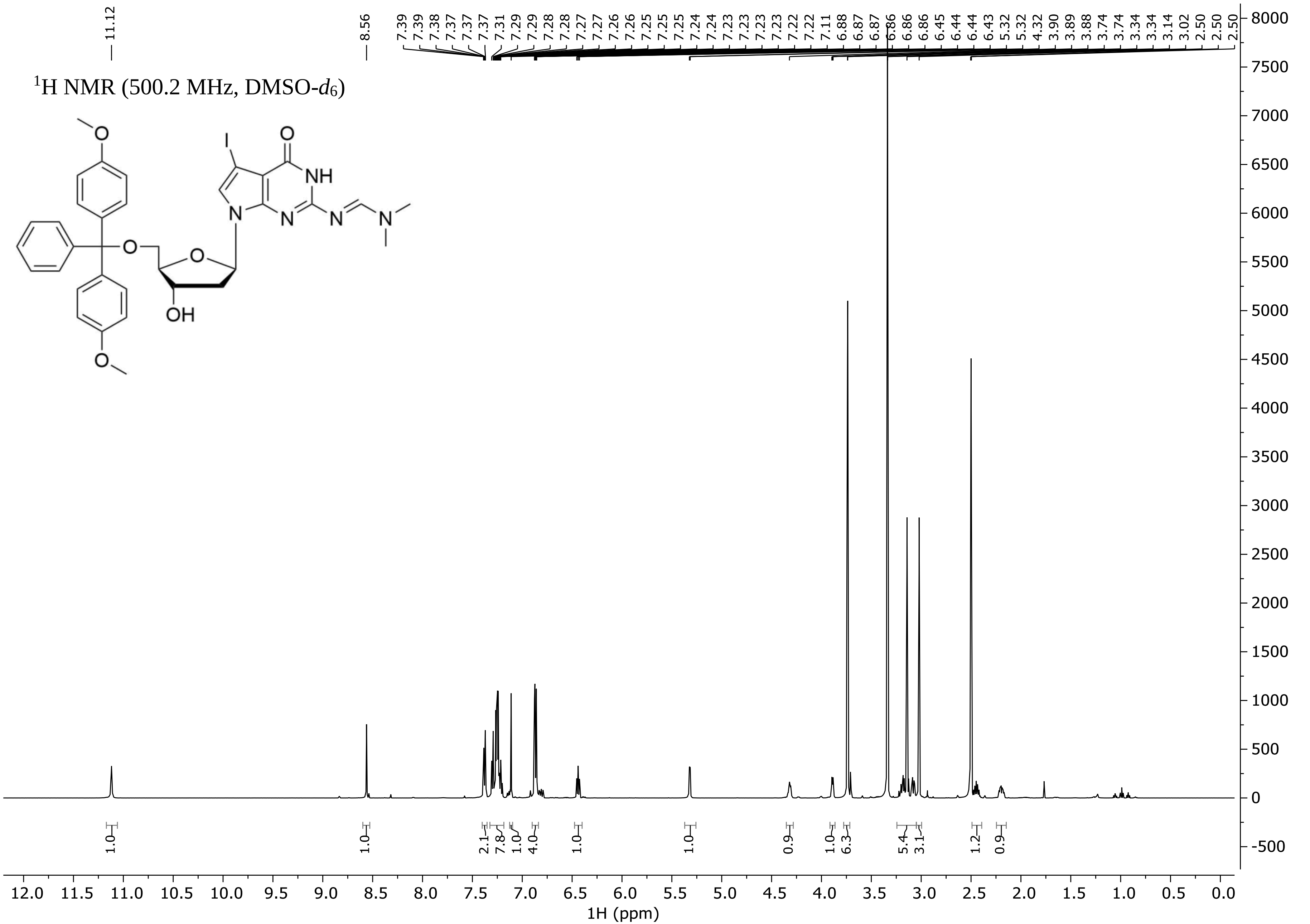
1H NMR (500.2 MHz, DMSO-d_6)
11.12
8.56
7.39 7.39 7.38 7.37 7.37 7.37 7.31 7.29 7.29 7.28 7.28 7.27 7.27 7.26 7.25 7.25 7.25 7.24 7.24 7.23 7.23 7.23 7.22 7.22 7.11 6.88 6.87 6.87 6.86 6.86 6.86 6.45 6.44 6.44 6.43 5.32 4.32 3.90 3.89 3.88 3.74 3.34 3.34 3.14 3.02 2.50 2.50
1.0 1.0 2.1 7.8 1.0 4.0 1.0 1.0 0.9 1.0 6.3 5.4 3.1 1.2 0.9
1H (ppm)

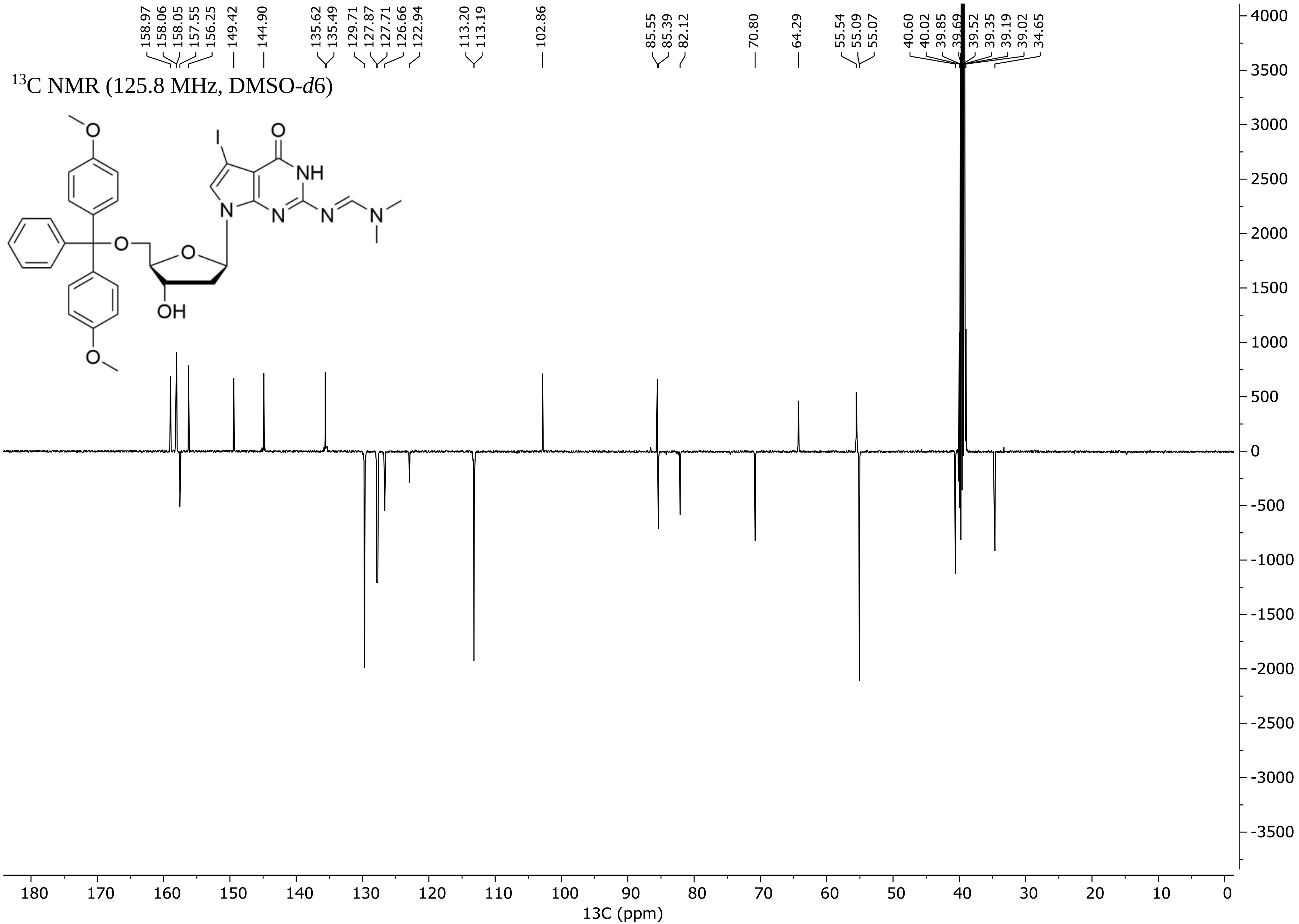
13C NMR (125.8 MHz, DMSO-d6)
158.97
158.06
158.05
157.55
156.25
149.42
144.90
135.62
135.49
129.71
127.87
127.71
126.66
122.94
113.20
113.19
102.86
85.55
85.39
82.12
70.80
64.29
55.54
55.09
55.07
40.60
40.02
39.85
39.69
39.52
39.35
39.19
39.02
34.65
I
O
NH
N
N
N
N
O
O
O
OH
O
13C (ppm)

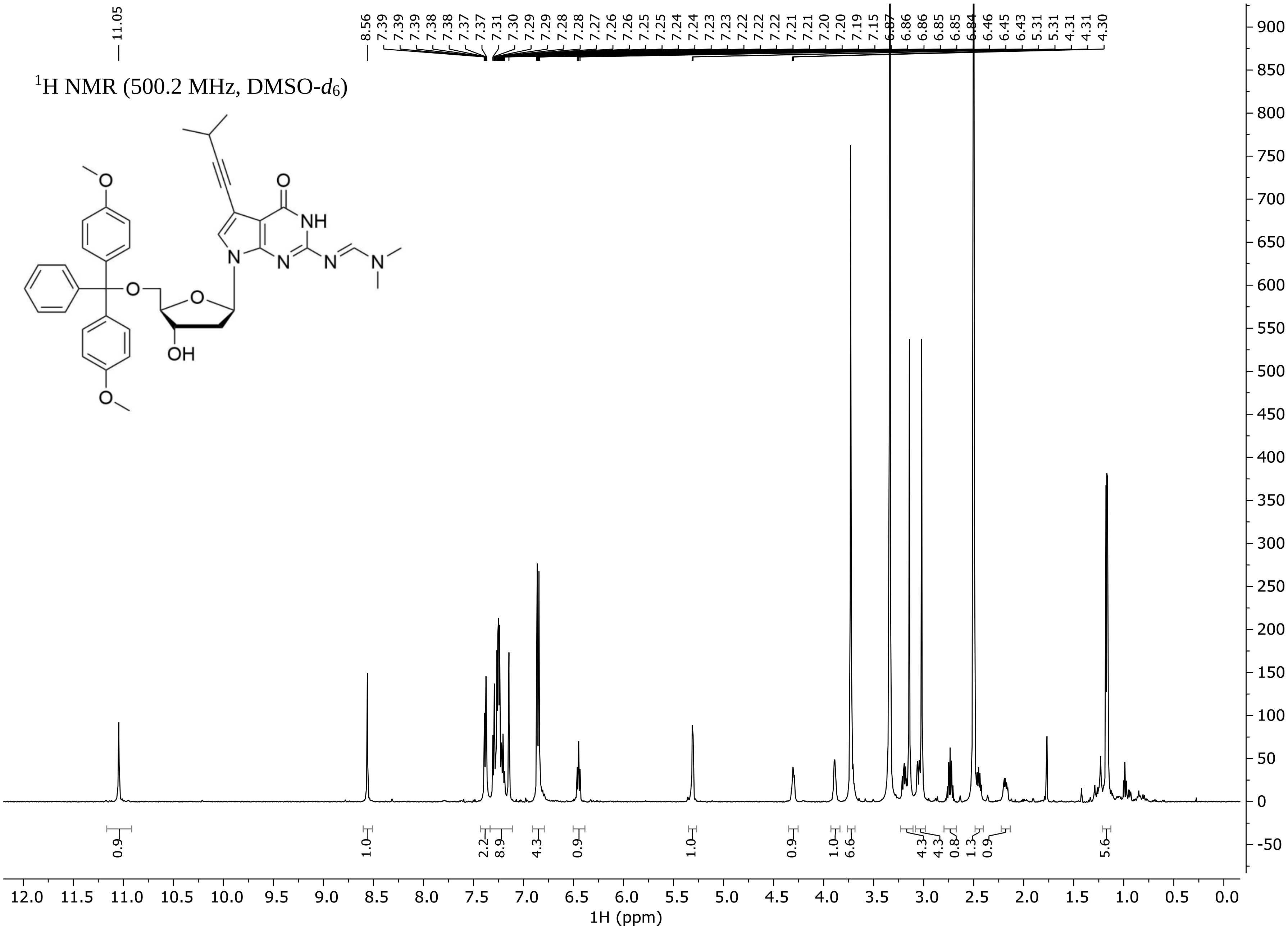
1H NMR (500.2 MHz, DMSO-d6)
1H (ppm)

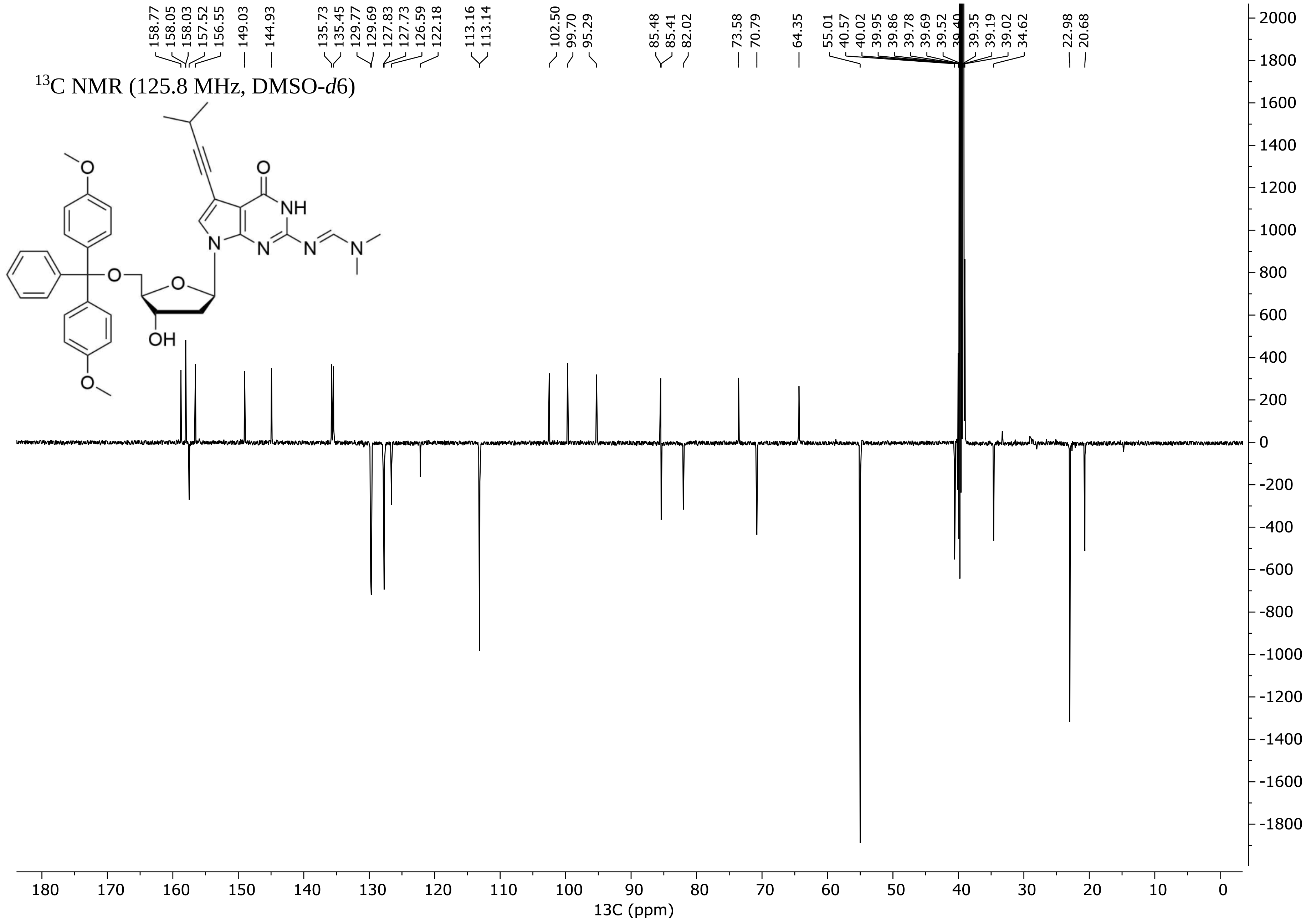

158.77
158.05
158.03
157.52
156.55
149.03
144.93
135.73
135.45
129.77
129.69
127.83
127.73
126.59
122.18
113.16
113.14
102.50
99.70
95.29
85.48
85.41
82.02
73.58
70.79
64.35
55.01
40.57
40.02
39.95
39.86
39.78
39.69
39.52
39.35
39.19
39.02
34.62
22.98
20.68
^{13}C NMR (125.8 MHz, DMSO-*d*6)
13C (ppm)

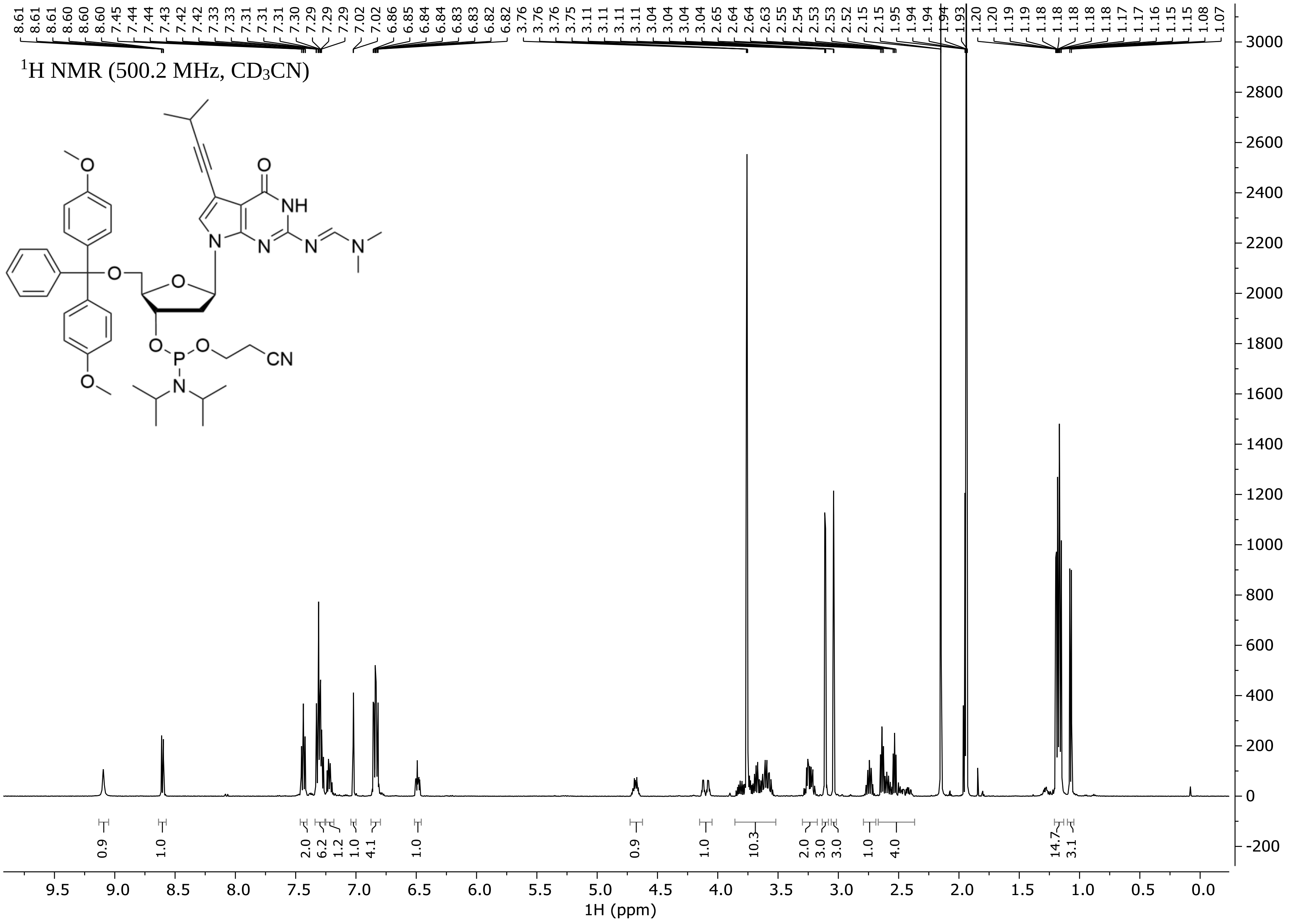

1H NMR (500.2 MHz, CD3CN)
1H (ppm)

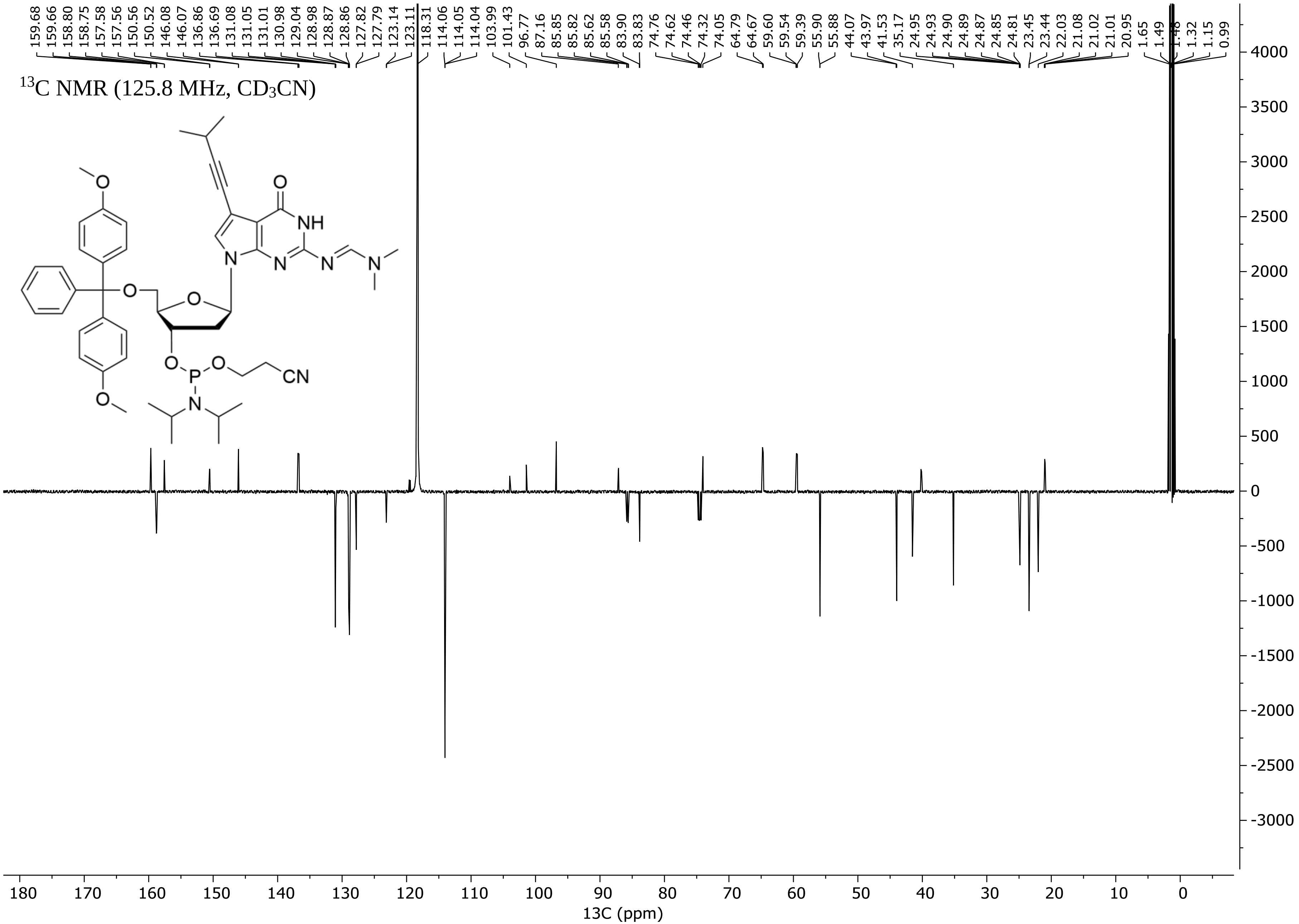

^{13}C NMR (125.8 MHz, CD_3CN)
159.68
159.66
158.80
158.75
157.58
157.56
150.56
150.52
146.08
146.07
136.86
136.69
131.08
131.05
131.01
130.98
129.04
128.98
128.87
128.86
127.82
127.79
123.14
123.11
118.31
114.06
114.05
114.04
103.99
101.43
96.77
87.16
85.85
85.82
85.62
85.58
83.90
83.83
74.76
74.62
74.46
74.32
74.05
64.79
64.67
59.60
59.54
59.39
55.90
55.88
44.07
43.97
41.53
35.17
24.95
24.93
24.90
24.89
24.87
24.85
24.81
23.45
23.44
22.03
21.08
21.02
21.01
20.95
1.65
1.49
1.48
1.32
1.15
0.99
NH
N
N
N
N
O
O
O
O
O
O
O
P
N
CN
13C (ppm)

$^{31}P\{^{1}H\}$ NMR (202.4 MHz, CD_3CN)

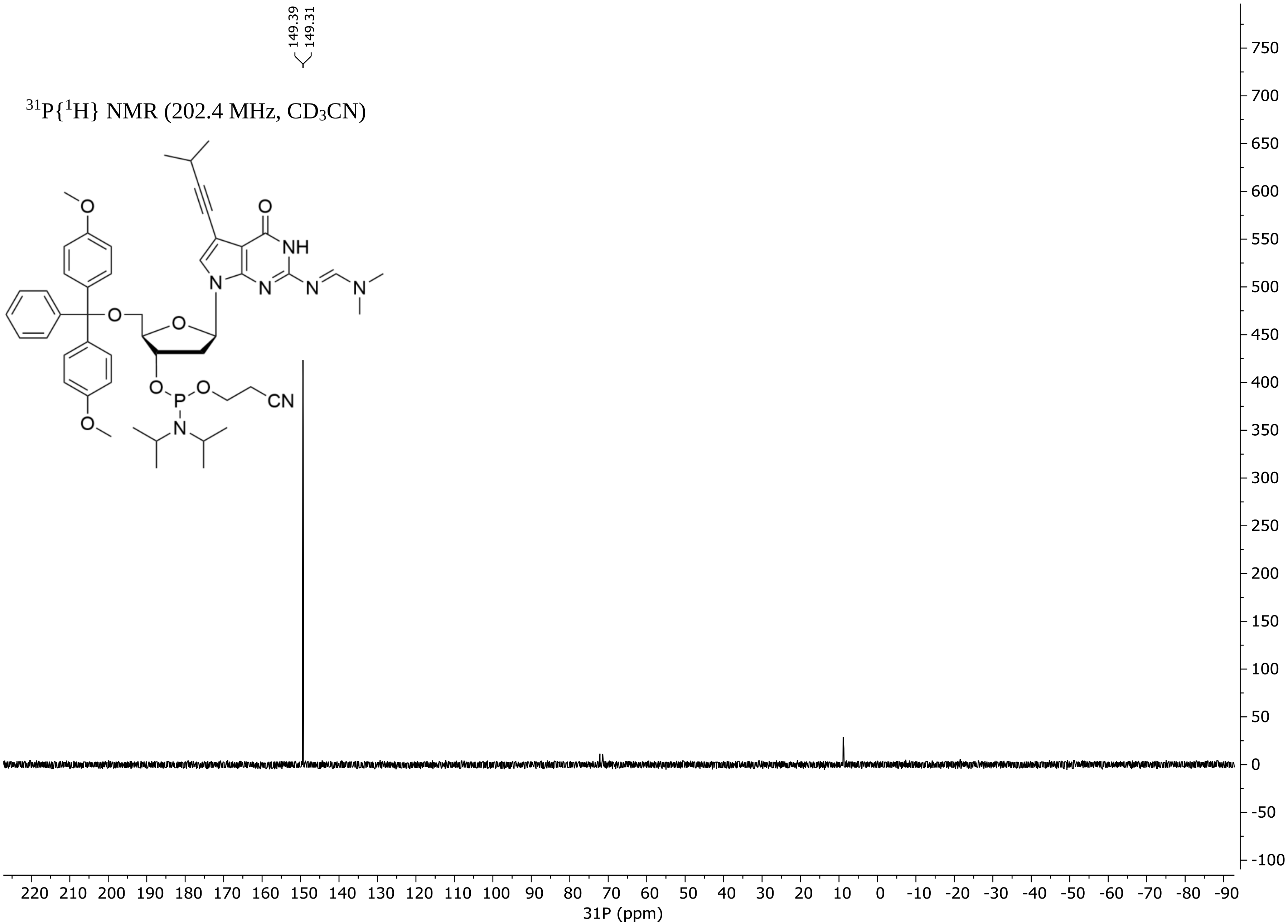

5 Reference List

(1) Marty, M. T.; Baldwin, A. J.; Marklund, E. G.; Hochberg, G. K.; Benesch, J. L.; Robinson, C. V. Bayesian deconvolution of mass and ion mobility spectra: from binary interactions to polydisperse ensembles. *Anal. Chem.* **2015**, *87*, 4370-6.

(2) Ondruš, M.; Sýkorová, V.; Bednárová, L.; Pohl, R.;Hocek, M. Enzymatic synthesis of hypermodified DNA polymers for sequence-specific display of four different hydrophobic groups. *Nucleic Acids Res.* **2020**, *48*, 11982-11993.

(3) Zhang, C.-P.; Wang, Z.-L.; Chen, Q.-Y.; Zhang, C.-T.; Gu, Y.-C.; Xiao, J.-C. Copper-Mediated Trifluoromethylation of Heteroaromatic Compounds by Trifluoromethyl Sulfonium Salts. *Angew. Chem. Int. Ed*. **2011**, *50*, 1896-1900.

(4) Iida, K.; Ishida, S.; Watanabe, T.; Arai, T. Disulfide-Catalyzed Iodination of Electron-Rich Aromatic Compounds. *J. Org. Chem.* **2019**, *84*, 7411-7417.

(5) Sánchez-Cantalejo, F.; Priest, J. D.; Davies, P. W. A Gold Carbene Manifold to Prepare Fused γ-Lactams by Oxidative Cyclisation of Ynamides. *Chem. Eur. J.* **2018**, *24*, 17215-17219.

(6) Matarazzo, A.; Brow, J.; Hudson, R. H. E. Synthesis and photophysical evaluation of new fluorescent 7-arylethynyl-7-deazaadenosine analogs. *Can. J. Chem.* **2018**, *96*, 1093-1100.

(7) Seela, F.; Peng, X. Pyrrolo[2,3-d]pyrimidine β-L-Nucleosides Containing 7-Deazaadenine, 2-Amino-7-deazaadenine, 7-Deazaguanine, 7-Deazaisoguanine, and 7-Deazaxanthine. Collect. *Czech. Chem. Commun.* **2006**, *71*, 956-977.

(8) Knapp, D. C.; Serva, S.; D'Onofrio, J.; Keller, A.; Lubys, A.; Kurg, A.; Remm, M.; Engels, J. W. Fluoride-cleavable, fluorescently labelled reversible terminators: synthesis and use in primer extension. *Chem. Eur. J.* **2011**, *17*, 2903-15.

(9) Seela, F.; Zulauf, M. 7-Deazaadenine-DNA: Bulky 7-Iodo Substituents or Hydrophobic 7-Hexynyl Chains Are Well Accommodated in the Major Groove of Oligonucleotide Duplexes. *Chem. Eur. J.* **1998**, *4*, 1781-1790.

(10) Seela, F.; Peng, X. Regioselective Syntheses of 7-Halogenated 7-Deazapurine Nucleosides Related to 2-Amino-7-deaza-2′-deoxyadenosine and 7-Deaza-2′-deoxyisoguanosine. *Synthesis.* **2004**, 1203-1210.

(11) Seela, F.; Shaikh, K. 7-halogenated 7-deaza-2'-deoxyxanthine 2'-deoxyribonucleosides. *Helv. Chim. Acta.* **2004**, *87*, 1325-1332.

(12) Ramzaeva, N.; Seela, F. 7-Substituted 7-Deaza-2′-deoxyguanosines: Regioselective Halogenation of Pyrrolo[2,3-d]pyrimidine Nucleosides. *Helv. Chim. Acta.* **1995**, *78*, 1083-1090.
